# Metal-free regioselective mono- and poly-halogenation of 2-substituted indazoles†

**DOI:** 10.1039/d2ra07398f

**Published:** 2023-02-07

**Authors:** Changjun Zhang, Tingting Wei, Zhichen Yu, Yuxin Ding, Weike Su, Yuanyuan Xie

**Affiliations:** a College of Pharmaceutical Sciences, Zhejiang University of Technology Hangzhou 310014 P. R. China xyycz@zjut.edu.cn; b Collaborative Innovation Center of Yangtze River Delta Region Green Pharmaeuticals, Zhejiang University of Technology Hangzhou 310014 P. R. China; c Key Laboratory of Pharmaceutical Engineering of Zhejiang Province Hangzhou 310014 China

## Abstract

An unprecedented metal-free regioselective halogenation of 2*H*-indazoles has been revealed, which not only realized the highly selective synthesis of mono-halogenated products, but also completed poly-halogenations by fine tuning the reaction conditions. Various mono-/poly-/hetero-halogenated indazoles were obtained in moderate to excellent yields. Notably, this approach features environmentally friendly solvents, mild reaction conditions, simple execution and short reaction time.

## Abstract 2

An unprecedented metal-free regioselective halogenation of 2*H*-indazoles is reported, and not only realizes the highly selective synthesis of mono-halogenated products, but also complete poly-halogenations.

## Graphical abstract

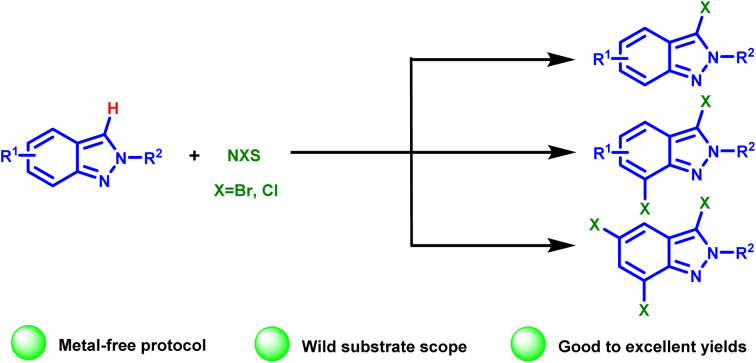

Halogens can significantly alter the biological properties of molecules, rendering the use of these compounds as drugs, agrochemicals, biocides, *etc.*^1^ In addition, organic halides are one of the most widely used precursors or intermediates for numerous organic transformations.^2^ For example, hetero-aromatic bromides and iodides play an important role in Grignard reactions^3^ and cross-coupling.^4^ Therefore, the construction of halogenated hetero-aromatic compounds through direct C–H halogenation is highly desirable.

Indazole, a nitrogen-containing heterocycle, has attracted much attention for its biological properties and a broad spectrum of medicinal values,^5^ such as anti-ovarian cancer drug Niraparib,^6^ selective estrogen receptor degrading agents,^7^ liver X receptor agonist,^8^ selective CRAF inhibitor,^9^ anticancer drugs Pazopanib,^10^ MK-482714,^9^ and gamedazoleq^11^ (Fig. 1). Notably, these drugs could be synthesized from halogenated indazole intermediates.

**Fig. 1:**
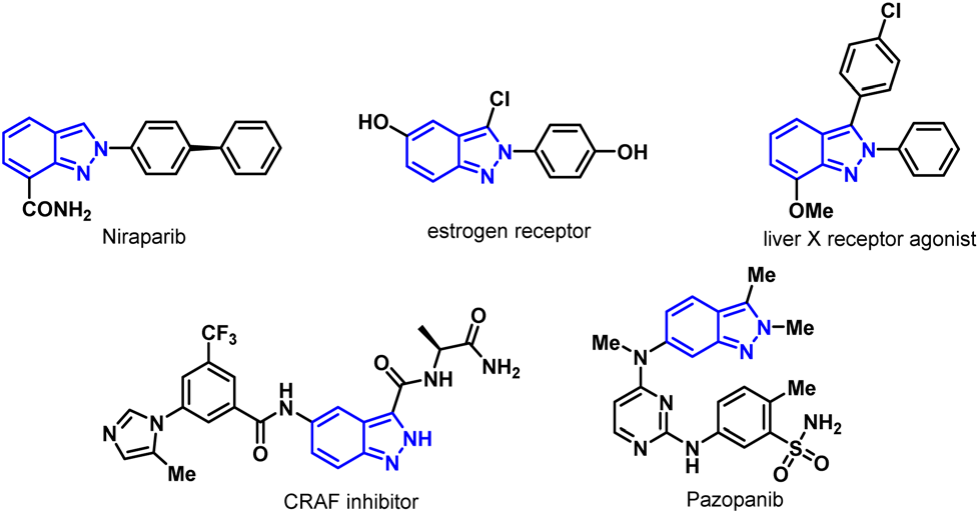
Bioactive compounds containing 2*H*-indazole.

Recognizing the importance of these molecules, chemists have developed various methods to synthesize indazole halides. However, C–H direct bromination of indazoles without metal catalysts has been rarely reported. Clarisse declared the bromination of 2-phenyl-2*H*-indazole employing Br_2_ as brominating reagent.^12^ Although 3-bromo-2*H*-indazole was formed in high yield, a mixture of 3,5-dibromo- and 3,7-dibromo-2*H*-indazole was obtained with poor selectivity and low yield. At the same time, the use of Br_2_ was environmentally unfriendly and troublesome. Herein, an efficient C–H direct halogenation of 2*H*-indazoles employing NXS (X = Br, Cl) was reported, which achieved the selective synthesis of mono-, poly- and hetero-halogenated products in high yields by adjusting reaction conditions.

In our initial study, 2-phenyl-2*H*-indazole (1a) and NBS (1.0 equiv.) were selected as model substrates to react at 25 °C. It was delighted that 88% mono-brominated product 2a was obtained after 2.0 h (Table 1, entry 1). Preliminary investigation of the reaction temperature demonstrated that the yield of target product 2a increased to 98% with the increase of reaction temperature (Table 1, entry 2). Similarly, the screening of solvents was also within our consideration for the purpose of corresponding green chemistry. Switching the MeCN to H_2_O or CH_3_OH, led to the decreased yield of 2a (Table 1, entries 3 and 4). But gratifyingly, in green solvent EtOH, 1a could be cleanly converted into mono-substituted product 2a with an excellent yield of 97% (Table 1, entry 5). The reaction temperature and equiv. of NBS were further investigated when H_2_O was used as solvent. The result indicated that in the presence of 1.3 equiv. NBS, it was suitable for mono-bromination and 2a was isolated by simple filtration with high yield of 96% under 95 °C (Table 1, entry 6). To our surprise, gradually increasing the equiv. of NBS produced disubstituted products 3,7-dibromo-2*H*-indazole 3a (Table 1, entry 7). The yield of 3a was greatly improved, when H_2_O was replaced by EtOH or MeCN, but higher temperature seemed to have a detrimental effect (Table 1, entries 8–10). It was worth to mention that no byproduct 3,5-dibromo-2*H*-indazole was detected. This reaction was then carried out at 80 °C by increasing the equiv. of NBS, suggesting that trisubstitution was best performed at 4.0 equiv. of NBS in MeCN and the yield of tribrominated product 4a could be increased to 71% (Table 1, entry 13).

**Table 1:** Screening of reaction parameters^a^

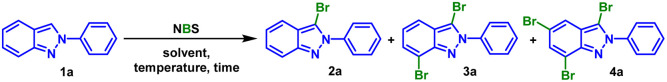				
Entry	Solvent	NBS (equiv.)	*T* (°C)	Yield^b^2a : 3a : 4a
1	MeCN	1.0	25	88 : 0 : 0
2	MeCN	1.0	50	98 : 0 : 0
3	CH_3_OH	1.0	50	92 : 0 : 0
4	H_2_O	1.0	50	75 : 0 : 0
**5**	**EtOH**	**1.0**	**50**	**97 : 0 : 0**
**6** c	**H**_**2**_ **O**	**1.3**	**95**	**96 : 0 : 0**
7^d^	H_2_O	2.0	50	80 : 5 : 0
**8** e	**EtOH**	**2.0**	**50**	**23 : 67 : trace**
9^e^	MeCN	2.0	50	35 : 59 : trace
10^e^	EtOH	2.0	80	32 : 55 : 5
11^d^	EtOH	3.0	80	10 : 25 : 56
12^d^	EtOH	4.0	80	8 : 24 : 67
**13** f	**MeCN**	**4.0**	**80**	**3 : 20 : 71**

a Reaction conditions: 1a (0.3 mmol), NBS (0.3 mmol) in 3.0 mL solvent, *T*, 2 h.

b Isolated yields.

c 5 h.

d 6 h.

e Adding NBS in batches into 5.0 mL solvent, 6 h.

f Dropwising 4.0 mL NBS (aq.) to the solution of 1a (1.0 mL), 8 h.

With the mentioned optimized reaction protocol in hand, first of all, the scopes of the mono-bromination were examined (Table 2). The effects of different substituents on the *N*-phenyl ring of 2*H*-indazoles were investigated, and the desired products could be obtained in the yield of 80–98% for both electron-donating and electron-withdrawing groups (2a–2l). Steric hindrance had effect on the yield, *m*-substituents on the phenyl ring resulting in lower yields compared to *p*-substituents (2b and 2g), and 3,4-disubstituents on the phenyl ring furnishing the desired products in moderate yields (2m and 2n). However, the situation changed when the substituents was on the indazole skeleton. It was found that electron-withing groups such as F or Cl were compatible with the optimized reaction conditions and afforded the corresponding desired products in good to excellent yields (2p*vs.*2q). While the substituent was methoxy, the raw material could not be completely converted giving product in 31% yield (2o). Furthermore, this method could be extended to the mono-bromination of *N*-pyridyl indazole with 81% yield (2r). In addition, applicability of aliphatic substituted substrates was also explored. The yield decreased sharply to 36% when the substituent was *tert*-butyl (2s), while none product was detected with *n*-butyl substituted indazole (2r).

**Table 2:** Substrate scope for mono-bromination of 2*H*-indazoles^a^^,^^b^

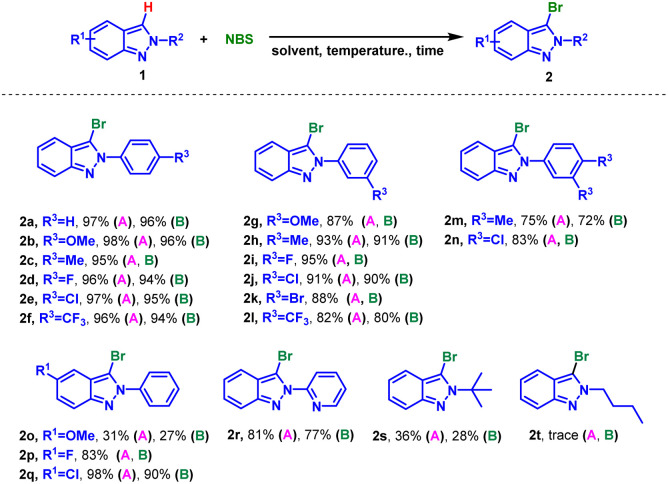

a Reaction conditions: (A) 1 (0.3 mmol), NBS (0.3 mmol), EtOH (3.0 mL), 50 °C, air, 2.0 h. (B) 1 (0.3 mmol), NBS (0.39 mmol), H_2_O (3.0 mL), 95 °C, air, 5.0 h.

b Isolated yield.

Inspired by the successful mono-bromination of 2*H*-indazoles under environmentally friendly conditions, the mono-chlorination was subsequently tested using NCS as chlorinating reagent (Table 3). The substrates with substituents on *N*-phenyl ring or indazole skeleton exhibited good reactivity both in H_2_O and EtOH (5a–5r). Interestingly, *m*-substituents on the phenyl ring gave higher yields than *p*-substituents, as opposed to mono-bromination (5b*vs.*5h). 2-Pyridyl-2*H*-indazole and 2-(*tert*-butyl)-2*H*-indazole also worked giving the desired products in 81% and 36% yield respectively (5s and 5t). It was a pity that iodination of 2*H*-indazoles in EtOH with *N*-iodo-succinimide (NIS) was not succeeded.

**Table 3:** Substrate scope for mono-chloramination of 2*H*-indazoles^a^^,^^b^

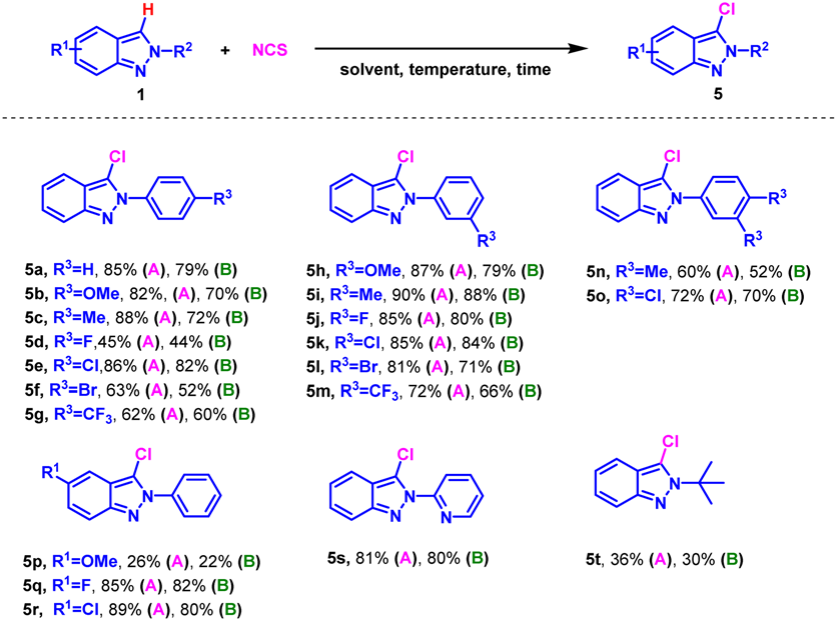

a Reaction conditions: (A) 1 (0.3 mmol), NCS (0.3 mmol), EtOH (3.0 mL), 50 °C, air, 2.0 h. (B) 1 (0.3 mmol), NCS (0.39 mmol), H_2_O (3.0 mL), 95 °C, air, 5.0 h.

b Isolated yield.

We then turned our attention to poly-halogenation of 2*H*-indazoles (Table 4), affording the corresponding di-halogenated products in 64–70% yields (3a–3d). Based on this, the conversion of 2*H*-indazoles to hetero-halogenated indazoles was realized by ‘one-pot, two step’ method. 3-Bromo-7-chloro-2*H*-indazoles were prepared by bromination followed by chlorination with moderate yield (3e and 3f). And 3-chloro-7-bromo-2*H*-indazoles were produced in 65–74% yield through chlorination-bromination process (3g–3j). It was found that the yield of 3-bromo-7-chloro-2*H*-indazoles were higher than 3-chloro-7-bromo-2*H*-indazoles (3e*vs.*3h, 3f*vs.*3i), which might be due to the low reactivity of indazole C7 position and stronger activity of NBS than NCS. By increasing the amount of NBS and prolonging the reaction time, the tribrominated product 4b was obtained in 72% yield from 2-(*m*-tolyl)-2*H*-indazole.

**Table 4:** Substrate scope for poly-halogenation of 2*H*-indazoles^a^^,^^b^

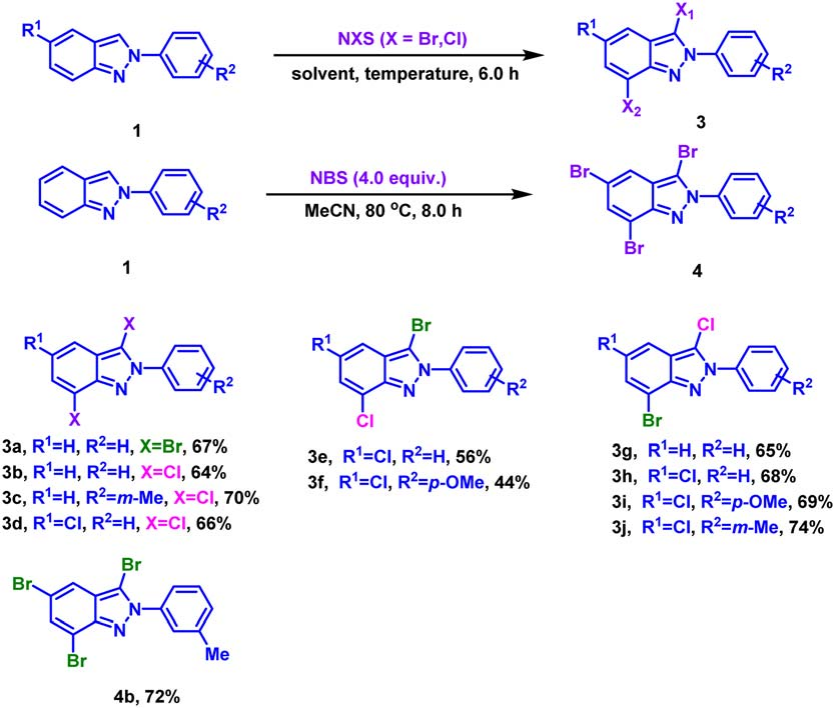

a Reaction conditions: (3a–3d) 1 (0.3 mmol), NXS in batches (0.6 mmol), EtOH (3.0 mL), 50 °C, air, 6.0 h. (3e–3f) step 1: 1 (0.3 mmol), NBS (0.3 mmol), EtOH (3.0 mL), 50 °C, air, 2.0 h; step 2: NCS (0.3 mmol), 50 °C, air, 4.0 h. (3g–3j) step 1: 1 (0.3 mmol), NCS (0.3 mmol), EtOH (3.0 mL), 50 °C, air, 2.0 h; step 2: NBS (0.3 mmol), 50 °C, air, 4.0 h.

b Isolated yield. (4b) MeCN (5.0 mL), 50 °C, air, 8.0 h.

To identify the structures, we took product 3c and 3j as examples to measure DEPT135, ^1^H–^1^H COSY, ^1^H–^13^C HSQC and ^1^H–^13^C HMBC spectra (Fig. 2), the details are listed in the ESI.†

**Fig. 2:**
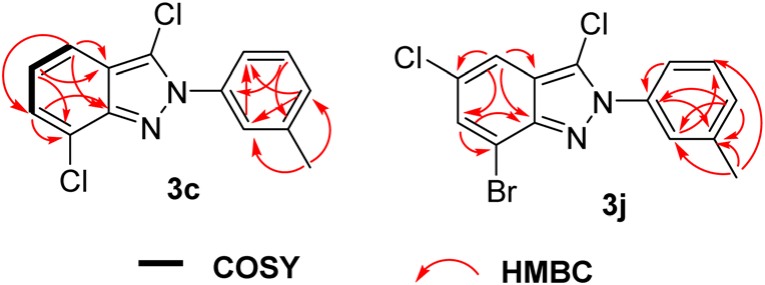
Key COSY and HMBC correlations of compounds 3c and 3j.

For purpose of demonstrating the suitability of this halogenation method on a large scale, a gram-scale reaction was investigated. The results showed that 6.0 mmol of 1a (1.164 g) could be cleanly converted to 2a with either EtOH or H_2_O as solvent (Scheme 1).

**Scheme 1:**
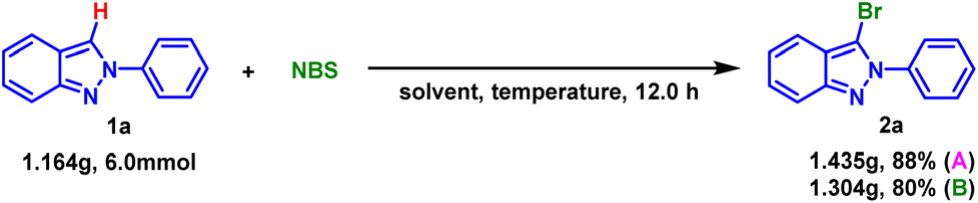
Gram-scale reaction.

In order to gain more insights into the mechanism, a series of control experiments were conducted. Firstly, using isolated mono-brominated product 2a as substrate and 1.0 equiv. NBS as brominating reagent, TLC monitoring showed that the dibrominated product 3a was generated, indicating that dihalogenation occurred after mono-halogenation (Scheme 2a). Second, when 3.0 equiv. 2,2,6,6-tetramethyl-piperidine-1-oxyl (TEMPO) or 2,6-di-*tert*-butyl-4-methylphenol (BHT) was respectively added under the standard reaction conditions, no desired products were formed (Scheme 2b). In addition, bromine radical was captured and 6 was detected by HRMS when ethene-1,1-diyldibenzene was used (Scheme 2c).

**Scheme 2:**
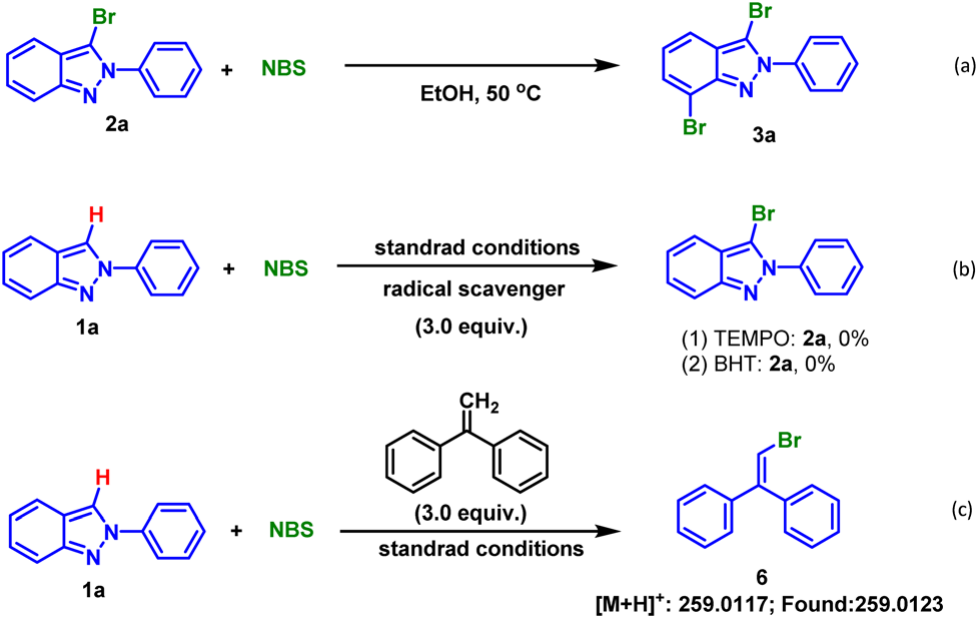
Control experiments.

We considered that a radical pathway mechanism could be involved on the grounds of experimental results and previous reports.^13^ At first, NBS was pyrolyzed under heating conditions to generate bromine radical and radical A. Then substrate 1a reacted with bromine radical to generate intermediate I, which would further oxidize by radical A to produce cationic intermediate II and succinimide anionic B. The proton transfer occurred between the above two ions, and finally succinimide C and mono-brominated product 2a were generated. Similarly, dibrominated product 3a could be obtained from 2a*via* the above pathway (Scheme 3).

**Scheme 3:**
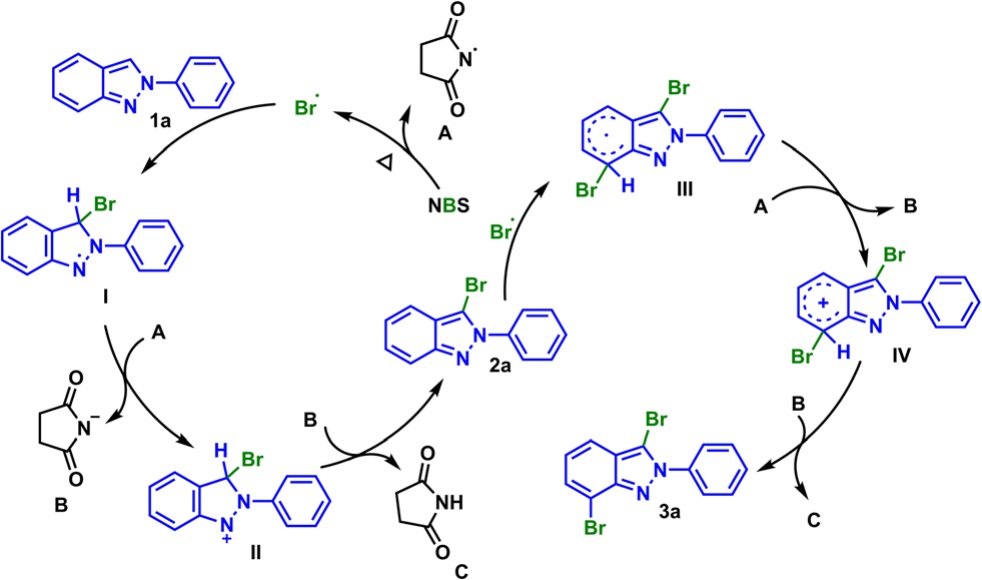
Proposed reaction mechanisms.

## Conclusions

In summary, we have successfully developed a simple and universal metal-free method for the synthesis of mono- and poly-halogenated 2*H*-indazoles. The mono-halogenation could be carried out in water giving products with good yields. Furthermore, hetero-halogenated 2*H*-indazole compounds were also achieved *via* a one-pot reaction. In addition, the gram-scale reaction also produced excellent yields. This new transformation exhibits high selectivity, good functional group tolerance, easy handing and eco-friendliness, rendering the “green” methodology as potential applications in agrochemical and pharmaceutical industries.

## Conflicts of interest

There are no conflicts to declare.

## Supplementary Material

RA-013-D2RA07398F-s001
