# Supplementary material for: Metal-free regioselective mono- and poly-halogenation of 2-substituted indazoles
Source: RSC Adv. 2023 Feb 7;13(8):4958–62. doi: 10.1039/d2ra07398f (PMC9904359; doi:10.1039/d2ra07398f)
Supplement: RA-013-D2RA07398F-s001 [file RA-013-D2RA07398F-s001.pdf]

Electronic Supplementary Material (ESI) for RSC Advances.
This journal is © The Royal Society of Chemistry 2023

Metal-free regioselective mono- and poly-halogenation of 2-substituted indazoles

Changjun Zhang,[a#] Tingting Wei,[a#] Zhichen Yu,[a] Yuxin, Ding,[a] Weike Su,[b] and Yuanyuan Xie[a,b,c]*

[#] Contribute equally

[a] College of Pharmaceutical Science, Zhejiang University of Technology, Hangzhou, 310014, China.

[b] Collaborative Innovation Center of Yangtze River Delta Region Green Pharmaeuticals, Zhejiang University of Technology, Hangzhou,310014, China.

[c] Key Laboratory of Pharmaceutical Engineering of Zhejiang Province, Hangzhou, 310014, China.

E-mail: xyycz@zjut.edu.cn; Fax: (+86) 0571-88320878.

3-bromo-2-phenyl-2*H*-indazole (2a)

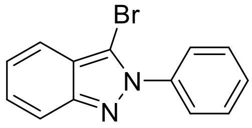

White solid, m.p. 72.4-72.9 ºC, 98% yield, ^{1}H NMR (400 MHz, $CDCl_3$) δ 7.75 (d, J = 8.8 Hz, 1H), 7.68 (d, J = 7.3 Hz, 2H), 7.63 – 7.50 (m, 6H), 7.38 (t, 1H), 7.19 (t, 1H). ^{13}C NMR (100 MHz, $CDCl_3$) δ 149.2, 139.2, 129.4, 129.2, 127.7, 126.3, 123.1, 122.9, 119.8, 118.2, 106.3. HRMS (ESI) calculated for $C_{13}H_{10}BrN_2$ $[M+H]^+$ 273.0022, found 273.0026.

3-bromo-2-(4-methoxyphenyl)-2*H*-indazole (2b)

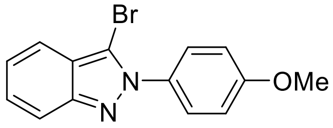

White solid, m.p. 125.4-126.7 ºC, 99% yield, ^{1}H NMR (400 MHz, $CDCl_3$) δ 7.74 (d, J = 8.8 Hz, 1H), 7.58 (d, J = 8.9 Hz, 3H), 7.40 – 7.32 (m, 1H), 7.20 – 7.13 (m, 1H), 7.08 – 7.00 (m, 2H), 3.89 (s, 3H). ^{13}C NMR (100 MHz, $CDCl_3$) δ 160.2, 149.1, 132.3, 127.6, 127.5, 122.9, 122.8, 119.7, 118.2, 114.3, 106.6, 55.7. HRMS (ESI) calculated for $C_{14}H_{12}BrN_2O$ $[M+H]^+$ 303.0128, found 303.0132.

3-bromo-2-(p-tolyl)-2*H*-indazole (2c)

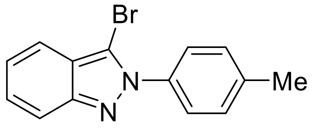

White solid, m.p. 97.5-98.3 ºC, 95% yield, ^{1}H NMR (400 MHz, $CDCl_3$) δ 7.75 (d, J = 8.7 Hz, 1H), 7.61 – 7.52 (m, 3H), 7.36 (t, J = 8.7 Hz, 3H), 7.23 – 7.13 (m, 1H), 2.46 (s, 3H). ^{13}C NMR (100 MHz, $CDCl_3$) δ 149.15, 139.50, 136.87, 129.73, 127.60, 126.03, 122.98, 122.90, 119.76, 118.23, 106.34, 21.40. HRMS (ESI) calculated for $C_{14}H_{12}BrN_2$ $[M+H]^+$ 287.0178, found 287.0182.

3-bromo-2-(4-fluorophenyl)-2*H*-indazole (2d)

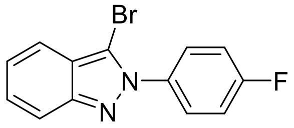

White solid, m.p. 105.0-105.7 ºC, 96% yield, ^{1}H NMR (400 MHz, $CDCl_3$) δ 7.73 (d, J = 8.8 Hz, 1H), 7.70 – 7.61 (m, 2H), 7.58 (d, J = 8.5 Hz, 1H), 7.43 – 7.33 (m, 1H), 7.27 – 7.21 (m, 2H), 7.21 – 7.15 (m, 1H). ^{13}C NMR (100 MHz, $CDCl_3$) δ 162.8 (d, ^{1}J = 248.4 Hz), 149.3, 135.4 (d, ^{4}J = 3.1 Hz), 128.2 (d, ^{3}J = 9.0 Hz), 127.9, 123.3, 122.9, 119.8, 118.2, 116.2 (d, ^{2}J = 23.0 Hz), 106.5. ^{19}F NMR (376 MHz, $CDCl_3$) δ -111.19. HRMS (ESI) calculated for $C_{13}H_9BrFN_2$ $[M+H]^+$ 290.9928, found 290.9932.

3-bromo-2-(4-chlorophenyl)-2*H*-indazole (2e)

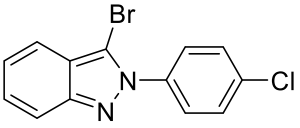

White solid, m.p. 146.4-147.7 °C, 96% yield, ^{1}H NMR (400 MHz, $CDCl_3$) δ 7.73 (d, *J* = 8.8 Hz, 1H), 7.64 (d, *J* = 8.7 Hz, 2H), 7.57 (d, *J* = 8.5 Hz, 1H), 7.53 (d, *J* = 8.7 Hz, 2H), 7.40 – 7.34 (m, 1H), 7.22 – 7.15 (m, 1H). ^{13}C NMR (100 MHz, $CDCl_3$) *δ* 149.4, 137.8, 135.3, 129.4, 128.0, 127.5, 123.3, 123.1, 120.0, 118.3, 106.2. HRMS (ESI) calculated for $C_{13}H_9BrClN_2$ $[M+H]^+$ 306.9632, found 306.9636.

3-bromo-2-(4-(trifluoromethyl)phenyl)-2*H*-indazole (2f)

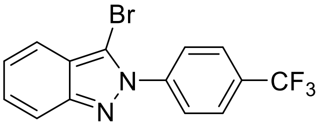

White solid, m.p. 111.7-112.4 °C, 96% yield, ^{1}H NMR (400 MHz, $CDCl_3$) δ 7.85 (q, *J* = 8.7 Hz, 4H), 7.74 (d, *J* = 8.8 Hz, 1H), 7.59 (d, *J* = 8.5 Hz, 1H), 7.42 – 7.36 (m, 1H), 7.24 – 7.17 (m, 1H). ^{13}C NMR (100 MHz, $CDCl_3$) *δ* 149.6, 142.0, 131.2 (q, ^{2}J = 32.8 Hz), 128.3, 126.5, 126.4 (q, ^{3}J = 3.7 Hz), 123.7 (q, ^{1}J = 270.8 Hz), 123.6, 123.3, 119.9, 118.3, 106.2. ^{19}F NMR (376 MHz, $CDCl_3$) *δ* -62.53. HRMS (ESI+) calculated for $C_{14}H_9BrF_3N_2$ $[M+H]^+$ 340.9896, found 340.9900.

3-bromo-2-(3-methoxyphenyl)-2*H*-indazole (2g)

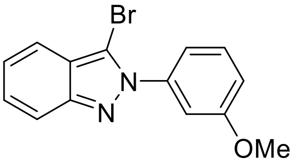

White solid, m.p. 87.1-89.2 °C, 87% yield, ^{1}H NMR (400 MHz, $CDCl_3$) δ 7.75 (d, *J* = 8.8 Hz, 1H), 7.59 (d, *J* = 8.5 Hz, 1H), 7.46 (t, *J* = 8.1 Hz, 1H), 7.39 – 7.34 (m, 1H), 7.28 – 7.25 (m, 1H), 7.22 (t, *J* = 2.2 Hz, 1H), 7.21 – 7.16 (m, 1H), 7.09 – 7.03 (m, 1H), 3.88 (s, 3H). ^{13}C NMR (100 MHz, $CDCl_3$) *δ* 160.1, 149.2, 140.2, 129.8, 127.8, 123.1, 123.0, 119.8, 118.50, 118.2, 115.6, 111.8, 106.4, 55.7. HRMS (ESI) calculated for $C_{14}H_{12}BrN_2O$ $[M+H]^+$ 303.0128, found 303.0130.

3-bromo-2-(m-tolyl)-2*H*-indazole (2h)

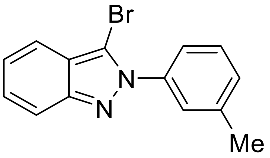

White solid, m.p. 55.6-56.8 ºC, 93% yield, ^{1}H NMR (400 MHz, $CDCl_3$) δ 7.75 (d, *J* = 8.8 Hz, 1H), 7.59 (d, *J* = 8.5 Hz, 1H), 7.51 – 7.31 (m, 5H), 7.22 – 7.15 (m, 1H), 2.47 (s, 3H). ^{13}C NMR (100 MHz, $CDCl_3$) *δ* 149.1, 139.4, 139.1, 130.1, 128.9, 127.7, 126.8, 123.3, 123.0, 122.9, 119.8, 118.2, 106.3, 21.5. HRMS (ESI) calculated for $C_{14}H_{12}BrN_2$ $[M+H]^+$ 287.0178, found 287.0182

3-bromo-2-(3-fluorophenyl)-2*H*-indazole (2i)

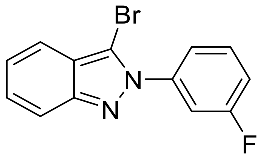

White solid, m.p. 92.2-93.5 ºC, 95% yield, ^{1}H NMR (400 MHz, $CDCl_3$) δ 7.73 (d, *J* = 8.8 Hz, 1H), 7.58 (d, *J* = 8.5 Hz, 1H), 7.56 – 7.50 (m, 2H), 7.50 – 7.43 (m, 1H), 7.41 – 7.34 (m, 1H), 7.26 – 7.14 (m, 2H). ^{13}C NMR (100 MHz, $CDCl_3$) *δ* 162.5 (d, ^1J = 247.0 Hz), 149.4, 140.5 (d, ^3J = 10.0 Hz), 130.4 (d, ^3J = 8.9 Hz), 128.1, 123.4, 123.2, 122.0 (d, ^4J = 3.3 Hz), 119.8, 118.3, 116.4 (d, ^2J = 20.9 Hz), 114.0 (d, ^2J = 24.9 Hz), 106.2. ^{19}F NMR (376 MHz, $CDCl_3$) *δ* -110.61. HRMS (ESI) calculated for $C_{13}H_9BrFN_2$ $[M+H]^+$ 290.9928, found 290.9932.

3-bromo-2-(3-chlorophenyl)-2*H*-indazole (2j)

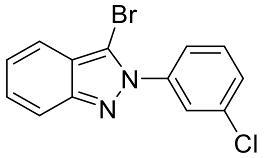

White solid, m.p. 105.0-105.4 ºC, 91% yield, ^{1}H NMR (400 MHz, $CDCl_3$) δ 7.78 – 7.70 (m, 2H), 7.64 – 7.56 (m, 2H), 7.53 – 7.46 (m, 2H), 7.40 – 7.35 (m, 1H), 7.22 – 7.14 (m, 1H). ^{13}C NMR (101 MHz, $CDCl_3$) *δ* 149.4, 140.2, 134.9, 130.2, 129.5, 128.1, 126.6, 124.4, 123.4, 123.2, 119.8, 118.3, 106.3. HRMS (ESI) calculated for $C_{13}H_9BrClN_2$ $[M+H]^+$ 306.9632, found 306.9633.

3-bromo-2-(3-bromophenyl)-2*H*-indazole (2k)

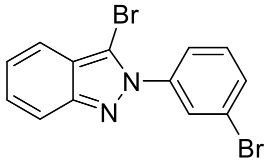

White solid, m.p. 109.7-110.6 ºC, 88% yield, ^{1}H NMR (400 MHz, $CDCl_3$) δ 7.89 (t, *J* = 1.9 Hz, 1H), 7.73 (d, *J* = 8.8 Hz, 1H), 7.68 – 7.62 (m, 2H), 7.57 (td, *J* = 8.5 Hz, 1H), 7.42 (t, *J* = 8.1 Hz, 1H), 7.40 – 7.34 (m, 1H), 7.22 – 7.16 (m, 1H). ^{13}C NMR (100 MHz, $CDCl_3$) *δ* 149.4, 140.3, 132.4, 130.4, 129.4, 128.1, 124.9, 123.4, 123.1, 122.6, 119.8, 118.3, 106.3. HRMS (ESI): calculated for $C_{13}H_9Br_2N_2$ $[M+H]^+$ 350.9127, found 350.9131.

3-bromo-2-(3-(trifluoromethyl)phenyl)-2*H*-indazole (2l)

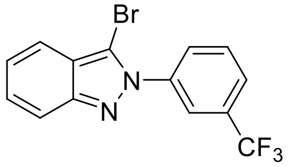

Yellow oil, 82% yield, ^{1}H NMR (400 MHz, CDCl$_3$) δ 8.03 (s, 1H), 7.92 (d, *J* = 8.0 Hz, 1H), 7.81 – 7.64 (m, 3H), 7.58 (d, *J* = 8.5 Hz, 1H), 7.42 – 7.34 (m, 1H), 7.23 – 7.14 (m, 1H). ^{13}C NMR (100 MHz, CDCl$_3$) *δ* 149.6, 139.7, 131.9 (q, ^{2}J = 33.1 Hz), 129.8, 129.4, 128.2, 126.0 (q, ^{3}J = 3.6 Hz), 123.5, 123.5 (q, ^{1}J = 270.9 Hz), 123.4 (q, ^{3}J = 3.9 Hz) 123.3, 119.8, 118.3, 106.3. ^{19}F NMR (376 MHz, CDCl$_3$) *δ* -62.66. HRMS (ESI) calculated for $C_{14}H_9BrF_3N_2$ [M+H]$^+$ 340.9896, found 340.9900.

3-bromo-2-(3,4-dimethylphenyl)-2*H*-indazole (2m)

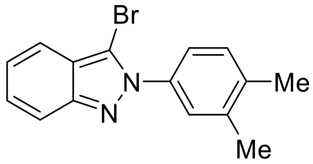

Yellow oil, 75% yield, ^{1}H NMR (400 MHz, CDCl$_3$) δ 7.76 (d, *J* = 8.8 Hz, 1H), 7.58 (d, *J* = 8.4 Hz, 1H), 7.46 (s, 1H), 7.42 – 7.33 (m, 2H), 7.29 (d, *J* = 8.0 Hz, 1H), 7.17 (t, 1H), 2.36 (s, 6H). ^{13}C NMR (100 MHz, CDCl$_3$) *δ* 149.1, 138.1, 137.8, 137.0, 130.0, 127.5, 127.1, 123.4, 122.9, 122.8, 119.7, 118.2, 106.2, 19.9, 19.7. HRMS (ESI) calculated for $C_{15}H_{14}BrN_2$ [M+H]$^+$ 301.0335, found 301.0338.

3-bromo-2-(3,4-dichlorophenyl)-2*H*-indazole (2n)

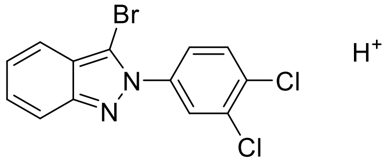

White solid, m.p. 140.8-142.4 ºC, 83% yield, ^{1}H NMR (400 MHz, CDCl$_3$) δ 7.86 (d, *J* = 2.3 Hz, 1H), 7.71 (d, *J* = 8.8 Hz, 1H), 7.65 – 7.54 (m, 3H), 7.40 – 7.34 (m, 1H), 7.22 – 7.14 (m, 1H). ^{13}C NMR (100 MHz, CDCl$_3$) *δ* 149.6, 138.4, 133.7, 133.3, 130.8, 128.3, 128.1, 125.3, 123.6, 123.3, 119.8, 118.3, 106.2. HRMS (ESI) calculated for $C_{13}H_8BrCl_2N_2$ [M+H]$^+$ 340.9242, found 340.9242.

3-bromo-5-methoxy-2-phenyl-2*H*-indazole (2o)

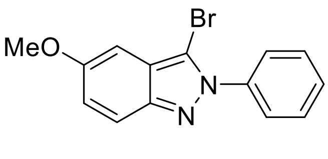

White solid, m.p. 139.0-150.5 ºC, 31% yield, ^{1}H NMR (400 MHz, CDCl$_3$) *δ* 7.73 – 7.60 (m, 3H), 7.59 – 7.45 (m, 3H), 7.06 (d, *J* = 9.2 Hz, 1H), 6.73 (s, 1H), 3.89 (s, 3H). ^{13}C NMR (100 MHz, CDCl$_3$) *δ* 156.2,

146.1, 139.4, 129.1, 126.1, 123.1, 123.0, 119.7, 104.6, 95.4, 55.6. HRMS (ESI) calculated for $C_{14}H_{12}BrN_2O$ $[M+H]^+$ 303.0128, found 303.0129.

3-bromo-5-fluoro-2-phenyl-2*H*-indazole (2p)

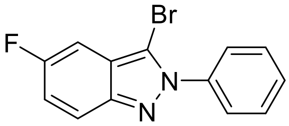

White solid, m.p. 123.1-123.5 °C, 91% yield, ^1H NMR (400 MHz, $CDCl_3$) δ 7.75 – 7.70 (m, 1H), 7.69 – 7.64 (m, 2H), 7.60 – 7.51 (m, 3H), 7.21 – 7.12 (m, 2H). ^{13}C NMR (100 MHz, $CDCl_3$) δ 159.1 (d, ^1J = 241.4 Hz), 146.6, 139.2, 129.5, 129.2, 126.2, 122.5 (d, ^3J = 11.9 Hz), 120.6 (d, ^3J = 9.6 Hz), 119.6 (d, ^2J = 28.9 Hz), 106.0 (d, ^4J = 8.9 Hz), 102.4 (d, ^2J = 25.1 Hz). ^{19}F NMR (376 MHz, $CDCl_3$) *δ* -117.57. HRMS (ESI) calculated for $C_{13}H_9BrFN_2$ $[M+H]^+$ 290.9928, found 290.9930.

3-bromo-5-chloro-2-phenyl-2H-indazole (2q)

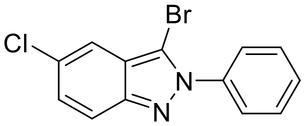

Yellow solid, m.p. 140.2-140.9 °C, 98% yield, ^1H NMR (400 MHz, Chloroform-*d*) δ 7.69 – 7.63 (m, 3H), 7.58 – 7.48 (m, 4H), 7.30 – 7.26 (m, 1H). ^{13}C NMR (150 MHz, $CDCl_3$) δ 147.60, 139.07, 129.55, 129.22, 129.18, 128.81, 126.13, 123.41, 119.91, 118.51, 105.77. HRMS (ESI+) calculated for $C_{13}H_9BrClN_2$ $[M+H]^+$ 306.9632, found 306.9632.

3-bromo-2-(pyridin-2-yl)-2*H*-indazole (2r)

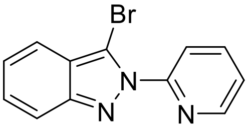

Orange oil, 81% yield, ^1H NMR (400 MHz, $CDCl_3$) *δ* 8.63 (d, *J* = 4.1 Hz, 1H), 7.94 – 7.82 (m, 2H), 7.72 (d, *J* = 8.8 Hz, 1H), 7.58 (d, *J* = 8.5 Hz, 1H), 7.43 – 7.29 (m, 2H), 7.18 – 7.10 (m, 1H). ^{13}C NMR (100 MHz, $CDCl_3$) *δ* 152.0, 149.4, 148.5, 138.6, 128.2, 123.9, 123.6, 123.3, 120.1, 119.8, 118.4, 105.5. HRMS (ESI+) calculated for $C_{12}H_9BrN_3$ $[M+H]^+$ 273.9974, found 273.9977.

3-bromo-2-(tert-butyl)-2*H*-indazole (2s)

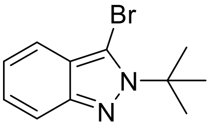

Yellow oil, 36% yield, ^{1}H NMR (400 MHz, $CDCl_3$) δ 7.68 (d, *J* = 8.7 Hz, 1H), 7.52 (d, *J* = 8.5 Hz, 1H), 7.31 – 7.27 (m, 1H), 7.14 – 7.08 (m, 1H), 1.90 (s, 9H). ^{13}C NMR (100 MHz, $CDCl_3$) δ 146.5, 126.6, 124.0, 122.4, 119.3, 118.1, 102.6, 63.6, 29.9. HRMS (ESI) calculated for $C_{11}H_{14}BrN_2$ $[M+H]^+$ 253.0335, found 253.0338.

3-chloro-2-phenyl-2*H*-indazole (5a)

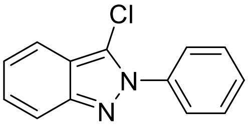

Yellow solid, m.p. 39.0-40.8 ºC,85% yield, ^{1}H NMR (400 MHz, $CDCl_3$) δ 7.77 – 7.68 (m, 3H), 7.63 (d, *J* = 8.5 Hz, 1H), 7.59 – 7.49 (m, 3H), 7.36 (t, 1H), 7.17 (t, 1H). ^{13}C NMR (100 MHz, $CDCl_3$) δ 148.6, 138.6, 129.3, 127.7, 125.8, 122.9, 121.2, 120.0, 119.6, 119.1, 118.3. HRMS (ESI) calculated for $C_{13}H_{10}ClN_2$ $[M+H]^+$ 229.0527, found 229.0531.

3-chloro-2-(4-methoxyphenyl)-2*H*-indazole (5b)

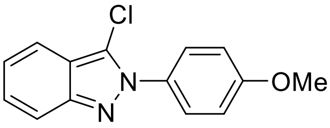

White solid, m.p. 89.5-91.1 ºC, 82% yield, ^{1}H NMR (400 MHz, $CDCl_3$) *δ* 7.71 (d, *J* = 8.8 Hz, 1H), 7.60 (t, *J* = 7.4 Hz, 3H), 7.35 (t, *J* = 7.7 Hz, 1H), 7.15 (t, *J* = 7.5 Hz, 1H), 7.05 (d, *J* = 8.8 Hz, 2H), 3.88 (s, 3H). ^{13}C NMR (100 MHz, $CDCl_3$) *δ* 160.1, 148.4, 131.5, 127.5, 127.1, 122.7, 119.8, 119.9, 119.0, 118.2, 114.3, 55.7. HRMS (ESI) calculated for $C_{14}H_{12}ClN_2O$ $[M+H]^+$ 259.0633, found 259.0635.

3-chloro-2-(p-tolyl)-2*H*-indazole (5c)

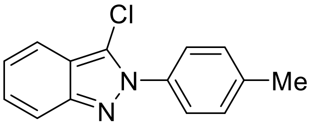

White solid, m.p. 87.2-88.3 ºC, 88% yield, ^{1}H NMR (400 MHz, $CDCl_3$) δ 7.72 (d, *J* = 8.8 Hz, 1H), 7.62 (d, *J* = 8.5 Hz, 1H), 7.60 – 7.54 (m, 2H), 7.39 – 7.32 (m, 3H), 7.19 – 7.13 (m, 1H), 2.46 (s, 3H). ^{13}C NMR (100 MHz, $CDCl_3$) *δ* 148.52, 139.43, 136.13, 129.82, 127.60, 125.61, 122.79, 119.93, 119.64, 119.09, 118.26, 21.40. HRMS (ESI) calculated for $C_{14}H_{12}ClN_2$ $[M+H]^+$ 243.0684, found 243.0688.

3-chloro-2-(4-fluorophenyl)-2*H*-indazole (5d)

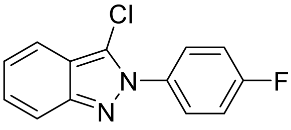

White solid, m.p. 103.6-105.1 ºC, 45% yield, ^{1}H NMR (400 MHz, $CDCl_3$) δ 7.73 – 7.63 (m, 3H), 7.61 (d, *J* = 8.5 Hz, 1H), 7.39 – 7.31 (m, 1H), 7.28 – 7.20 (m, 2H), 7.19 – 7.13 (m, 1H). ^{13}C NMR (100 MHz, $CDCl_3$) *δ* 162.8 (d, ^{1}J = 248.4 Hz), 148.6, 134.6 (d, ^{4}J = 3.1 Hz), 127.8, 127.7 (d, ^{3}J = 8.8 Hz), 123.0, 119.9, 119.7, 119.0, 118.2, 116.2 (d, ^{2}J = 23.0 Hz). ^{19}F NMR (376 MHz, $CDCl_3$) *δ* -111.37. HRMS (ESI) calculated for $C_{13}H_9ClFN_2$ $[M+H]^+$ 247.0433, found 247.0435.

3-chloro-2-(4-chlorophenyl)-2*H*-indazole (5e)

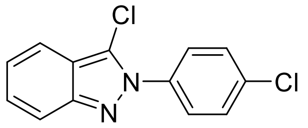

White solid, m.p. 138.7-140.0 ºC, 86% yield, ^{1}H NMR (400 MHz, $CDCl_3$) δ 7.71 (d, *J* = 8.8 Hz, 1H), 7.69 – 7.64 (m, 2H), 7.61 (d, *J* = 8.5 Hz, 1H), 7.56 – 7.49 (m, 2H), 7.39 – 7.33 (m, 1H), 7.20 – 7.14 (m, 1H). ^{13}C NMR (100 MHz, $CDCl_3$) *δ* 148.8, 137.1, 135.2, 129.5, 128.0, 127.0, 123.1, 120.1, 119.6, 119.1, 118.3. HRMS (ESI) calculated for $C_{13}H_9Cl_2N_2$ $[M+H]^+$ 263.0137, found 263.0140.

3-chloro-2-(4-chlorophenyl)-2*H*-indazole (5f)

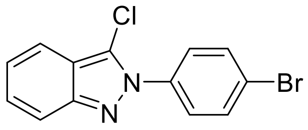

White solid, m.p. 144.3-146.5 ºC, 63% yield, ^{1}H NMR (400 MHz, $CDCl_3$) δ 7.76 – 7.63 (m, 3H), 7.63 – 7.52 (m, 3H), 7.39 – 7.32 (m, 1H), 7.20 – 7.13 (m, 1H). ^{13}C NMR (100 MHz, $CDCl_3$) *δ* 148.8, 137.6, 132.5, 128.0, 127.2, 123.3, 123.1, 119.5, 119.1, 118.3. HRMS (ESI) calculated for $C_{13}H_9BrClN_2$ $[M+H]^+$ 306.9632, found 306.9636.

3-chloro-2-(4-(trifluoromethyl)phenyl)-2*H*-indazole (5g)

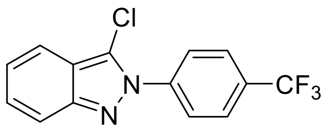

Yellow oil, 62% yield, ^{1}H NMR (400 MHz, $CDCl_3$) *δ* 7.90 (d, *J* = 8.5 Hz, 2H), 7.83 (d, *J* = 8.6 Hz, 2H), 7.72 (d, *J* = 8.8 Hz, 1H), 7.62 (d, *J* = 8.6 Hz, 1H), 7.42 – 7.33 (m, 1H), 7.21 – 7.13 (m, 1H). ^{13}C NMR (100 MHz, $CDCl_3$) *δ* 149.0, 141.4, 131.1 (q, ^{2}J = 32.8 Hz), 128.2, 126.5 (q, ^{3}J = 3.7 Hz), 126.0, 123.8 (q, ^{1}J = 270.8 Hz), 123.4, 120.3, 119.7, 119.1, 118.4. ^{19}F NMR (376 MHz, $CDCl_3$) *δ* -62.60. HRMS (ESI+) calculated for $C_{14}H_9ClF_3N_2$ $[M+H]^+$ 297.0401, found 297.0405.

3-chloro-2-(3-methoxyphenyl)-2*H*-indazole (5h)

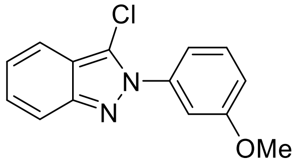

Yellow oil, 87% yield, ^{1}H NMR (400 MHz, $CDCl_3$) δ 7.73 (d, *J* = 8.8 Hz, 1H), 7.62 (d, *J* = 8.5 Hz, 1H), 7.44 (t, *J* = 8.1 Hz, 1H), 7.39 – 7.33 (m, 1H), 7.29 (d, *J* = 8.6 Hz, 1H), 7.26 – 7.23 (m, 1H), 7.16 (t, 1H), 7.05 (dd, *J* = 8.3, 2.3 Hz, 1H), 3.87 (s, 3H). ^{13}C NMR (100 MHz, $CDCl_3$) *δ* 160.1, 148.5, 139.5, 129.9, 127.7, 122.9, 120.0, 119.6, 119.1, 118.2, 118.0, 115.4, 111.3, 55.7. HRMS (ESI) calculated for $C_{14}H_{12}ClN_2O$ $[M+H]^+$ 259.0633, found 259.0635.

3-chloro-2-(3-methoxyphenyl)-2*H*-indazole (5i)

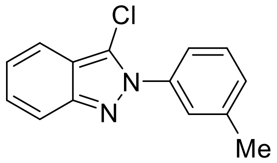

Yellow oil, 90% yield, ^{1}H NMR (400 MHz, $CDCl_3$) *δ* 7.73 (d, *J* = 8.8 Hz, 1H), 7.63 (d, *J* = 8.5 Hz, 1H), 7.52 (s, 1H), 7.50 (d, *J* = 8.2 Hz, 1H), 7.43 (t, *J* = 7.7 Hz, 1H), 7.39 – 7.33 (m, 1H), 7.31 (d, *J* = 7.5 Hz, 1H), 7.19 – 7.13 (m, 1H), 2.46 (s, 3H). ^{13}C NMR (100 MHz, $CDCl_3$) *δ* 148.5, 139.4, 138.4, 130.0, 128.9, 127.6, 126.4, 122.8, 122.7, 119.9, 119.5, 119.0, 118.2, 21.4. HRMS (ESI) calculated for $C_{14}H_{12}ClN_2$ $[M+H]^+$ 243.0684, found 243.0686.

3-chloro-2-(3-fluorophenyl)-2*H*-indazole (5j)

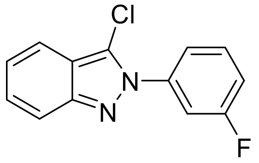

Yellow solid, m.p. 69.8-72.0 °C, 85% yield, ^{1}H NMR (400 MHz, $CDCl_3$) δ 7.71 (d, *J* = 8.8 Hz, 1H), 7.62 (d, *J* = 8.5 Hz, 1H), 7.58 – 7.47 (m, 3H), 7.40 – 7.33 (m, 1H), 7.25 – 7.12 (m, 2H). ^{13}C NMR (100 MHz, $CDCl_3$) *δ* 162.3 (d, ^{1}J = 246.9 Hz), 148.8, 139.7 (d, ^{3}J = 10.0 Hz), 130.5 (d, ^{3}J = 8.9 Hz), 128.0, 123.2, 121.4 (d, ^{4}J = 3.3 Hz), 120.1, 119.6, 119.1, 118.3, 116.2 (d, ^{2}J = 20.9 Hz), 113.4 (d, ^{2}J = 25.0 Hz). ^{19}F NMR (376 MHz, $CDCl_3$) *δ* -110.55. HRMS (ESI) calculated for $C_{13}H_9ClFN_2$ $[M+H]^+$ 247.0433, found 247.0437.

3-chloro-2-(3-chlorophenyl)-2*H*-indazole (5k)

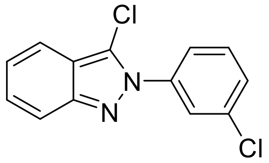

White solid, m.p. 67.7-70.0 ºC, 85% yield, ^{1}H NMR (400 MHz, $CDCl_3$) δ 7.76 (s, 1H), 7.71 (d, J = 8.8 Hz, 1H), 7.65 – 7.58 (m, 2H), 7.48 (d, J = 5.0 Hz, 2H), 7.40 – 7.32 (m, 1H), 7.20 – 7.12 (m, 1H). ^{13}C NMR (100 MHz, $CDCl_3$) δ 148.78, 139.5, 134.9, 130.2, 129.3, 128.0, 126.2, 123.8, 123.2, 120.1, 119.6, 119.1, 118.3. HRMS (ESI) calculated for $C_{13}H_9Cl_2N_2$ [M+H]$^+$ 263.0137, found 263.0140.

2-(3-bromophenyl)-3-chloro-2*H*-indazole (5l)

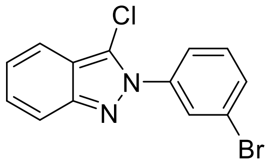

Yellow solid, m.p. 92.7-94.3 ºC, 81% yield, ^{1}H NMR (400 MHz, $CDCl_3$) δ 7.91 (t, J = 1.9 Hz, 1H), 7.73 – 7.55 (m, 4H), 7.41 (t, J = 8.1 Hz, 1H), 7.38 – 7.32 (m, 1H), 7.19 – 7.12 (m, 1H). ^{13}C NMR (100 MHz, $CDCl_3$) δ 148.8, 139.5, 132.2, 130.4, 128.8, 128.0, 124.2, 123.2, 122.7, 120.1, 119.6, 119.1, 118.3. HRMS (ESI) calculated for $C_{13}H_9BrClN_2$ [M+H]$^+$ 306.9632, found 306.9632.

3-chloro-2-(3-(trifluoromethyl)phenyl)-2*H*-indazole (5m)

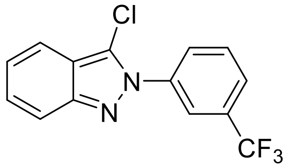

Yellow oil, 72% yield, ^{1}H NMR (400 MHz, $CDCl_3$) δ 8.05 (s, 1H), 7.94 (d, J = 8.0 Hz, 1H), 7.77 (d, J = 7.8 Hz, 1H), 7.74 – 7.66 (m, 2H), 7.62 (d, J = 8.5 Hz, 1H), 7.40 – 7.34 (m, 1H), 7.20 – 7.14 (m, 1H). ^{13}C NMR (100 MHz, $CDCl_3$) δ 149.0, 139.0, 132.0 (q, ^2J = 33.1 Hz), 129.9, 128.8, 128.8, 128.2, 125.8 (q, ^3J = 3.7 Hz), 123.5 (q, ^1J = 270.1 Hz), 123.3, 122.8 (q, ^3J = 3.9 Hz), 120.2, 119.1, 118.3. ^{19}F NMR (376 MHz, $CDCl_3$) δ -62.68. HRMS (ESI) calculated for $C_{14}H_9ClF_3N_2$ [M+H]$^+$ 297.0401, found 297.0405.

3-chloro-2-(3,4-dimethylphenyl)-2*H*-indazole (5n)

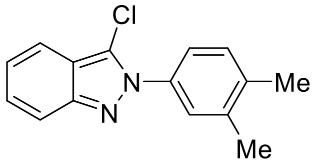

Yellow oil, 60% yield, ^{1}H NMR (400 MHz, $CDCl_3$) δ 7.73 (d, J = 8.8 Hz, 1H), 7.62 (d, J = 8.5 Hz, 1H), 7.47 (s, 1H), 7.41 (d, J = 8.0 Hz, 1H), 7.38 – 7.32 (m, 1H), 7.30 (d, J = 8.0 Hz, 1H), 7.19 – 7.12 (m, 1H), 2.36 (s, 6H). ^{13}C NMR (100 MHz, $CDCl_3$) δ 148.4, 138.1, 137.9, 136.3, 130.1, 127.5, 126.7, 122.9, 122.7, 119.9, 119.5, 119.0, 118.2, 19.95, 19.7. HRMS (ESI) calculated for $C_{15}H_{14}ClN_2$ $[M+H]^+$ 257.0840, found 257.0844.

3-chloro-2-(3,4-dichlorophenyl)-2*H*-indazole (5o)

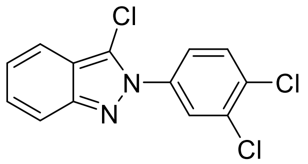

White solid, m.p. 137.4-141.5 ºC, 72% yield, ^{1}H NMR (400 MHz, $CDCl_3$) δ 7.89 (s, 1H), 7.69 (d, J = 8.8 Hz, 1H), 7.65 – 7.56 (m, 3H), 7.36 (t, 1H), 7.21 – 7.13 (m, 1H). ^{13}C NMR (100 MHz, $CDCl_3$) δ 148.9, 137.7, 133.6, 133.4, 130.9, 128.2, 127.6, 124.7, 123.4, 120.2, 119.6, 119.1, 118.3. HRMS (ESI) calculated for $C_{13}H_8Cl_3N_2$ $[M+H]^+$ 296.9748, found 296.9752.

3-chloro-5-methoxy-2-phenyl-2*H*-indazole (5p)

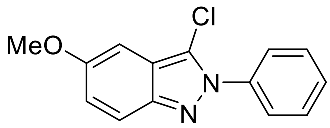

White solid, m.p. 148.4-148.5 ºC, 26% yield, ^{1}H NMR (400 MHz, $CDCl_3$) δ 7.69 (d, J = 7.5 Hz, 2H), 7.61 (d, J = 9.3 Hz, 1H), 7.55 (t, J = 7.6 Hz, 2H), 7.48 (t, J = 7.0 Hz, 1H), 7.06 (dd, J = 9.3, 2.3 Hz, 1H), 6.77 (d, J = 2.1 Hz, 1H), 3.89 (s, 3H). ^{13}C NMR (100 MHz, $CDCl_3$) δ 156.0, 145.5, 138.7, 129.2, 129.0, 125.6, 123.1, 120.0, 119.8, 118.1, 94.7, 55.6. HRMS (ESI) calculated for $C_{14}H_{12}ClN_2O$ $[M+H]^+$ 259.0633, found 259.0633.

3-chloro-5-fluoro-2-phenyl-2*H*-indazole (5q)

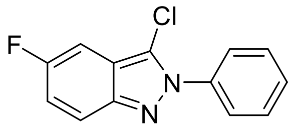

White solid, m.p. 102.6-104.9 ºC,85% yield, ^{1}H NMR (400 MHz, $CDCl_3$) δ 7.74 – 7.66 (m, 3H), 7.59 – 7.50 (m, 3H), 7.23 – 7.09 (m, 2H). ^{13}C NMR (100 MHz, $CDCl_3$) δ 157.9 (d, ^1J = 241.4 Hz), 146.0, 138.5, 129.4, 129.3, 125.7, 120.7 (d, ^3J = 9.6 Hz), 119.6 (d, ^4J = 9.0 Hz), 119.6 (d, ^2J = 29.0 Hz), 119.4 (d, ^3J = 11.9 Hz), 101.6 (d, ^2J = 25.1 Hz). ^{19}F NMR (376 MHz, $CDCl_3$) δ -117.71. HRMS (ESI) calculated for $C_{13}H_9ClFN_2$ $[M+H]^+$ 247.0433, found 247.0437.

3,5-dichloro-2-phenyl-2*H*-indazole (5r)

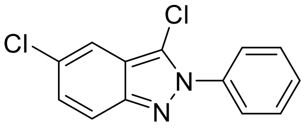

Yellow solid, m.p. 115.8-116.9 °C,89% yield, ^{1}H NMR (400 MHz, $CDCl_3$) δ 7.70 – 7.63 (m, 3H), 7.61 (s, 1H), 7.59 – 7.50 (m, 3H), 7.30 – 7.26 (m, 1H). ^{13}C NMR (150 MHz, $CDCl_3$) δ 146.9, 138.3, 129.5, 129.3, 129.2, 128.7, 125.7, 120.4, 119.9, 119.3, 117.8. HRMS (ESI) calculated for $C_{13}H_9Cl_2N_2$ $[M+H]^+$ 263.0137, found 263.0140.

3-chloro-2-(pyridin-2-yl)-2*H*-indazol (5s)

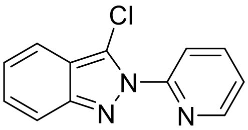

Blown oil, 81% yield, ^{1}H NMR (400 MHz, $CDCl_3$) *δ* 8.65 (d, *J* = 4.2 Hz, 1H), 7.92 (td, *J* = 7.8, 1.7 Hz, 1H), 7.87 (d, *J* = 8.0 Hz, 1H), 7.70 (d, *J* = 8.9 Hz, 1H), 7.63 (d, *J* = 8.5 Hz, 1H), 7.45 – 7.38 (m, 1H), 7.37 – 7.31 (m, 1H), 7.14 (dd, *J* = 8.4, 6.7 Hz, 1H). ^{13}C NMR (100 MHz, $CDCl_3$) *δ* 151.6, 148.8, 148.7, 138.7, 128.2, 123.9, 123.2, 120.6, 119.9, 119.6, 119.4, 118.4. HRMS (ESI) calculated for $C_{12}H_9ClN_3$ $[M+H]^+$ 230.0480, found 230.0484.

2-(*tert*-butyl)-3-chloro-2*H*-indazole (5t)

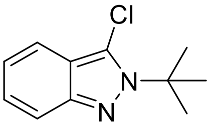

Yellow oil, 36% yield, ^{1}H NMR (400 MHz, DMSO-d_6) *δ* 7.63 (d, *J* = 8.7 Hz, 1H), 7.54 (d, *J* = 8.4 Hz, 1H), 7.32 – 7.25 (m, 1H), 7.15 – 7.08 (m, 1H), 1.77 (s, 9H). ^{13}C NMR (100 MHz, DMSO) *δ* 145.2, 126.4, 122.3, 120.3, 118.2, 118.0, 116.5, 62.8, 29.0. HRMS (ESI) calculated for $C_{11}H_{14}ClN_3$ $[M+H]^+$ 209.0480, found 209.0484.

3,7-dibromo-2-phenyl-2*H*-indazole (3a)

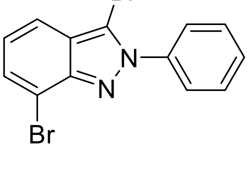

White solid, m.p. 204.3-205.1 °C, 67% yield, ^{1}H NMR (400 MHz, $CDCl_3$) δ 7.68 (d, *J* = 7.3 Hz, 2H), 7.62 – 7.49 (m, 5H), 7.05 (t, *J* = 7.8 Hz, 1H). ^{13}C NMR (100 MHz, $CDCl_3$) δ 147.8, 139.1, 130.4, 129.7,

129.2, 126.6, 123.7, 123.6, 119.4, 111.7, 107.7. HRMS (ESI) calculated for $C_{13}H_9Br_2N_2$ $[M+H]^+$ 350.9127, found 350.9131.

3,7-dichloro-2-phenyl-2*H*-indazole (3b)

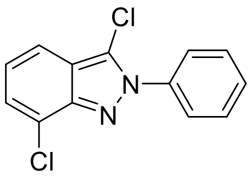

White solid, m.p. 172.3-173.5 °C, 64% yield, ^{1}H NMR (400 MHz, $CDCl_3$) δ 7.68 (m, 2H), 7.59 – 7.52 (m, 5H), 7.36 (d, *J* = 1.7 Hz, 1H). ^{13}C NMR (100 MHz, $CDCl_3$) δ 144.6, 138.0, 129.8, 129.4, 129.4, 128.1, 126.3, 125.9, 124.7, 120.9, 116.8. HRMS (ESI) calculated for $C_{13}H_9Cl_2N_2$ $[M+H]^+$ 263.0137, found 263.0141.

3,7-dichloro-2-phenyl-2*H*-indazole (3c)

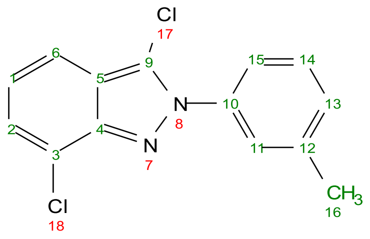

Blown oil, 70% yield, ^{1}H NMR (600 MHz, $CDCl_3$) δ 7.57 (dd, *J* = 8.5, 0.9 Hz, 1H, 6), 7.54 (s, 1H, 11), 7.50 (d, *J* = 8.2 Hz, 1H, 15), 7.45 (t, *J* = 7.7 Hz, 1H, 14), 7.41 (dd, *J* = 7.1, 0.9 Hz, 1H, 2), 7.35 (d, *J* = 7.8 Hz, 1H, 13), 7.11 (dd, *J* = 8.5, 7.2 Hz, 1H, 1), 2.49 (s, 3H, 16).^{13}C NMR (150 MHz, $CDCl_3$) δ 145.91 (4), 139.49 (12), 138.05 (10), 130.30 (13), 128.86 (14), 126.78 (2), 126.62 (11), 123.34 (3), 122.97 (15), 122.96 (1), 120.98 (5), 120.84 (9), 117.95 (6), 21.38 (16). HRMS (ESI) calculated for $C_{13}H_{11}Cl_2N_2$ $[M+H]^+$ 277.0294, found 277.0294.

3,5,7-trichloro-2-phenyl-2*H*-indazole (3d)

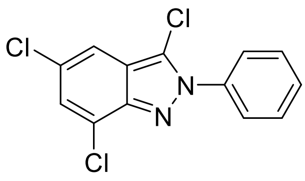

Blown solid, m.p. 177.5-178.8 °C, 66% yield, ^{1}H NMR (400 MHz, $CDCl_3$) δ 7.71 – 7.67 (m, 2H), 7.60 – 7.53 (m, 4H), 7.37 (d, *J* = 1.6 Hz, 1H). ^{13}C NMR (100 MHz, $CDCl_3$) δ 144.65, 137.98, 129.86, 129.39, 128.13, 125.96, 124.98, 124.71, 120.93, 120.67, 116.88. HRMS (ESI) calculated for $C_{13}H_8Cl_3N_2$ $[M+H]^+$ 296.9748, found 296.9750.

3-bromo-5,7-dichloro-2-phenyl-2*H*-indazole (3e)

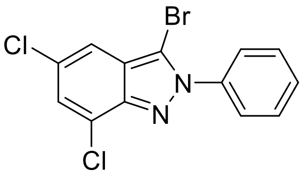

White solid, m.p. 188.2-190.8 °C,56% yield, ^{1}H NMR (600 MHz, $CDCl_3$) δ 7.70 – 7.64 (m, 2H), 7.60 – 7.52 (m, 5H). ^{13}C NMR (150 MHz, $CDCl_3$) δ 160.6, 146.0, 131.3, 131.1, 127.6, 127.3, 122.5, 117.4, 114.5, 114.5, 111.0, 55.8. HRMS (ESI) calculated for $C_{13}H_8BrCl_2N_2$ $[M+H]^+$ 340.9242, found 340.9346.

3-bromo-5,7-dichloro-2-(4-methoxyphenyl)-2*H*-indazole (3f)

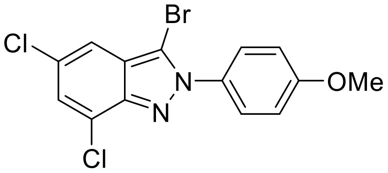

White solid, m.p. 174.5-178.2 °C,44% yield, ^{1}H NMR (600 MHz, $CDCl_3$) δ 7.69 – 7.29 (m, 4H), 7.04 (d, *J* = 6.8 Hz, 2H), 3.88 (s, 3H). ^{13}C NMR (150 MHz, $CDCl_3$) δ 160.6, 146.0, 131.3, 131.1, 127.6, 127.23, 122.5, 117.4, 114.5, 114.5, 111.0, 55.8. HRMS (ESI) calculated for $C_{14}H_{10}BrCl_2N_2O$ $[M+H]^+$ 370.9348, found 370.9352.

7-bromo-3-chloro-2-phenyl-2*H*-indazole (3g)

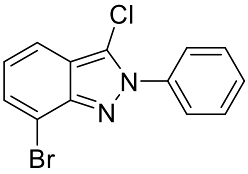

White solid, m.p. 174.5-178.2 °C,65% yield, ^{1}H NMR (600 MHz, $CDCl_3$) δ 7.73 – 7.64 (m, 3H), 7.61 – 7.49 (m, 5H). ^{13}C NMR (150 MHz, $CDCl_3$) δ 145.8, 138.0, 133.4, 129.8, 129.4, 126.1, 126.0, 120.8, 118.7, 115.6, 113.1. HRMS (ESI) calculated for $C_{13}H_9BrClN_2$ $[M+H]^+$ 306.9632, found 306.9634.

7-bromo-3,5-dichloro-2-phenyl-2*H*-indazole (3h)

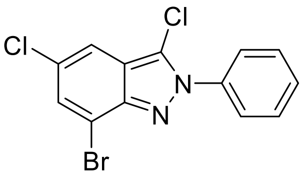

White solid, m.p. 182.8-184.7 °C,68% yield, ^{1}H NMR (600 MHz, $CDCl_3$) δ 7.71 – 7.66 (m, 2H), 7.58 – 7.52 (m, 5H). ^{13}C NMR (150 MHz, $CDCl_3$) δ 145.7, 138.0, 131.3, 129.8, 129.4, 128.4, 126.4, 126.0, 120.5, 117.4, 113.0. HRMS (ESI) calculated for $C_{13}H_8BrCl_2N_2$ $[M+H]^+$ 340.9242, found 340.9246.

7-bromo-3,5-dichloro-2-phenyl-2*H*-indazole (3i)

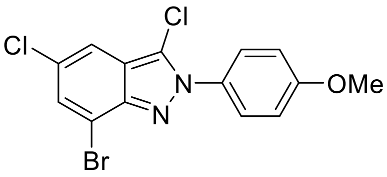

Purple solid, m.p. 178.1-179.9 °C,69% yield, ^{1}H NMR (600 MHz, $CDCl_3$) δ 7.60 – 7.51 (m, 4H), 7.05 (s, 1H), 7.03 (d, *J* = 2.0 Hz, 1H), 3.88 (s, 3H). ^{13}C NMR (150 MHz, $CDCl_3$) δ 160.5, 145.5, 131.1, 131.0, 128.2, 127.3, 120.8, 120.3, 117.4, 114.5, 112.8, 55.8. HRMS (ESI) calculated for $C_{14}H_{10}BrCl_2N_2O$ $[M+H]^+$ 370.9348, found 370.9352.

7-bromo-3,5-dichloro-2-phenyl-2*H*-indazole (3j)

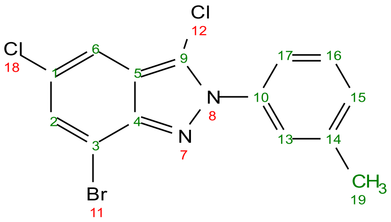

Purple solid, m.p. 163.0-163.8 °C,74% yield, ^{1}H NMR (600 MHz, $CDCl_3$) δ 7.76 (d, *J* = 1.6 Hz, 1H, 6), 7.71 (d, *J* = 1.6 Hz, 1H, 2), 7.51 – 7.48 (m, 1H, 11), 7.48 – 7.43 (m, 2H, 13, 15), 7.39 – 7.35 (m, 1H, 14), 2.49 (d, *J* = 0.8 Hz, 3H, 16).^{13}C NMR (150 MHz, $CDCl_3$) δ 146.33 (4), 139.53 (12), 138.60 (10), 133.22 (2), 130.59 (14), 128.86 (13), 126.90 (11), 124.14 (9), 123.34 (15), 121.42 (6), 115.60 (5), 112.80 (3), 106.95 (1), 21.34 (16). HRMS (ESI) calculated for $C_{14}H_{10}BrCl_2N_2$ $[M+H]^+$ 354.9399, found 354.9403.

3,5,7-tribromo-2-phenyl-2*H*-indazole (4b)

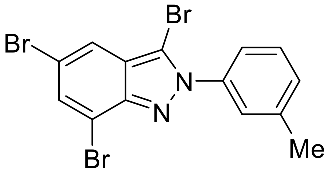

White solid, m.p. 205.1-205.3 °C, 72% yield, ^{1}H NMR (400 MHz, $CDCl_3$) δ 7.71 (s, 1H), 7.66 (s, 1H), 7.46 (s, 1H), 7.44 – 7.38 (m, 2H), 7.36 – 7.30 (m, 1H), 2.45 (s, 3H).^{13}C NMR (100 MHz, $CDCl_3$) δ 146.3, 139.6, 138.6, 133.2, 130.6, 128.9, 126.9, 124.1, 123.4, 121.5, 115.6, 112.8, 107.1, 77.2, 21.5. HRMS (ESI) calculated for $C_{14}H_{10}Br_3N_2$ $[M+H]^+$ 442.8389, found 442.8393.

2a ^{1}H-NMR

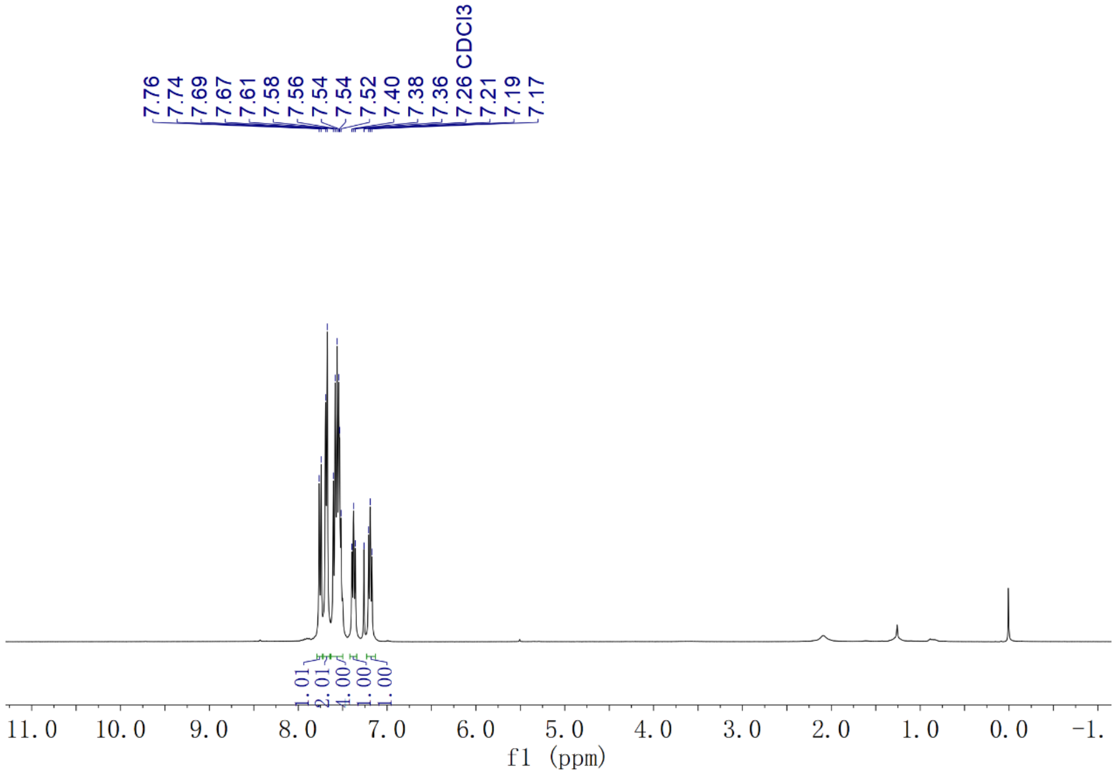

2a ^{13}C-NMR

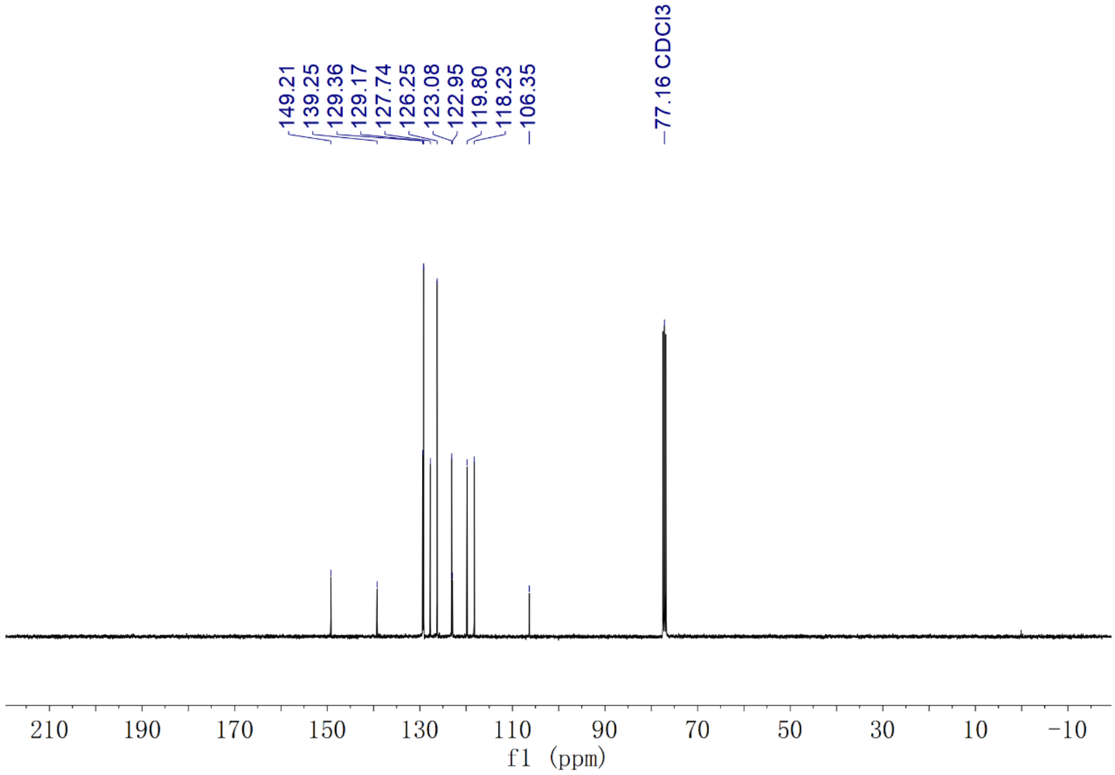

2b ^{1}H-NMR

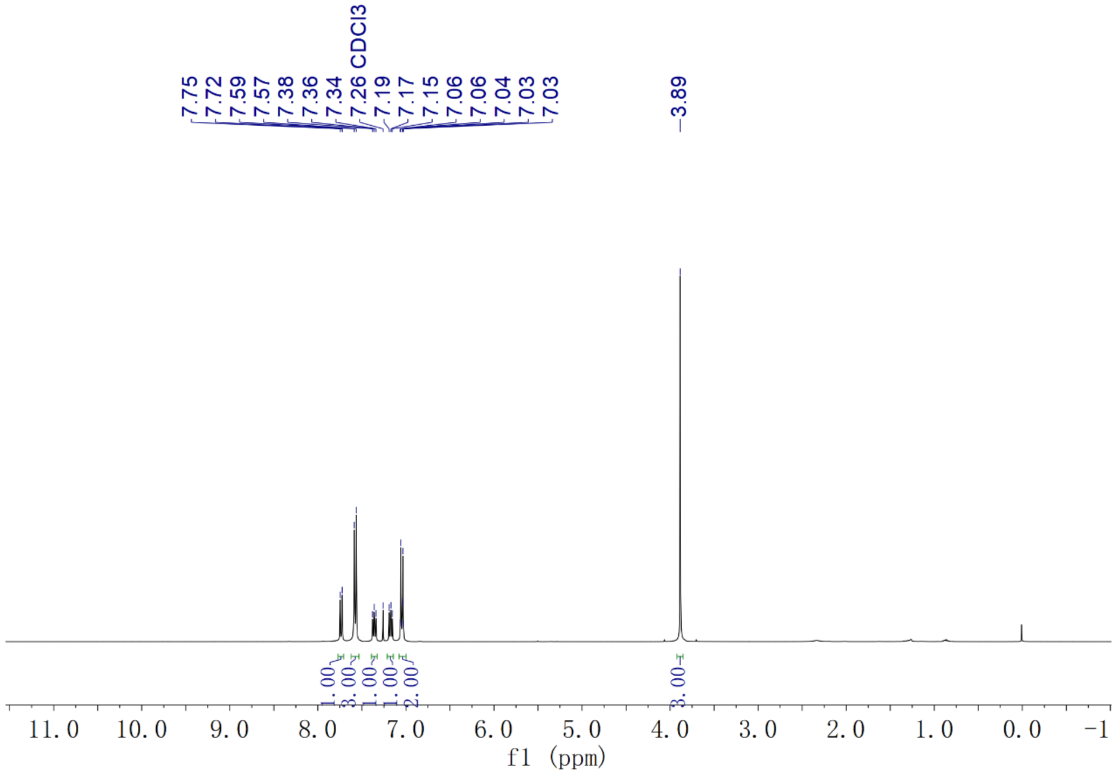

2b ^{13}C-NMR

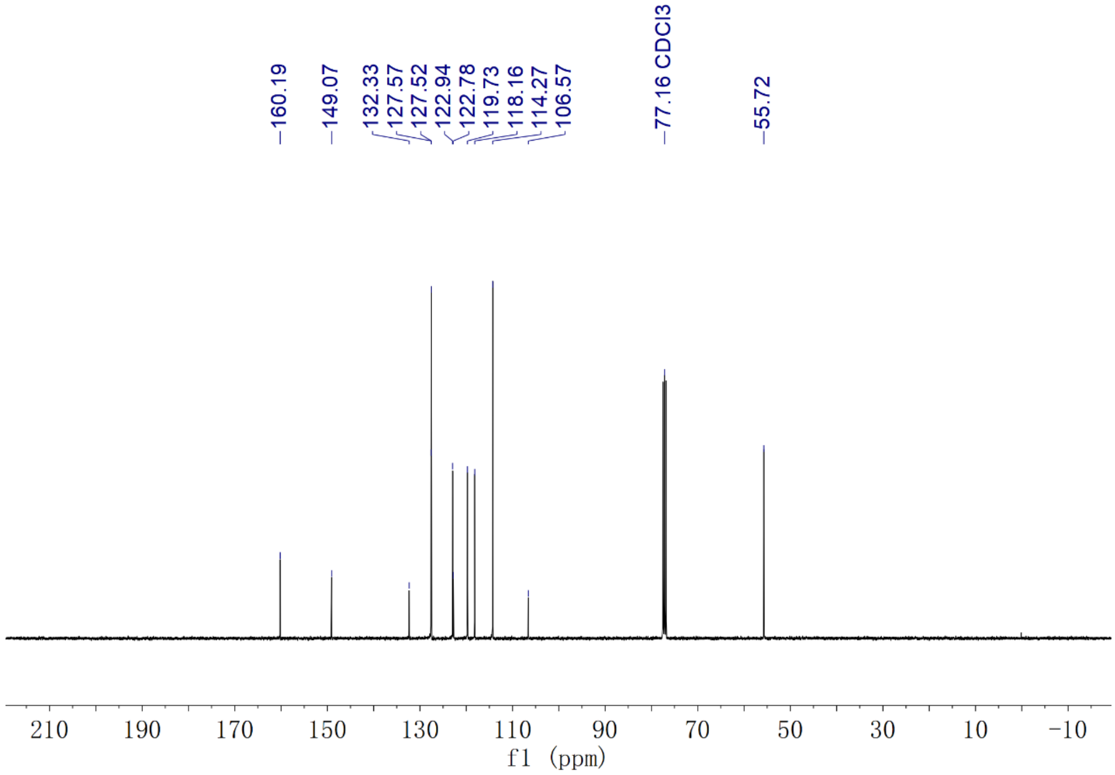

2c ^{1}H-NMR

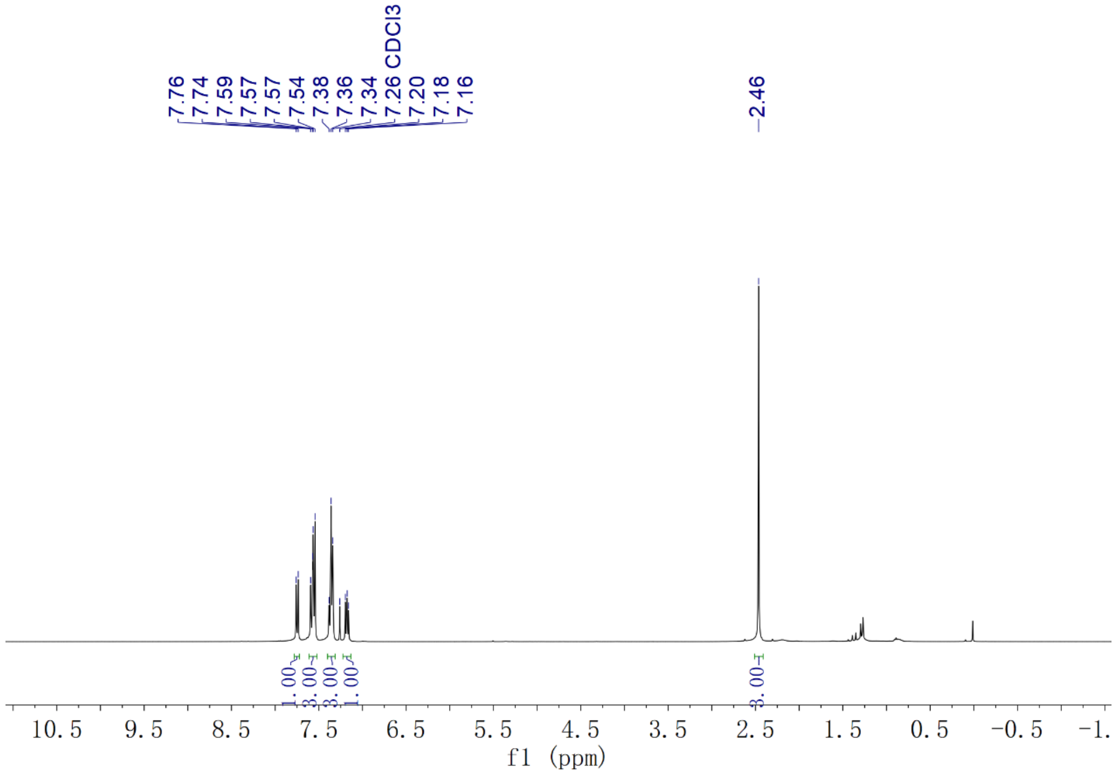

2c ^{13}C-NMR

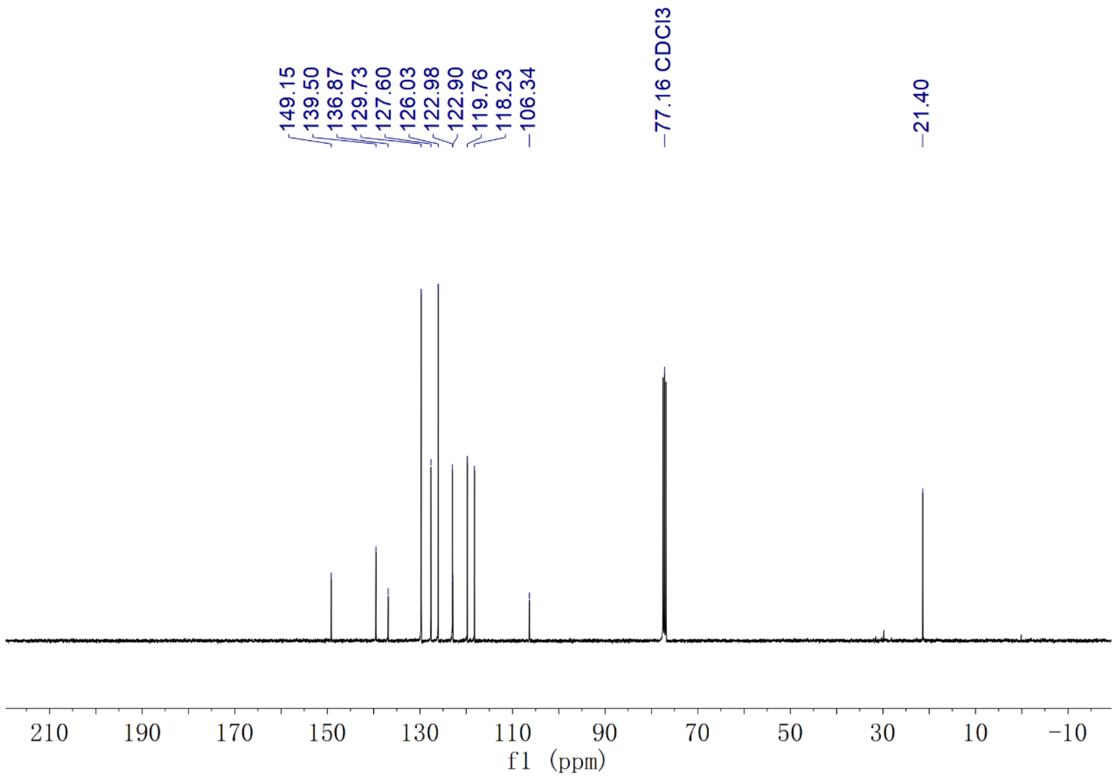

2d ¹H-NMR

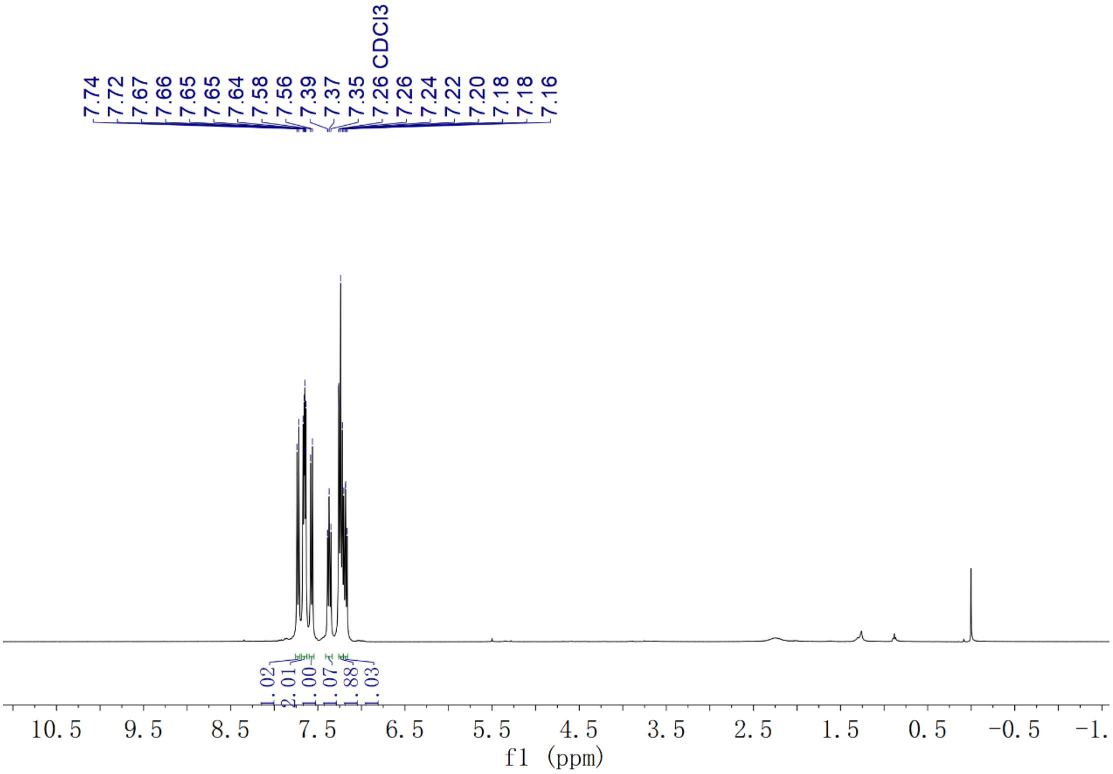

2d ¹³C-NMR

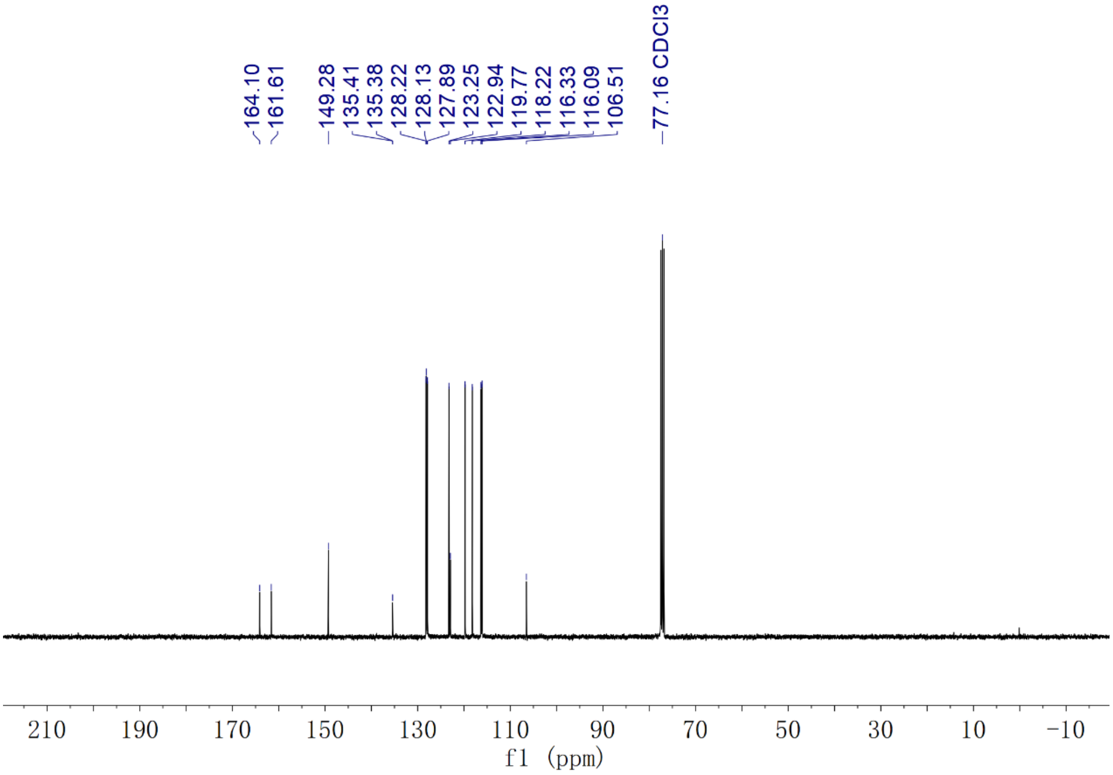

2d ^{19}F-NMR

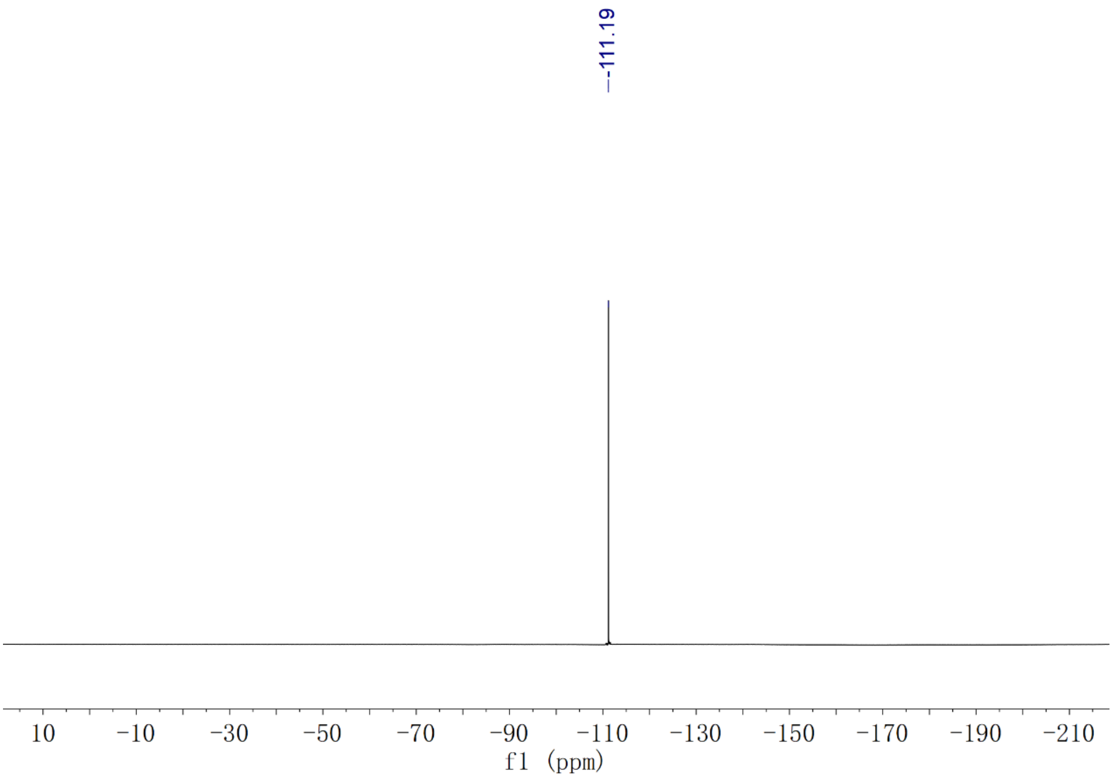

2e ^{1}H-NMR

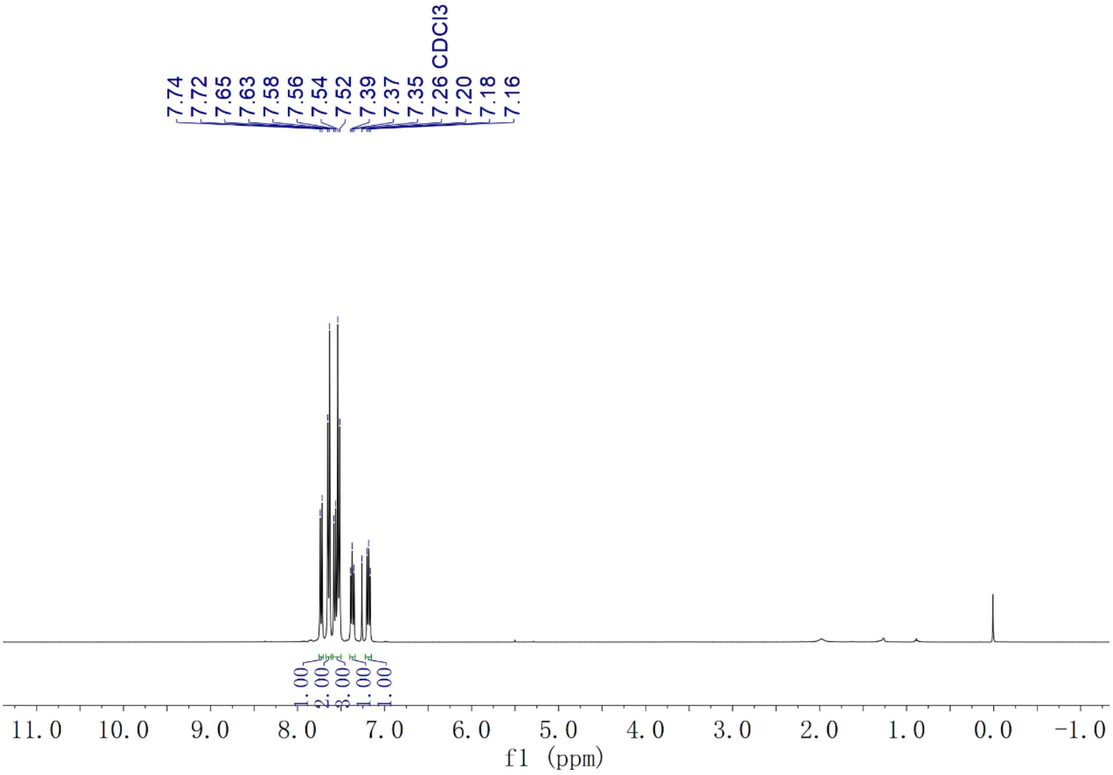

2e ^{13}C-NMR

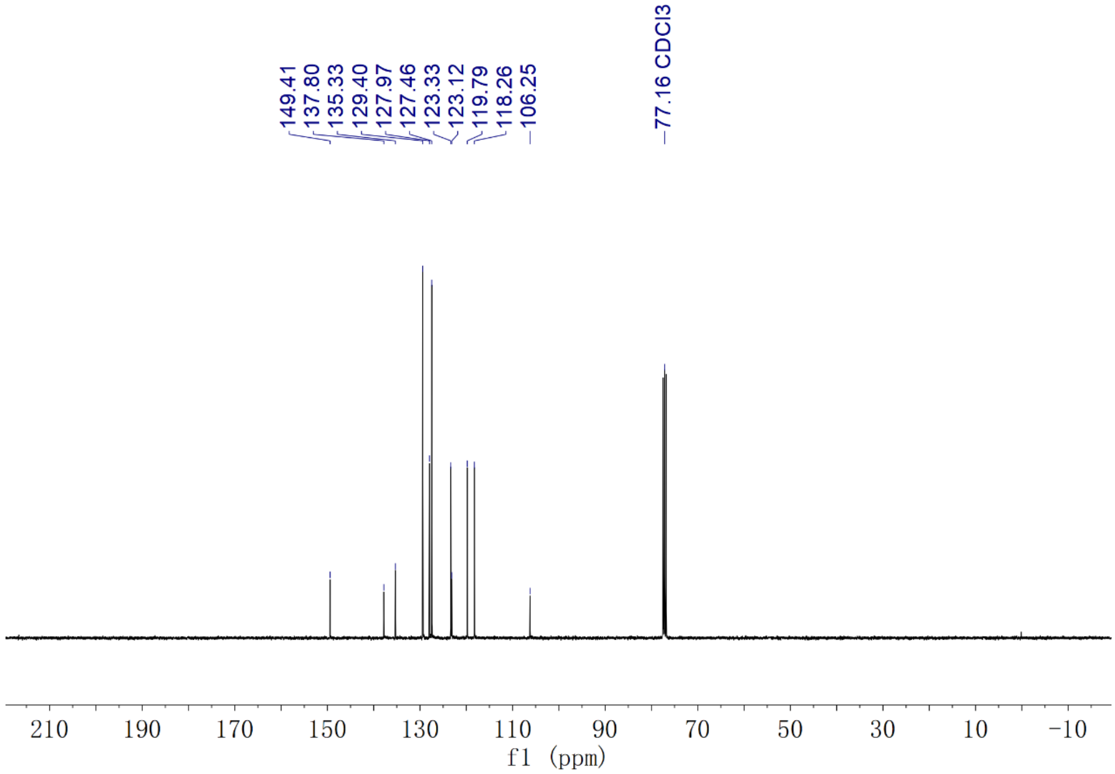

2f ^{1}H-NMR

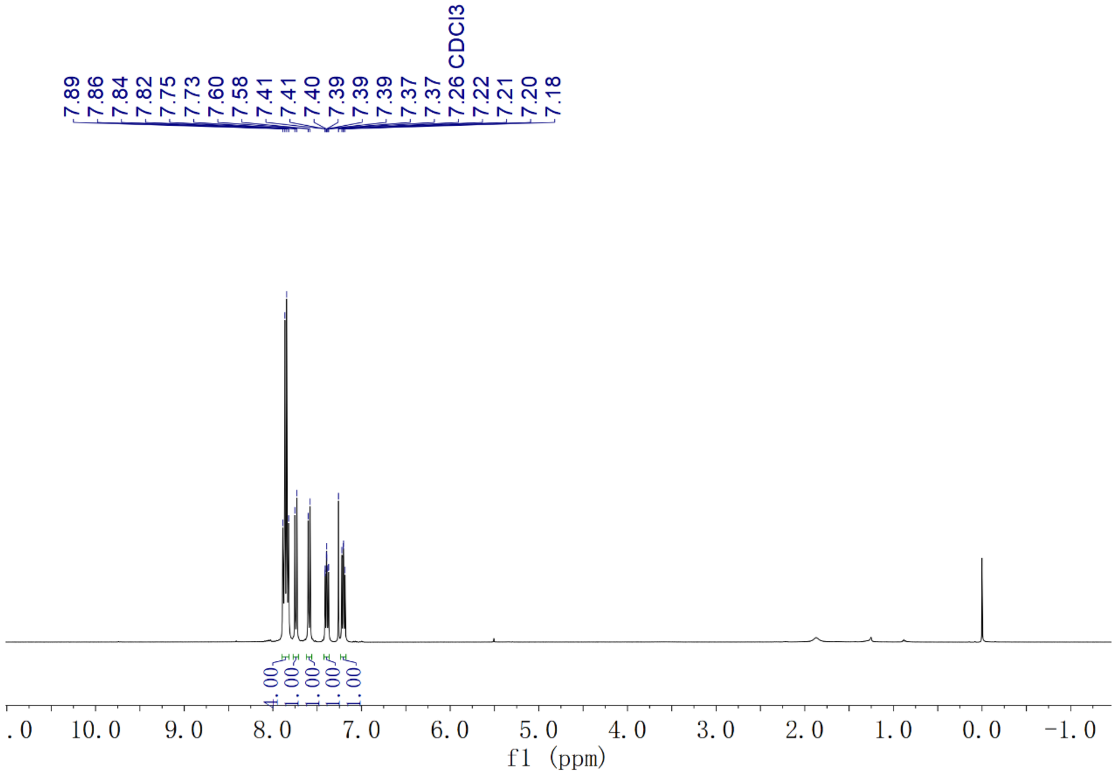

2f ^{13}C-NMR

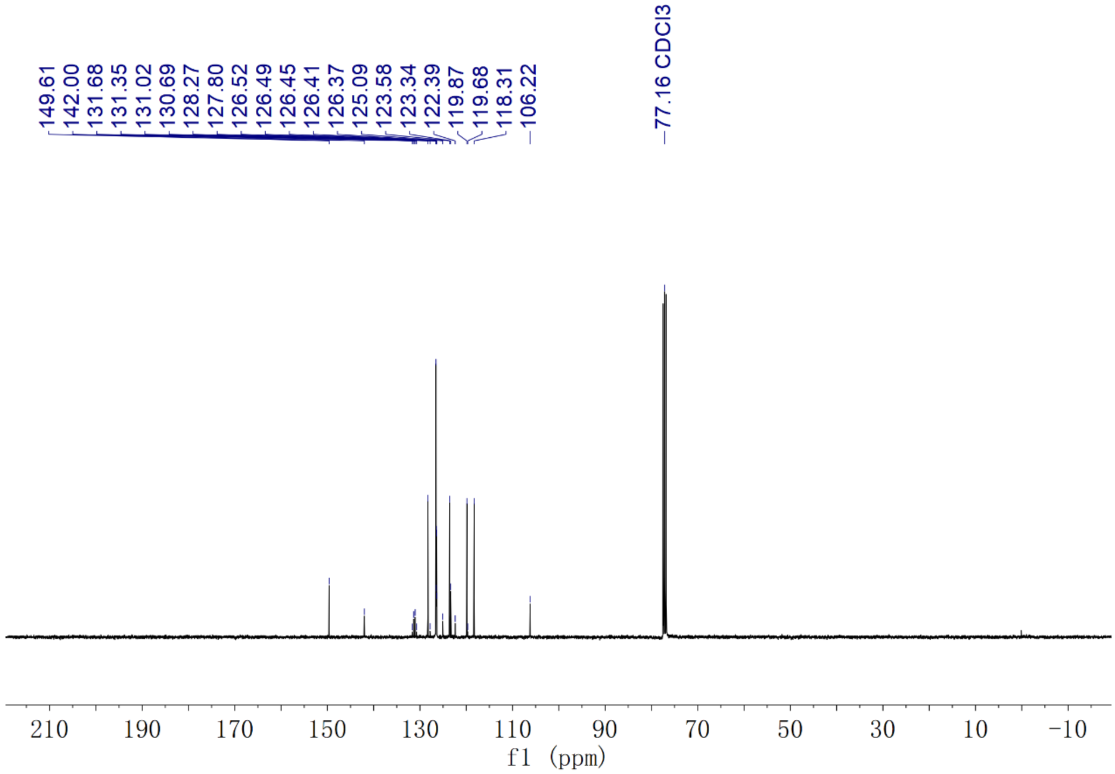

2f ^{19}F-NMR

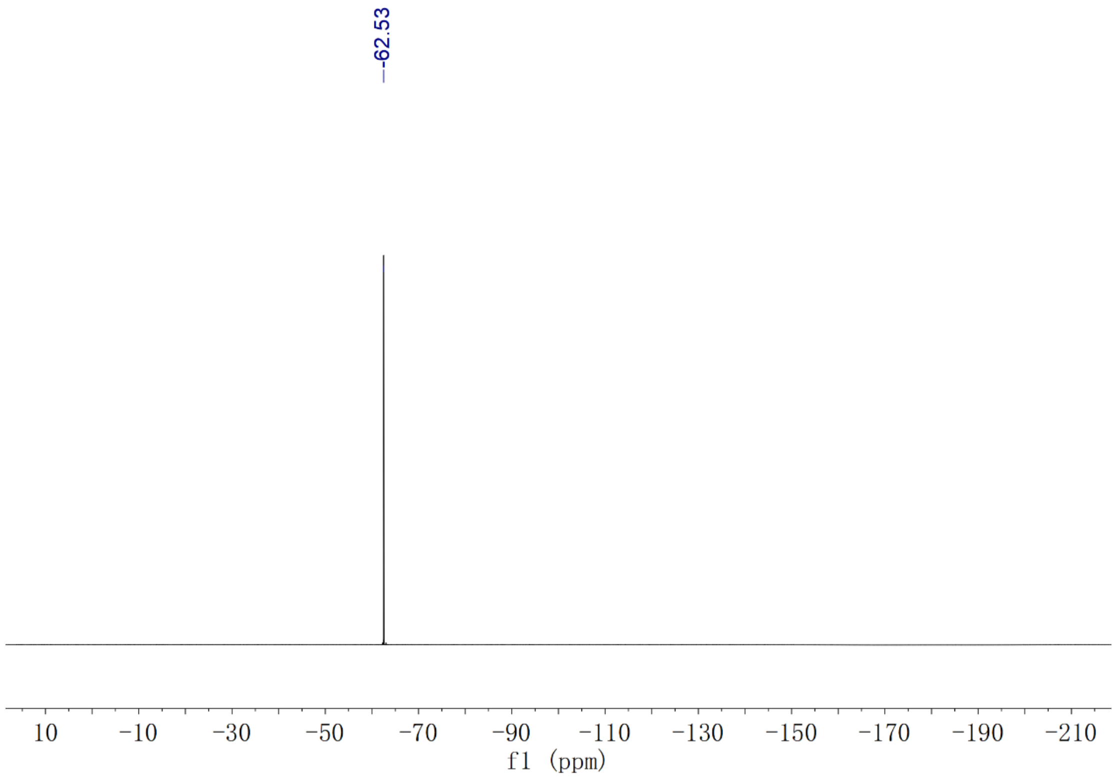

2g ^{1}H-NMR

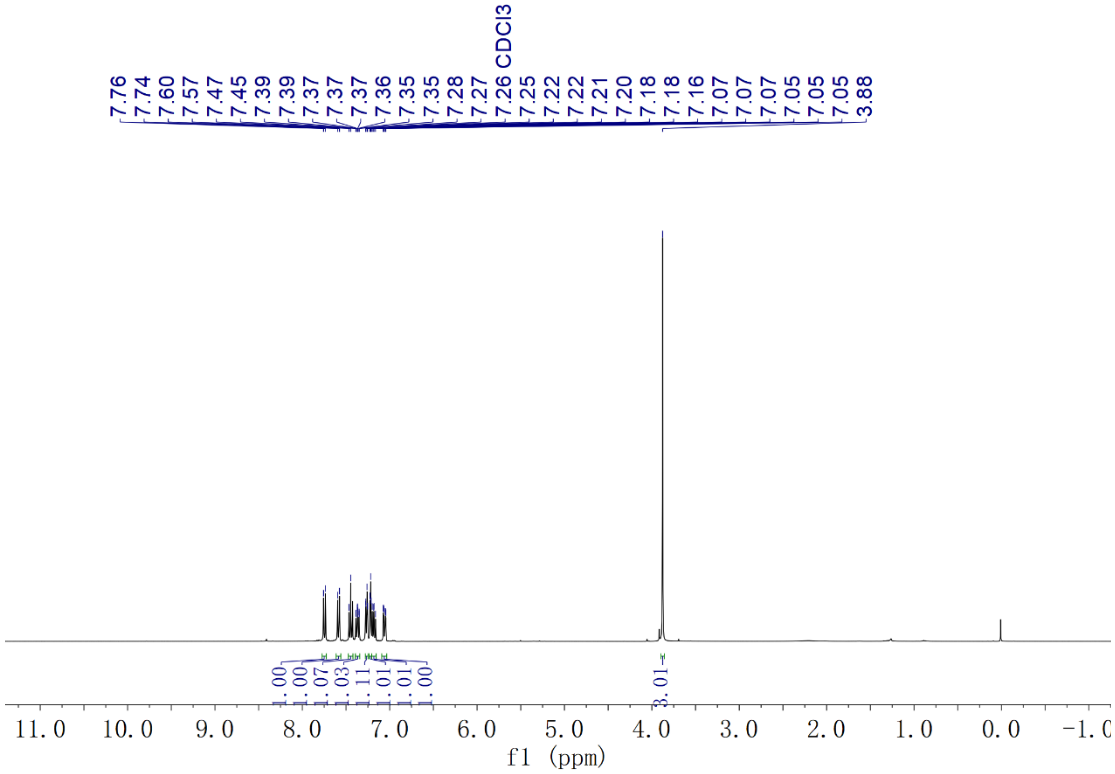

2g ^{13}C-NMR

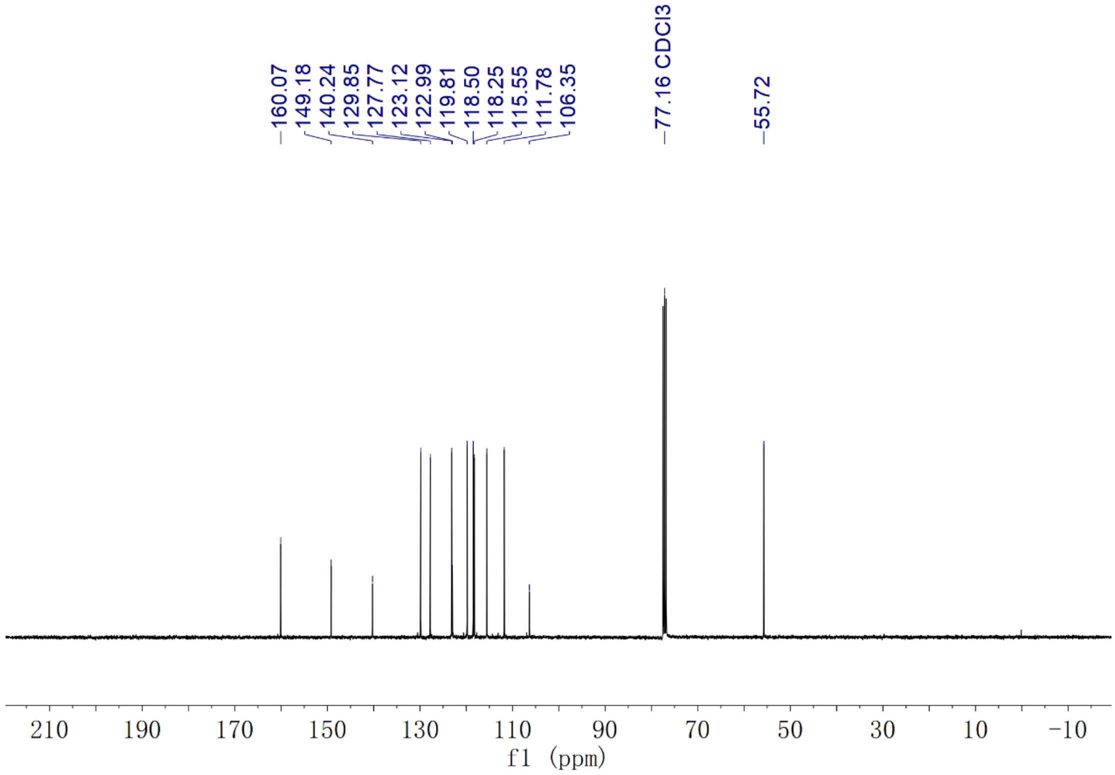

2h ^{1}H-NMR

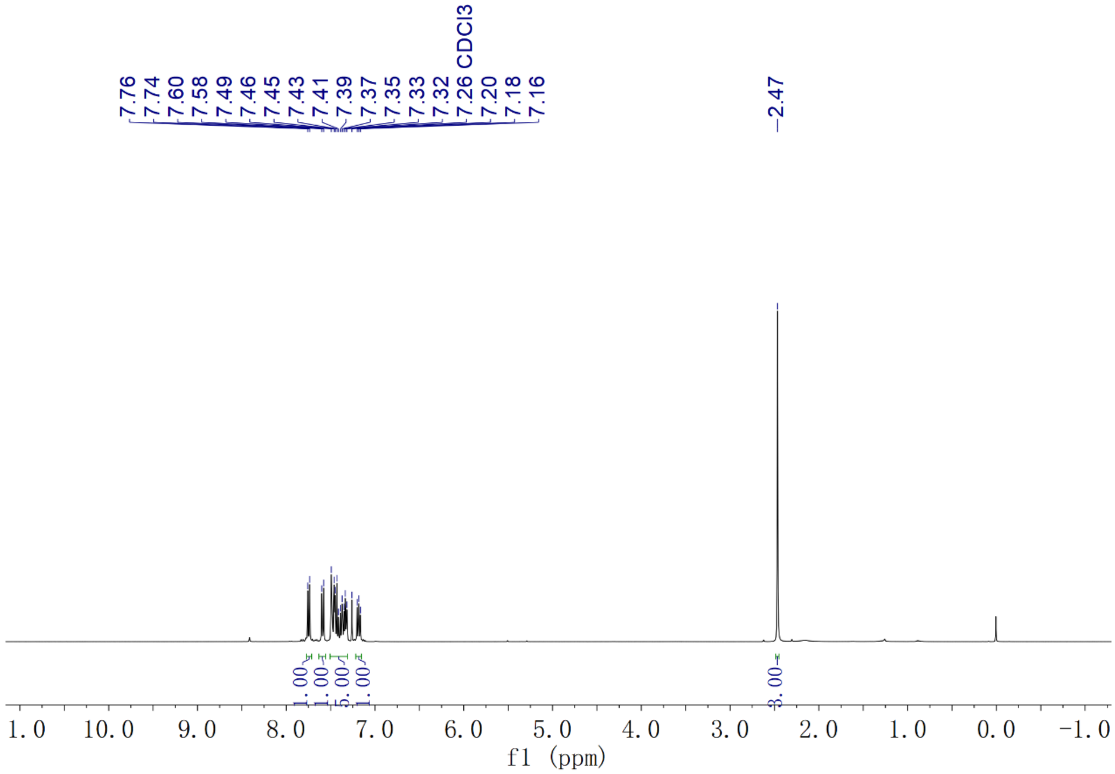

2i ^{1}H-NMR

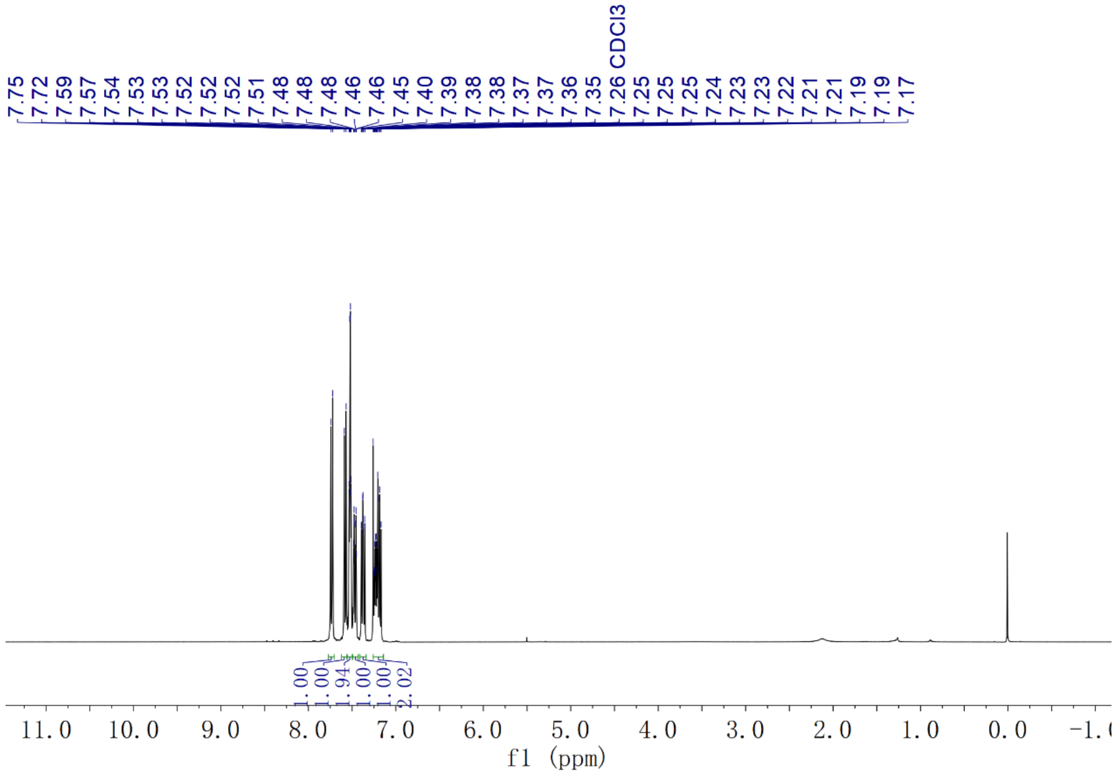

2i ^{13}C-NMR

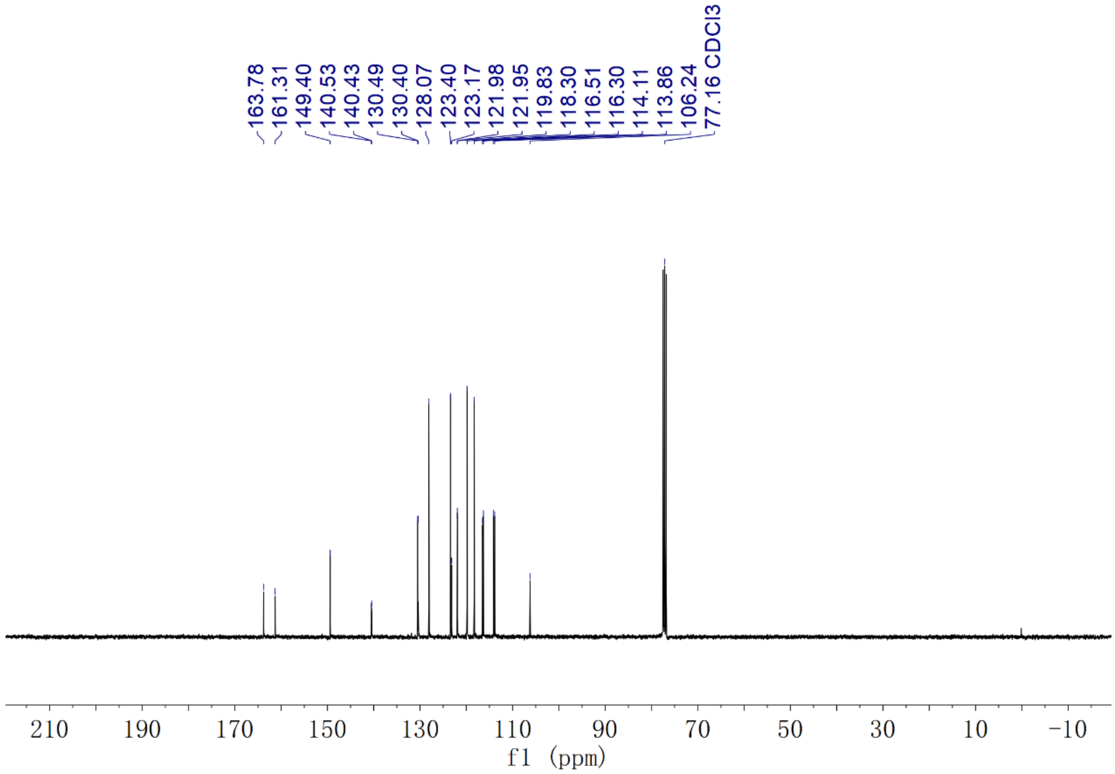

2i ^{19}F-NMR

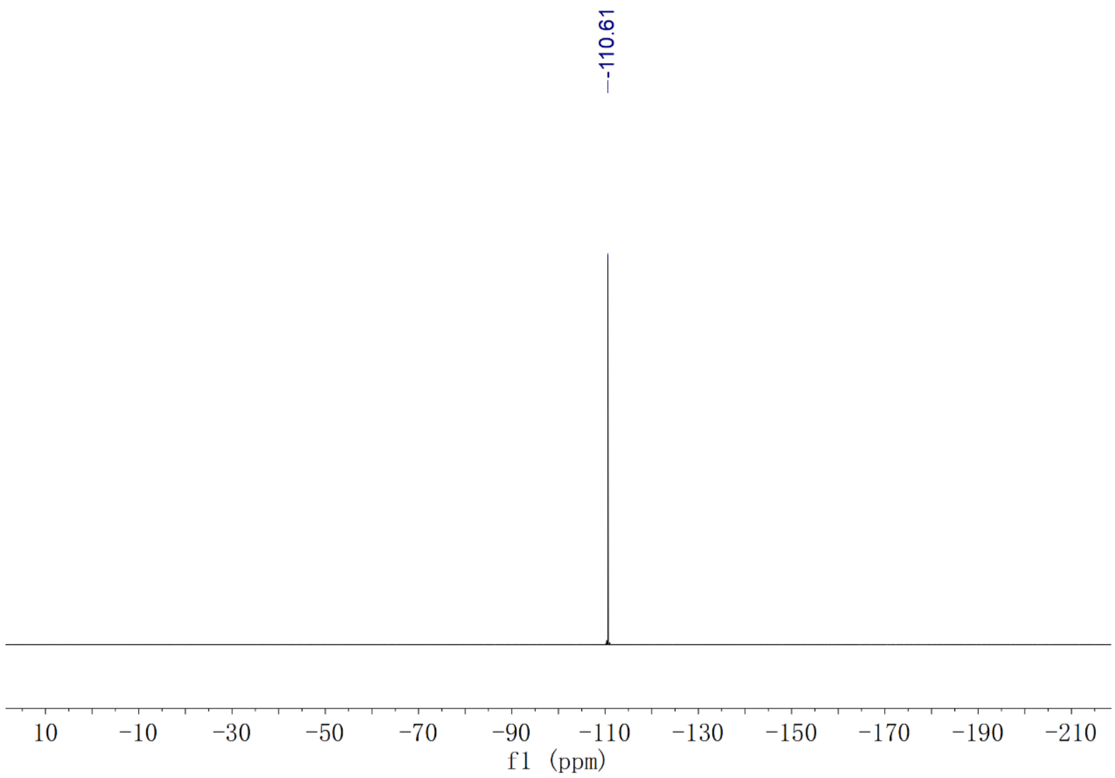

2j ^{1}H-NMR

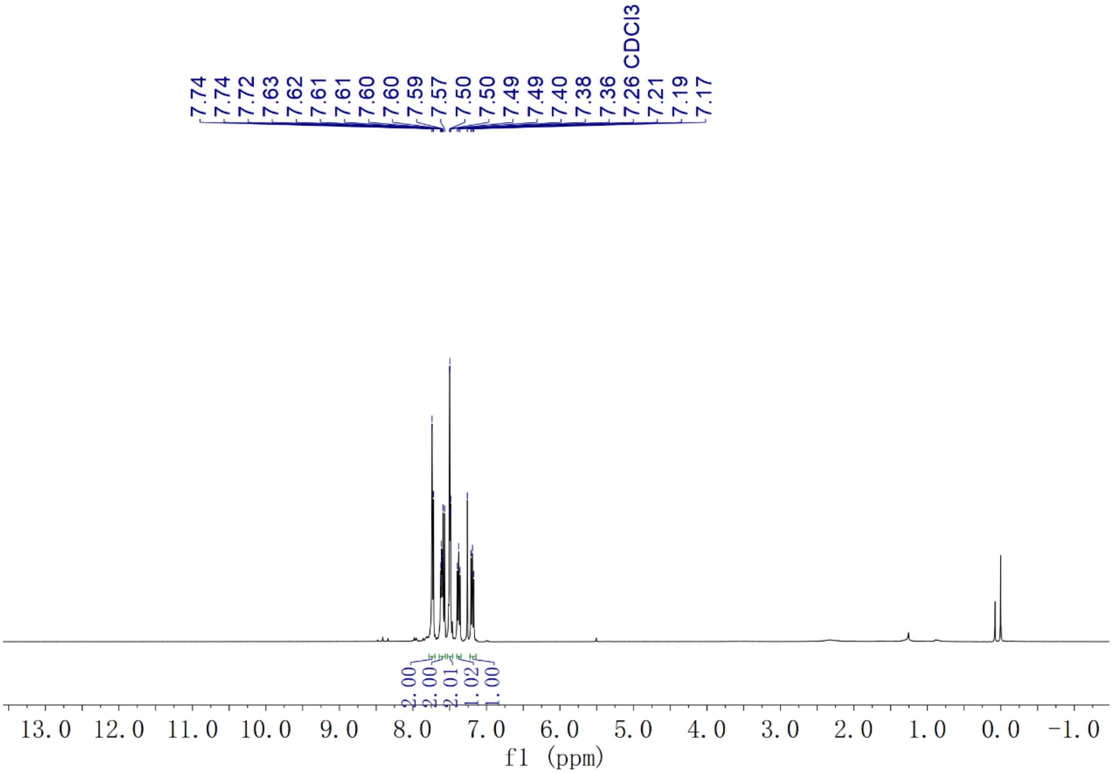

2j ^{13}C-NMR

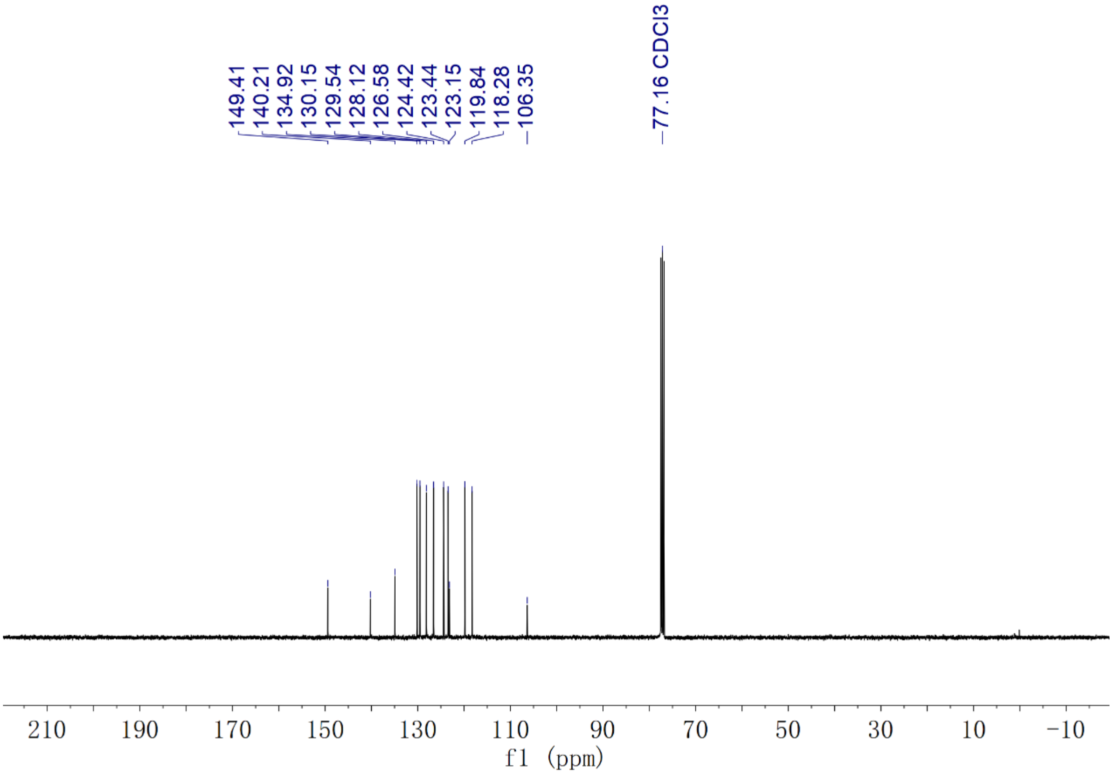

2k ^{1}H-NMR

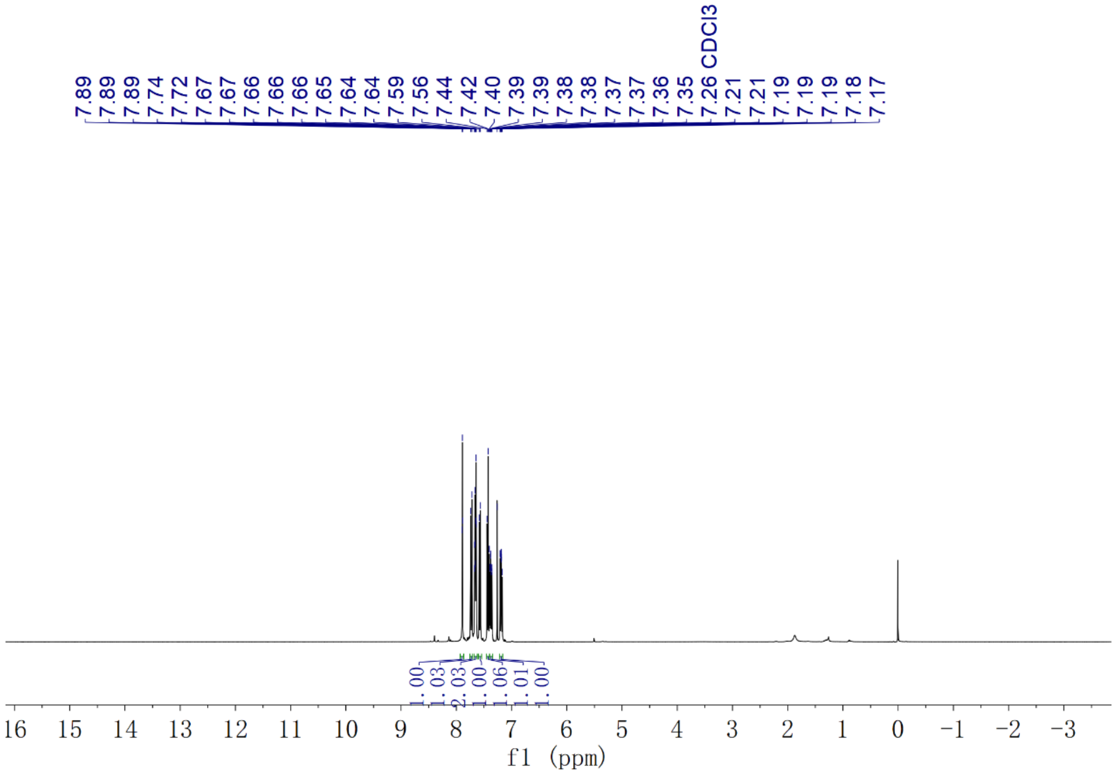

2k ^{13}C-NMR

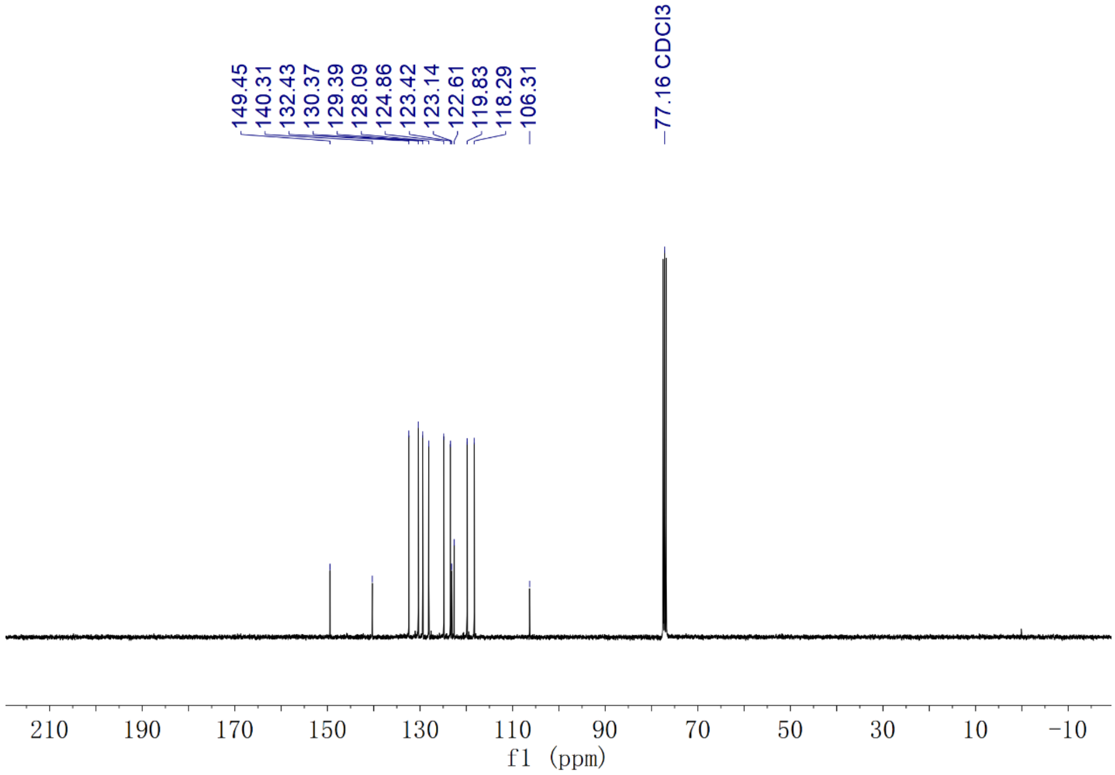

2l ^{1}H-NMR

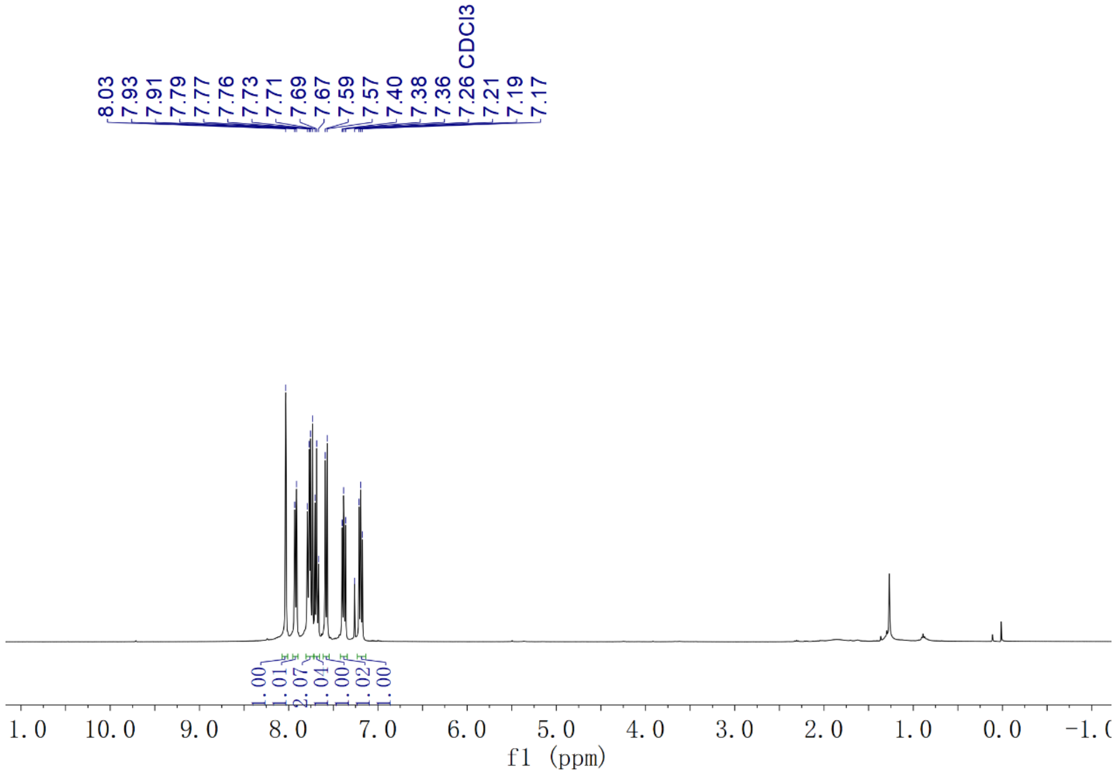

2l ^{13}C-NMR

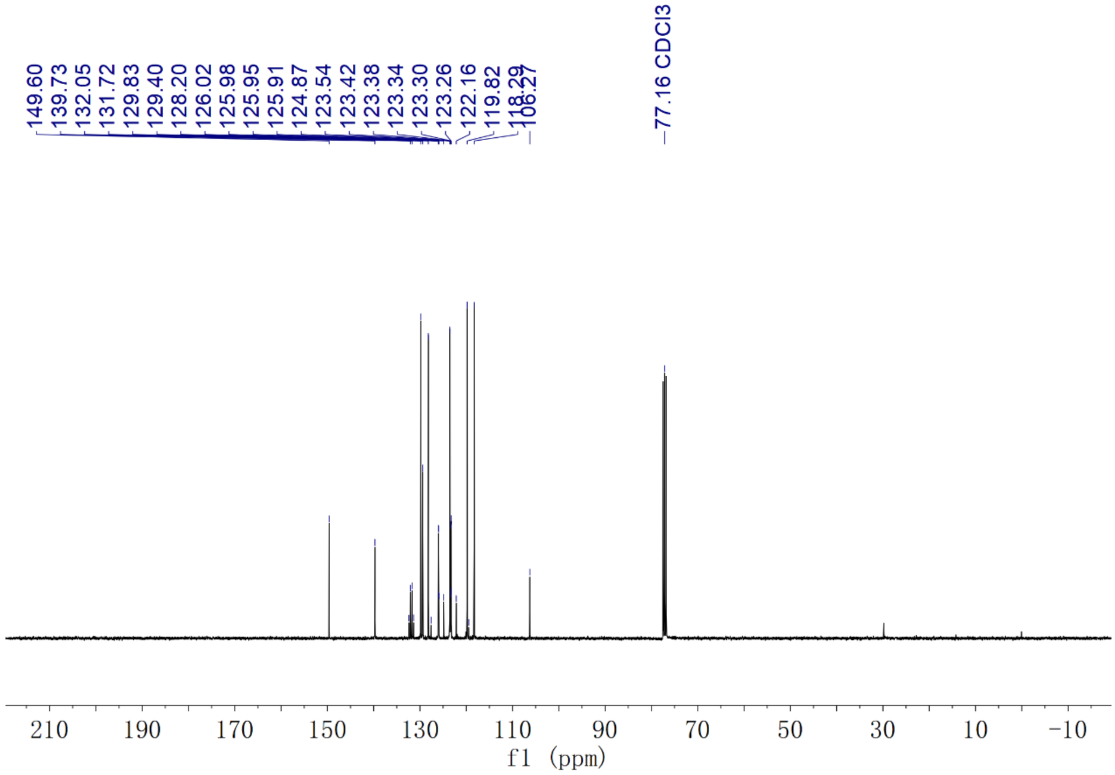

2l ^{19}F-NMR

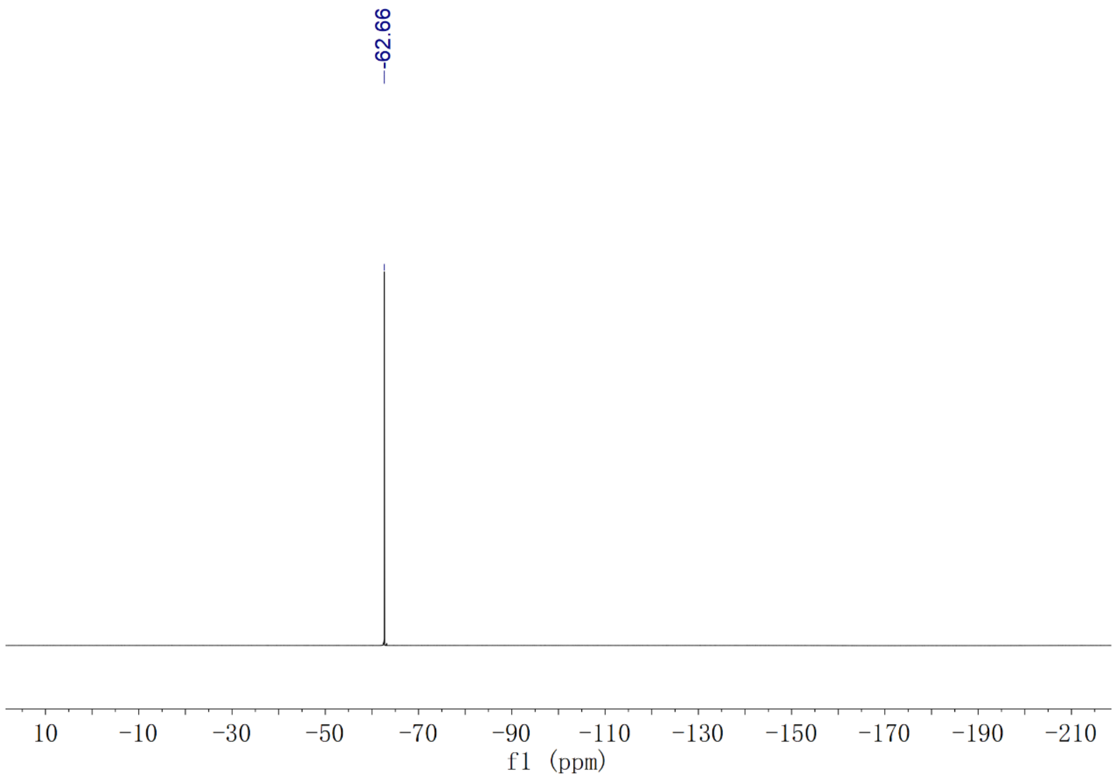

2m ^{1}H-NMR

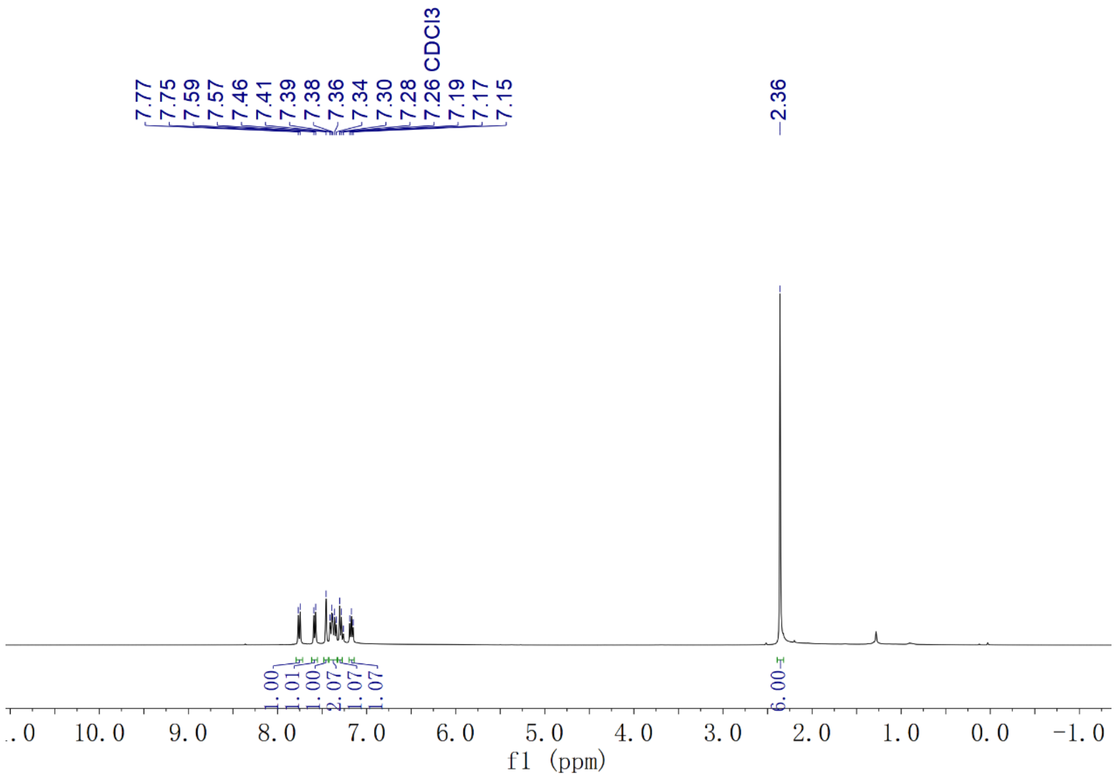

2m ^{13}C-NMR

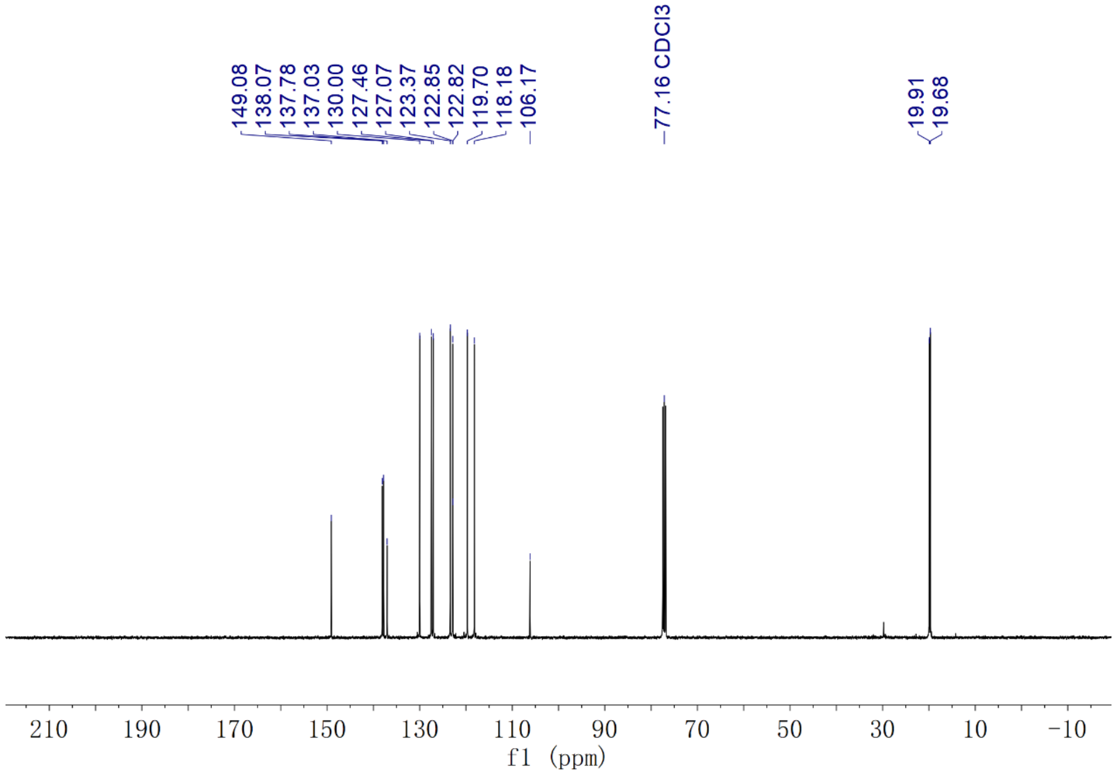

2n ^{1}H-NMR

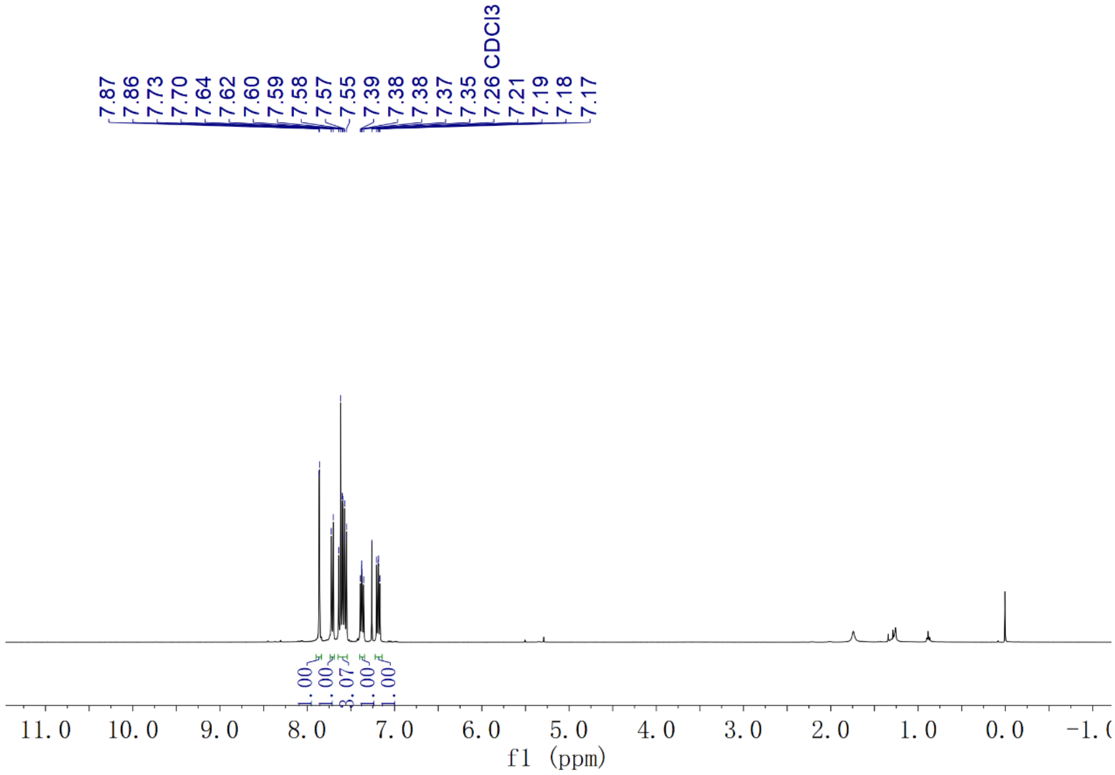

2n ^{13}C-NMR

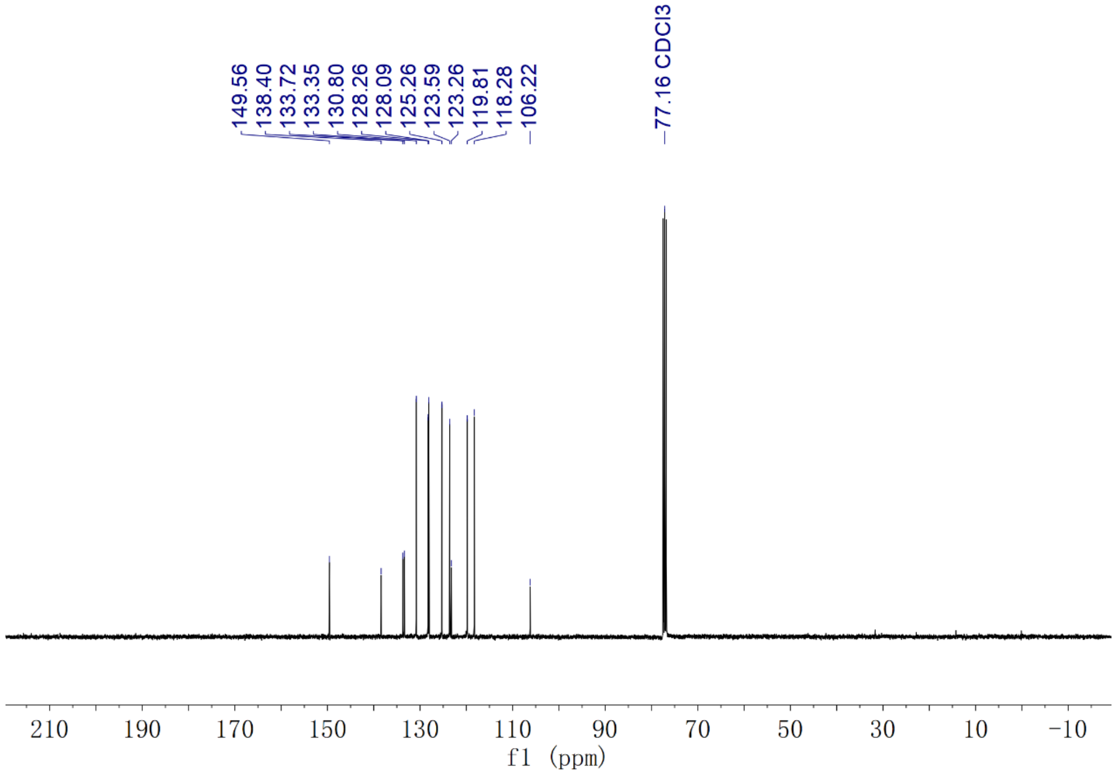

2o ^{1}H-NMR

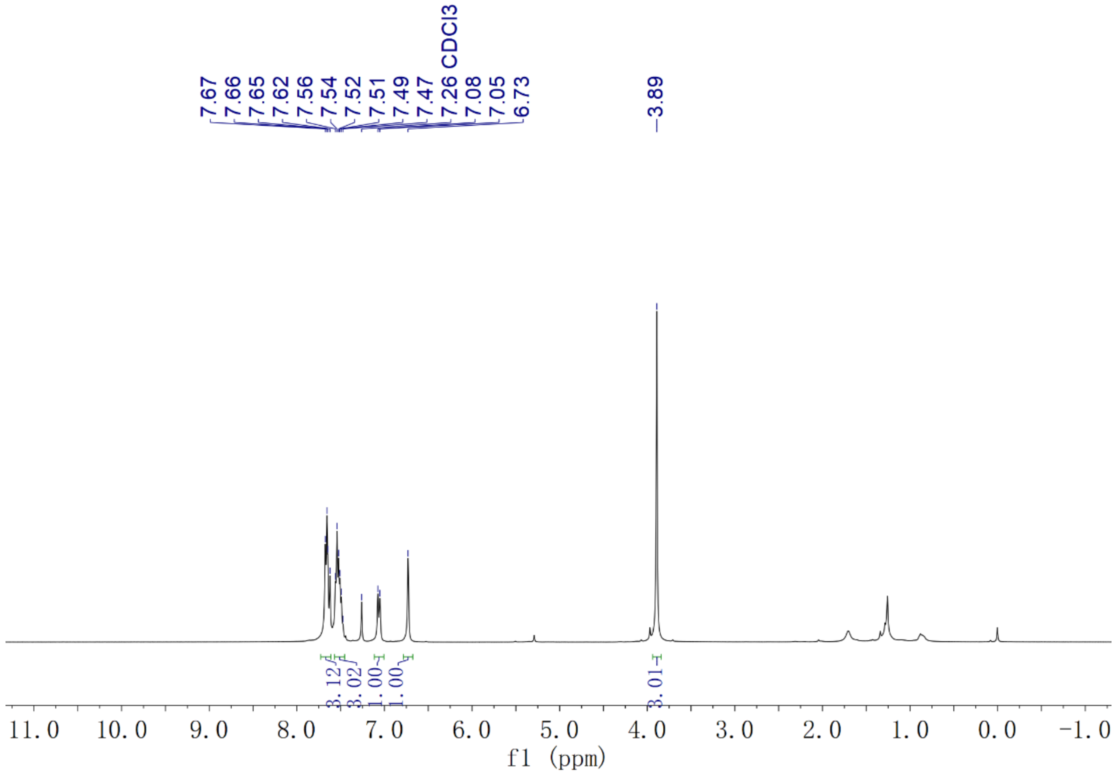

2o ^{13}C-NMR

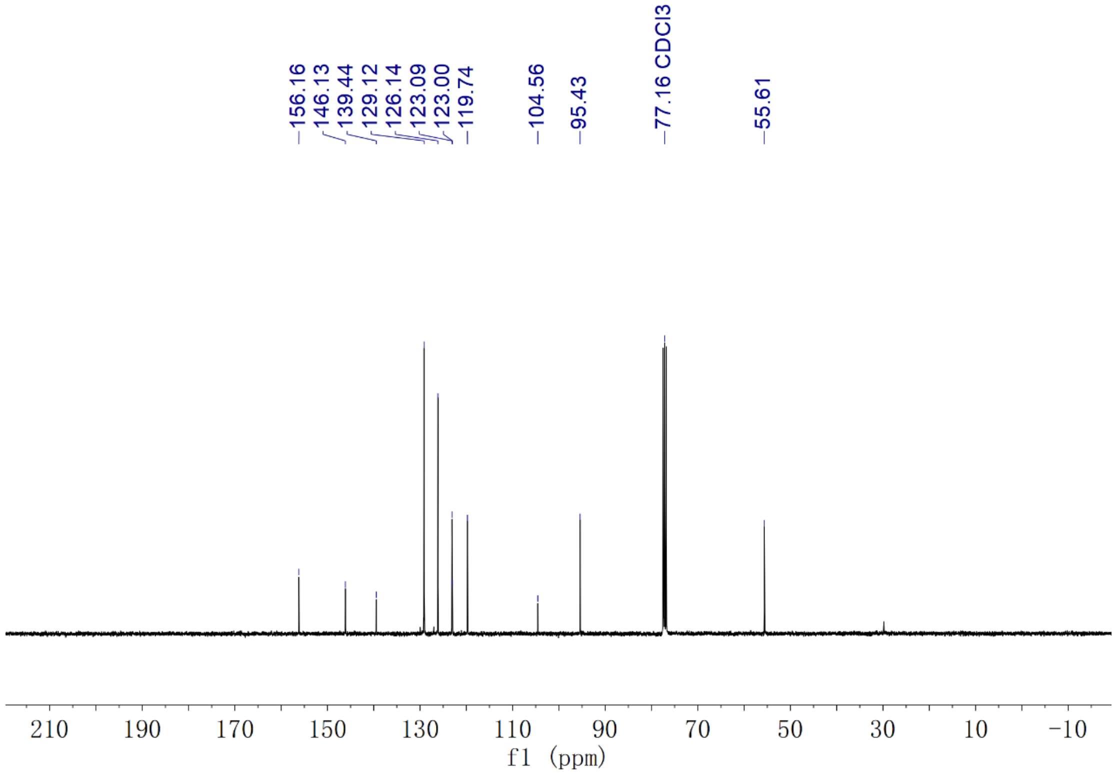

2p ^{1}H-NMR

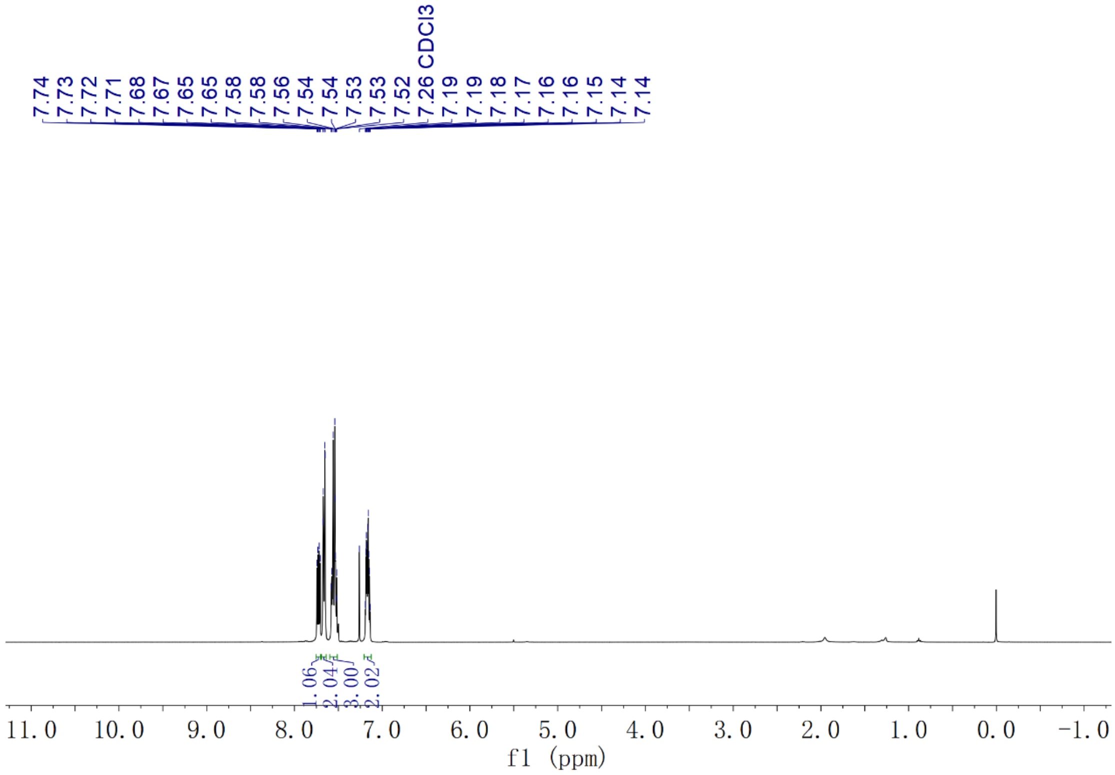

2p ^{13}C-NMR

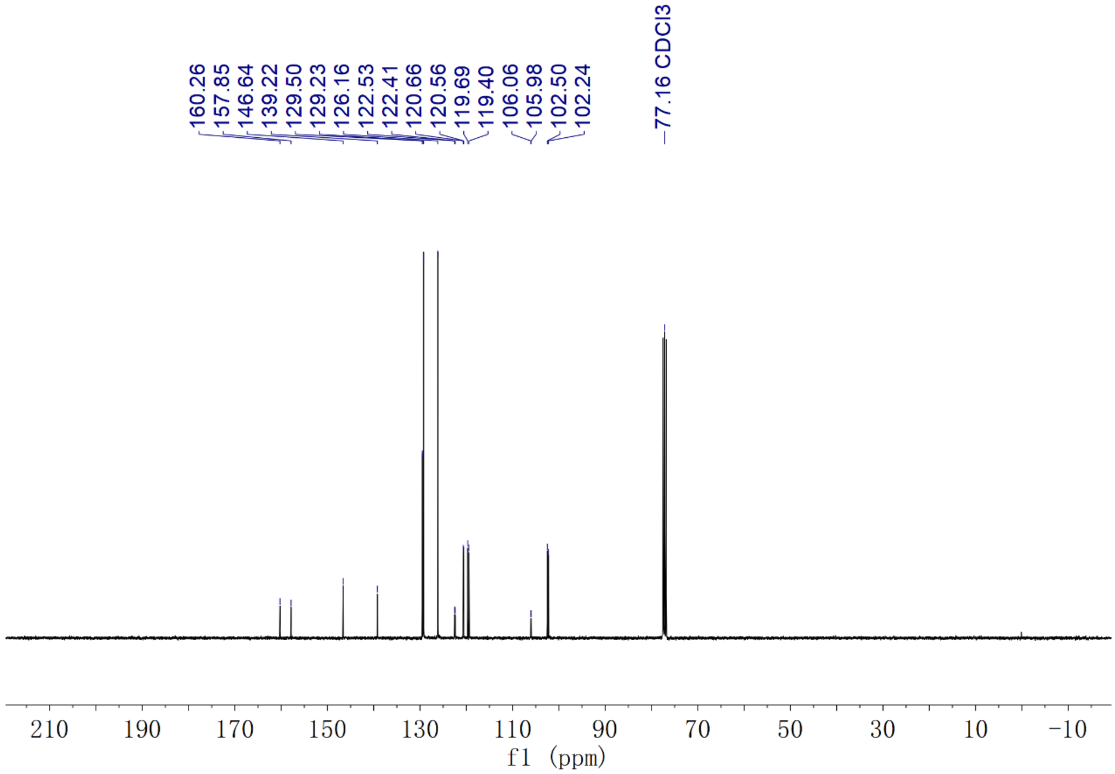

2p ^{19}F-NMR

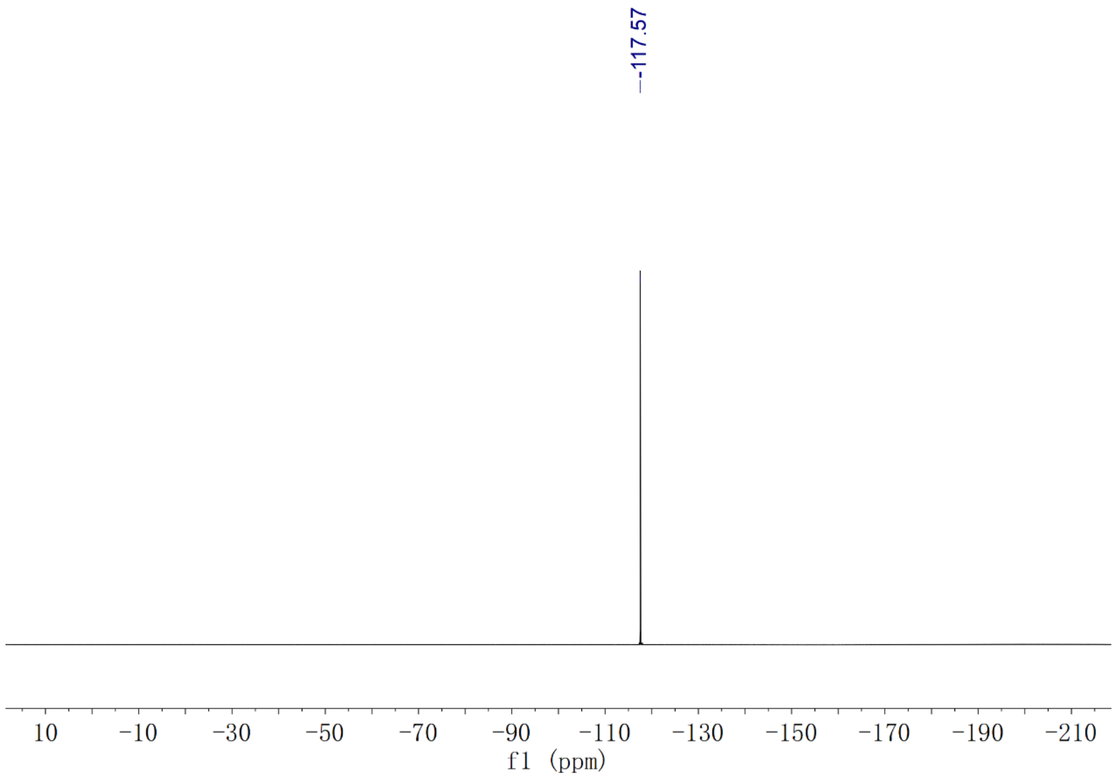

2q ^{1}H-NMR

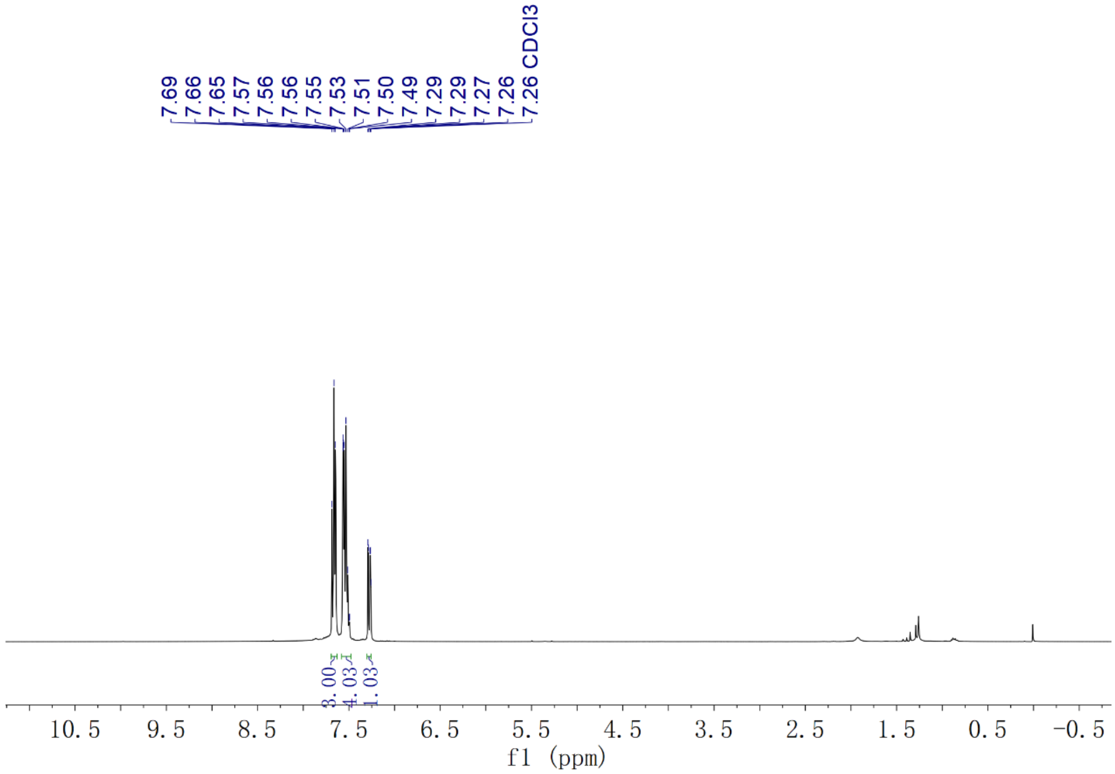

2q ^{13}C-NMR

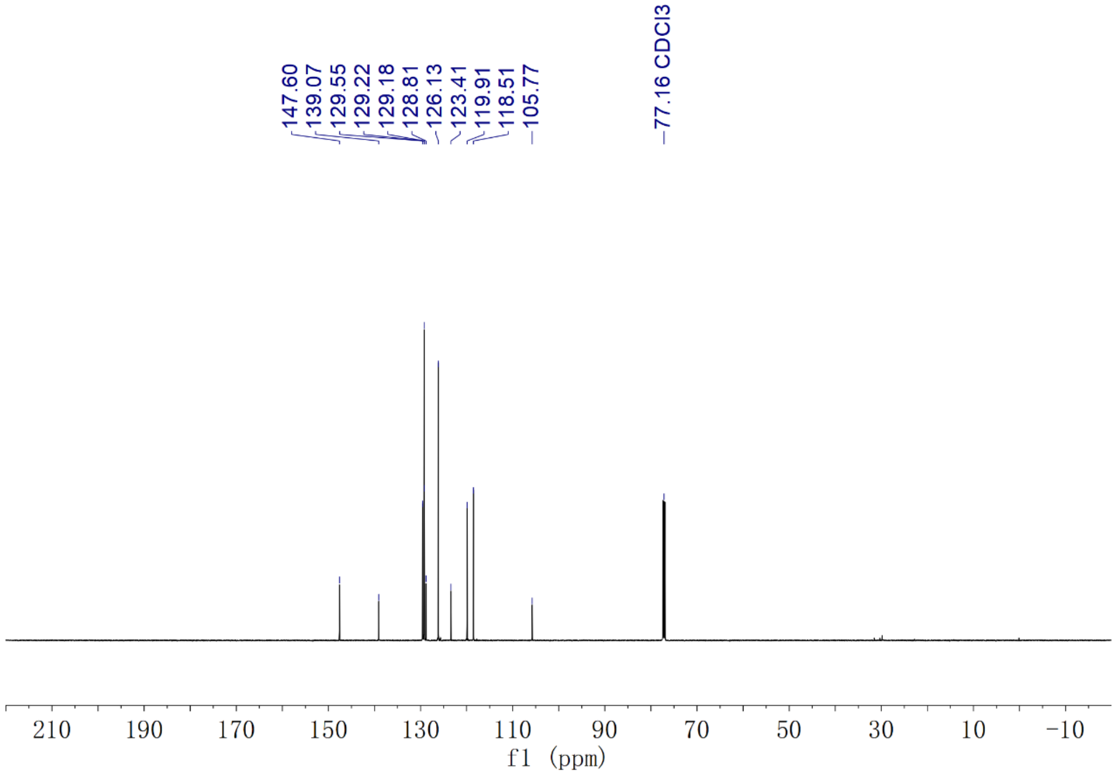

2r ^{1}H-NMR

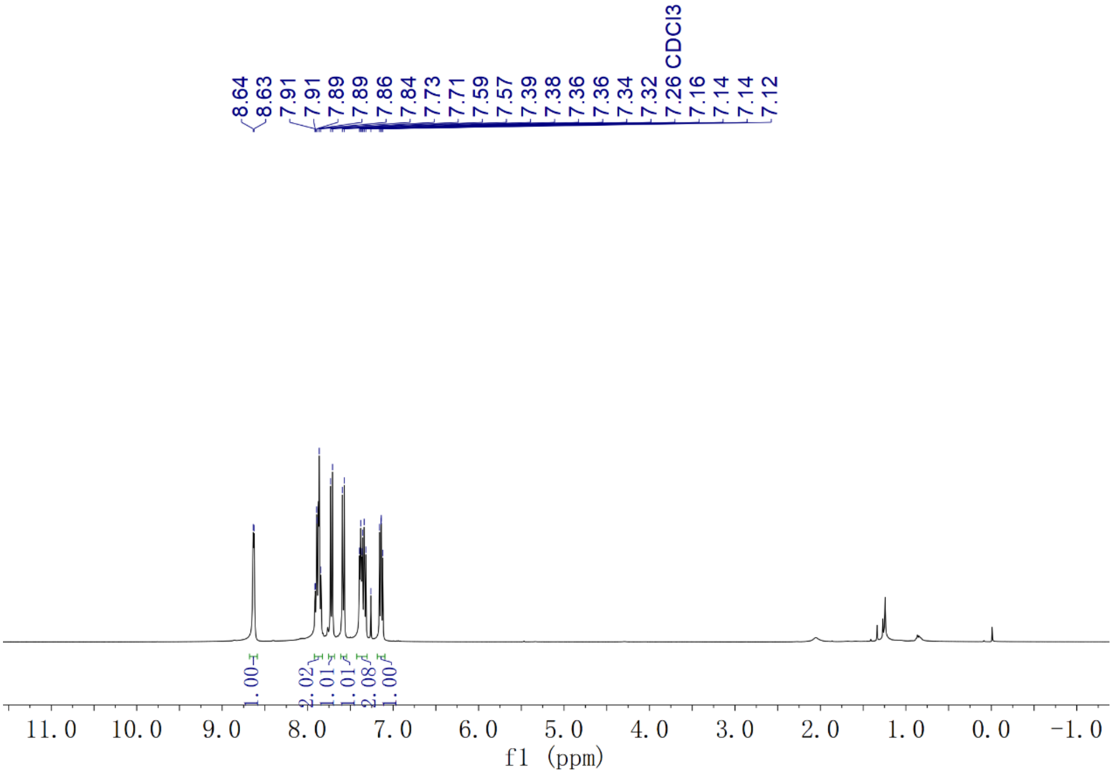

2r ^{13}C-NMR

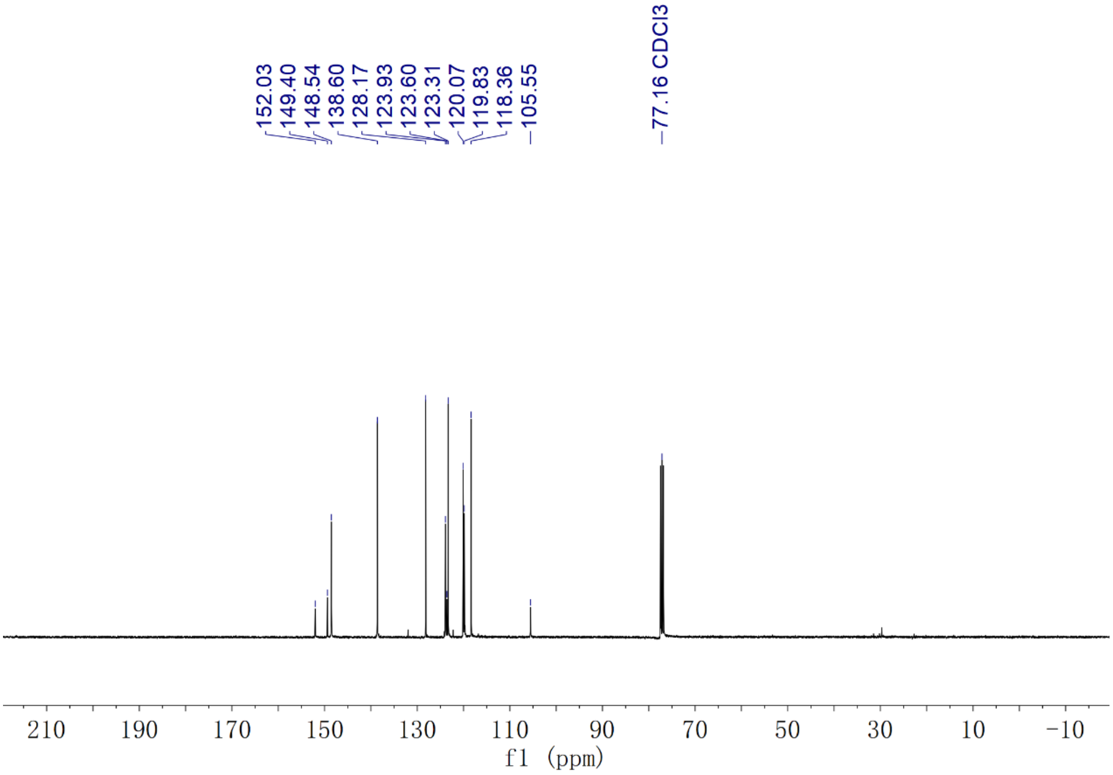

2s ¹H-NMR

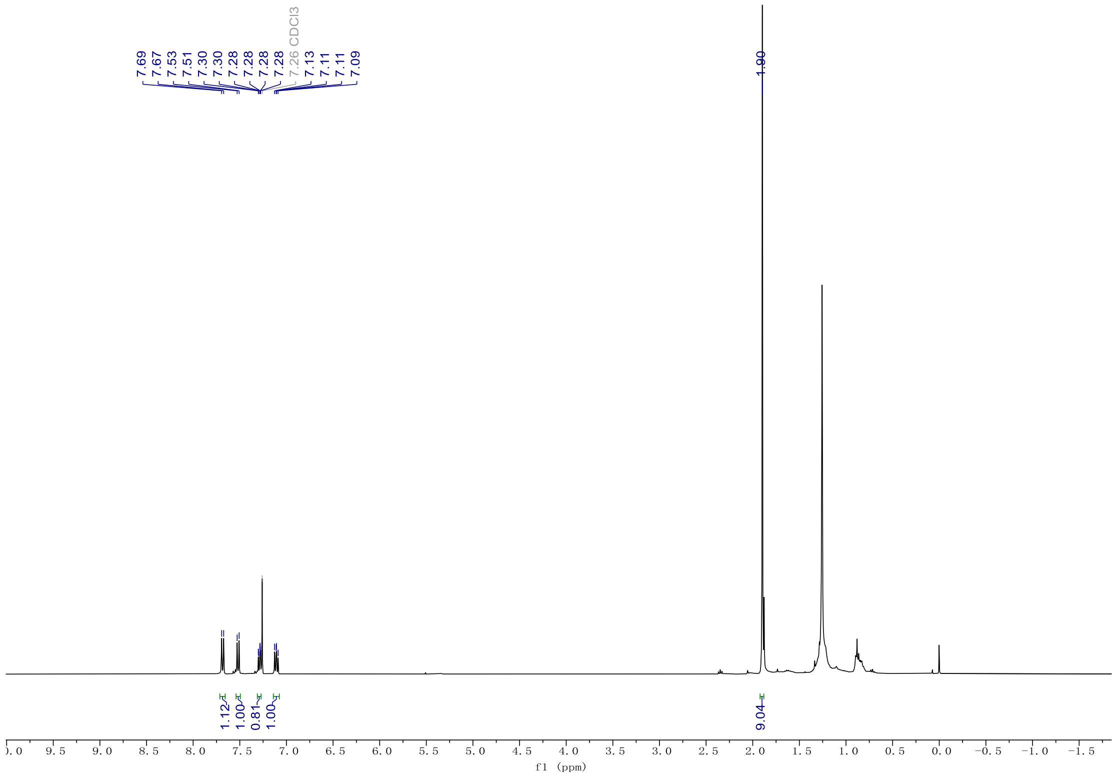

2s ¹³C-NMR

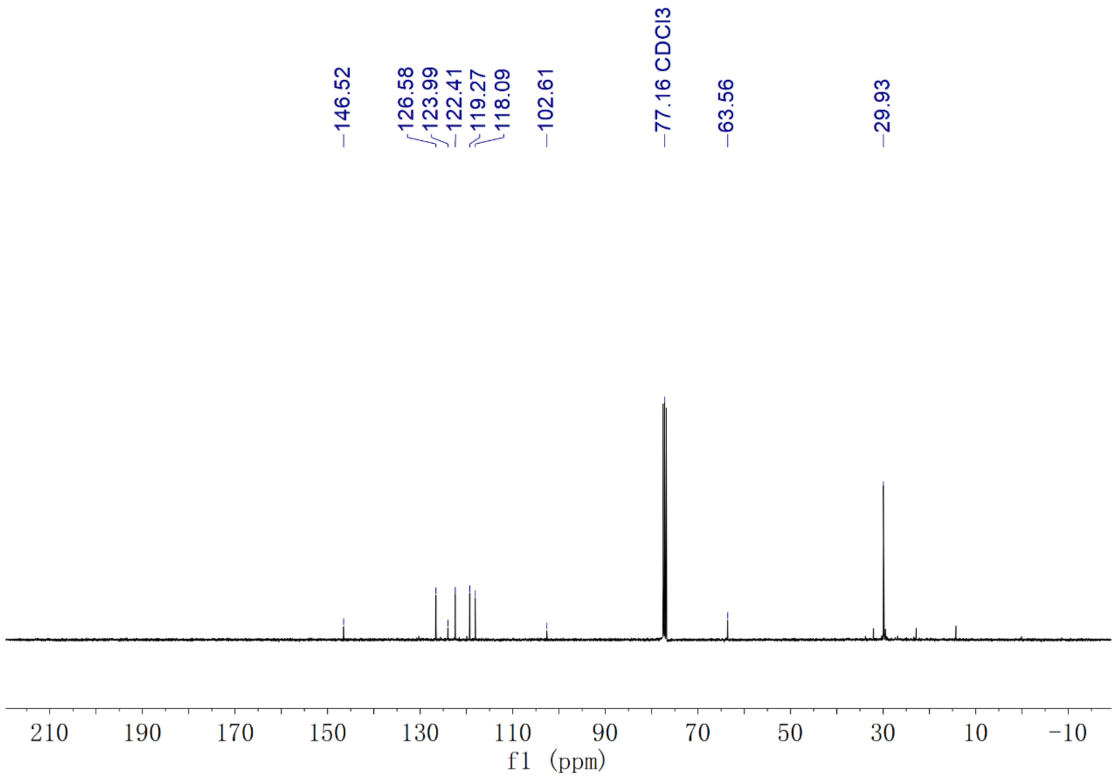

5a ^{1}H-NMR

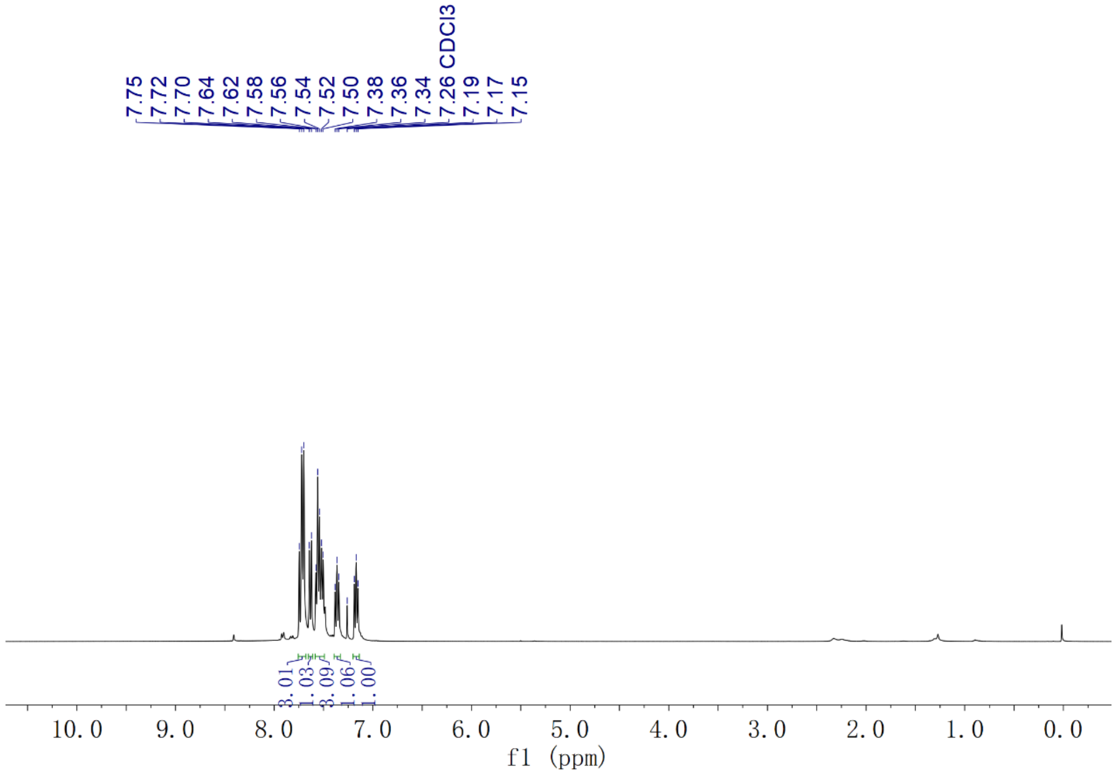

5a ^{13}C-NMR

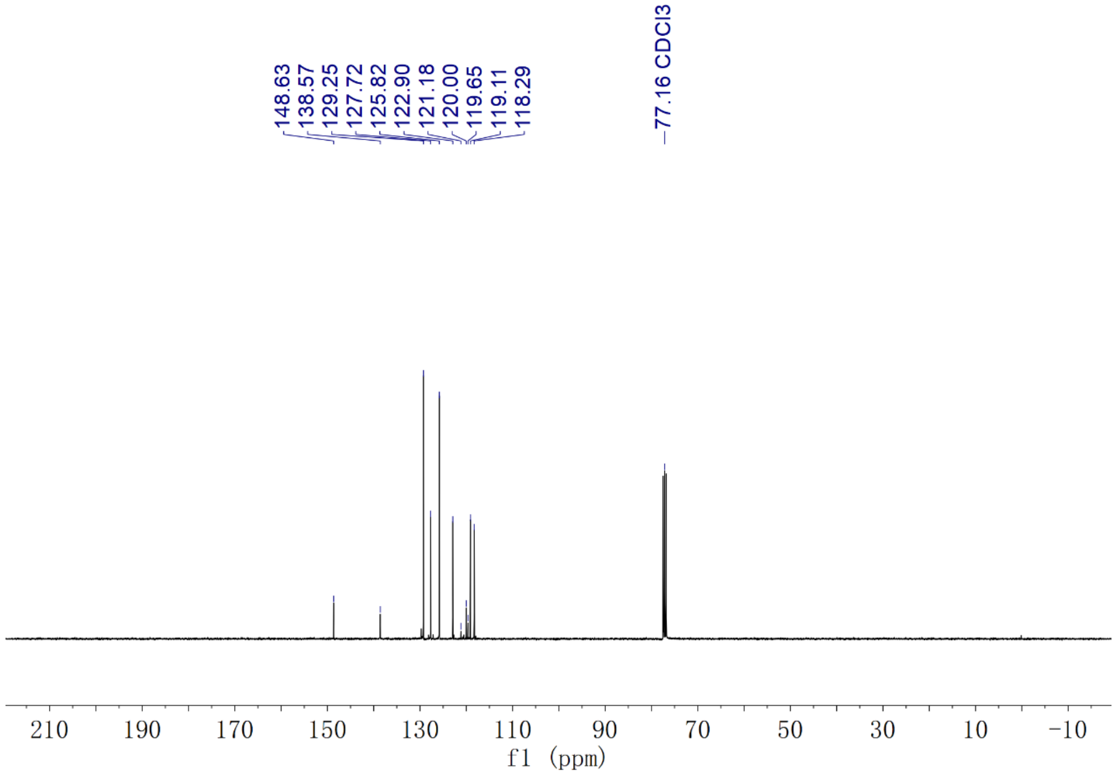

5b ^{1}H-NMR

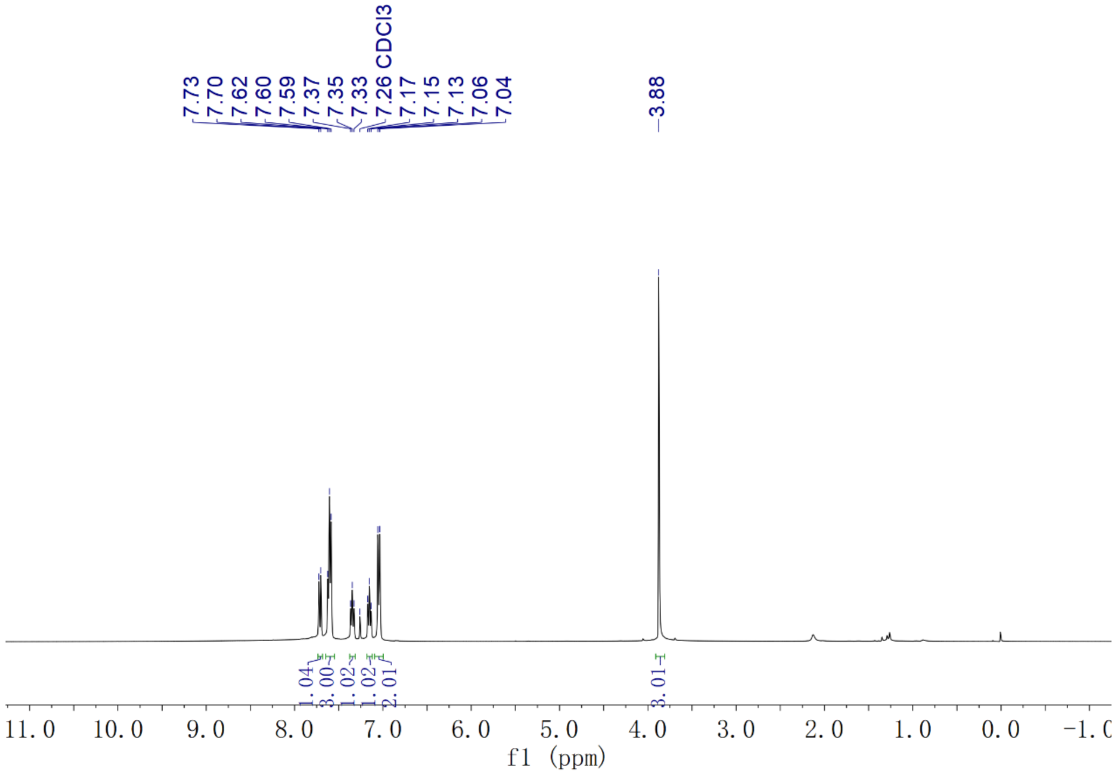

5b ^{13}C-NMR

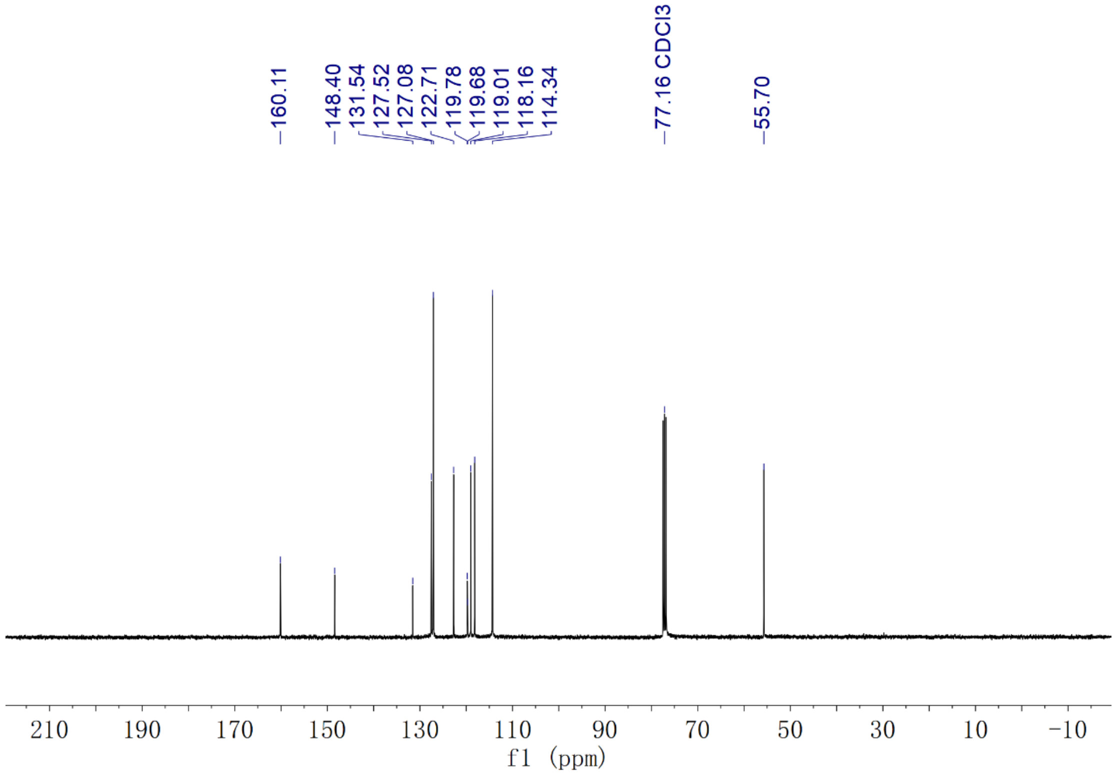

5c ^{1}H-NMR

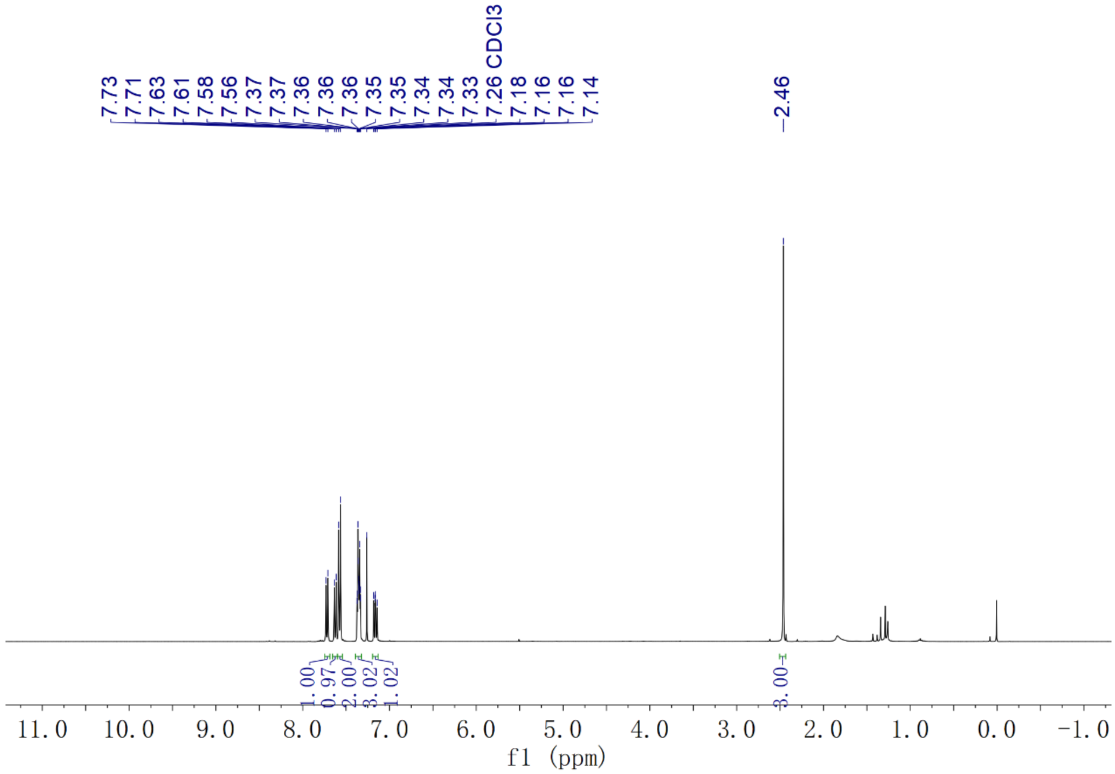

5c ^{13}C-NMR

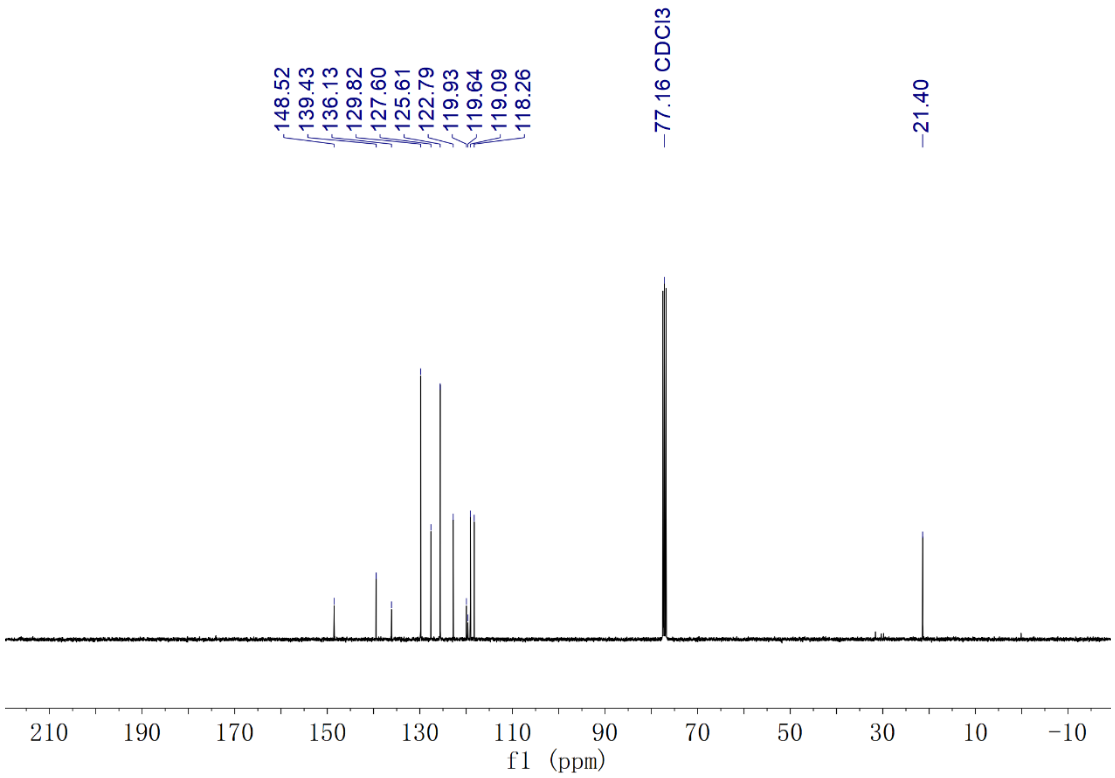

5d ^{1}H-NMR

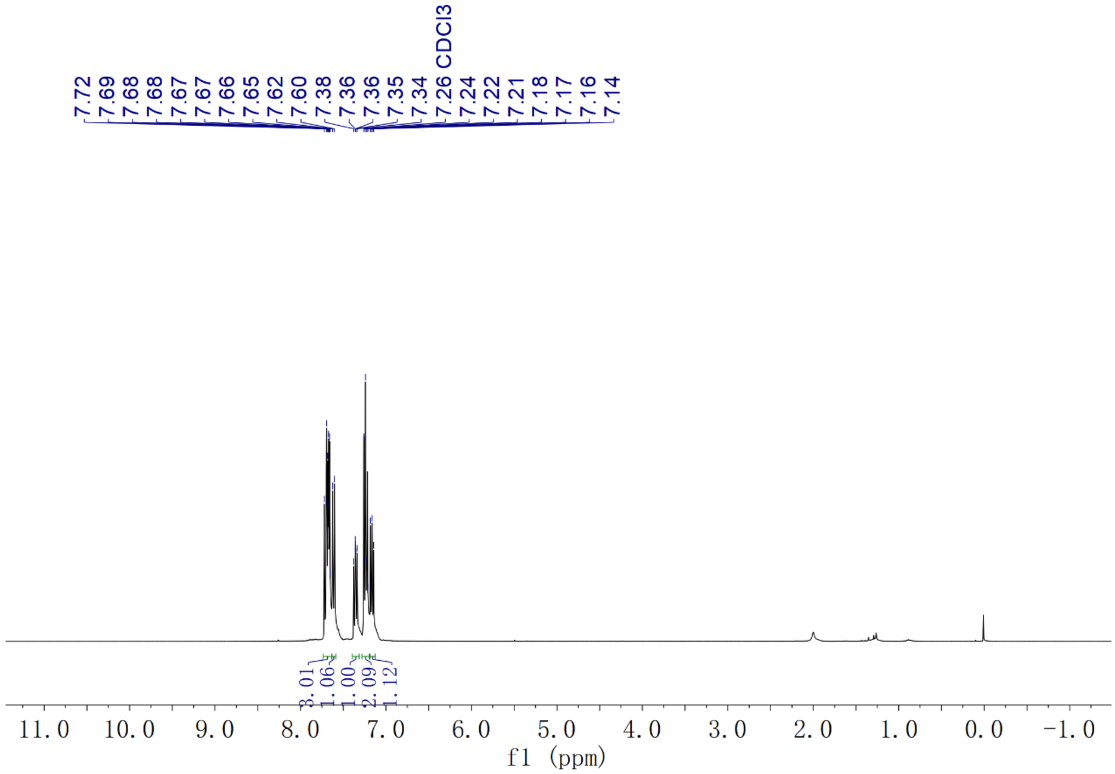

5c ^{13}C-NMR

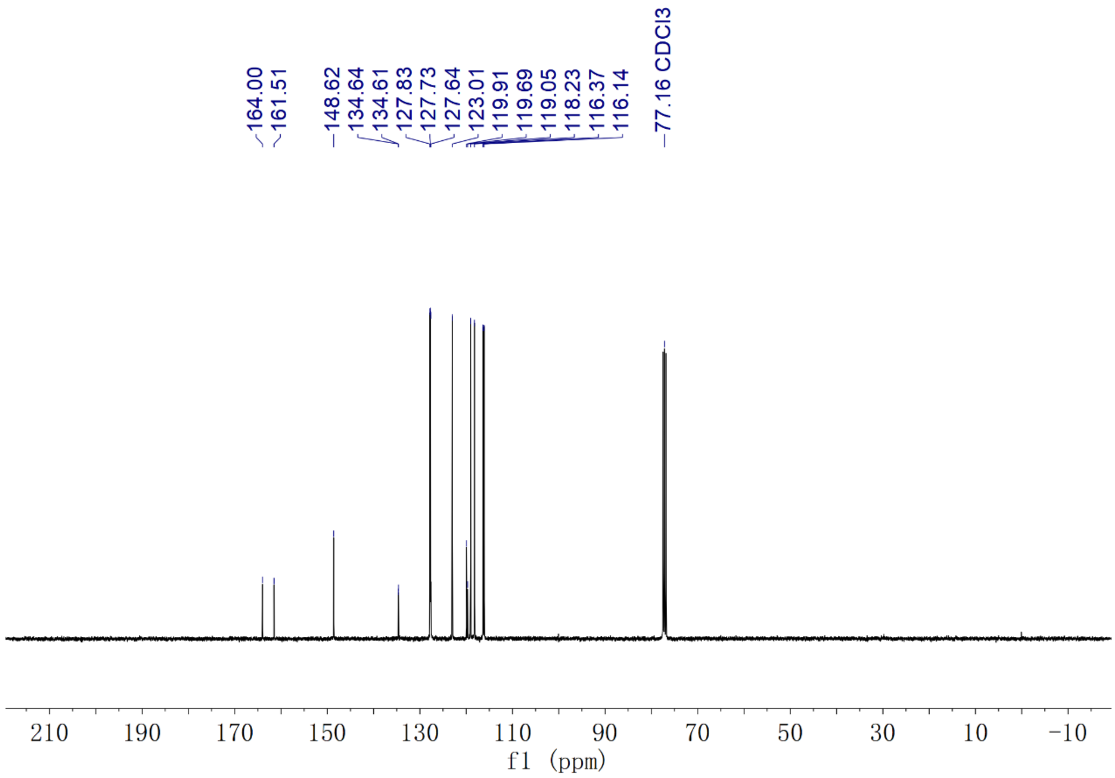

5c ^{19}F-NMR

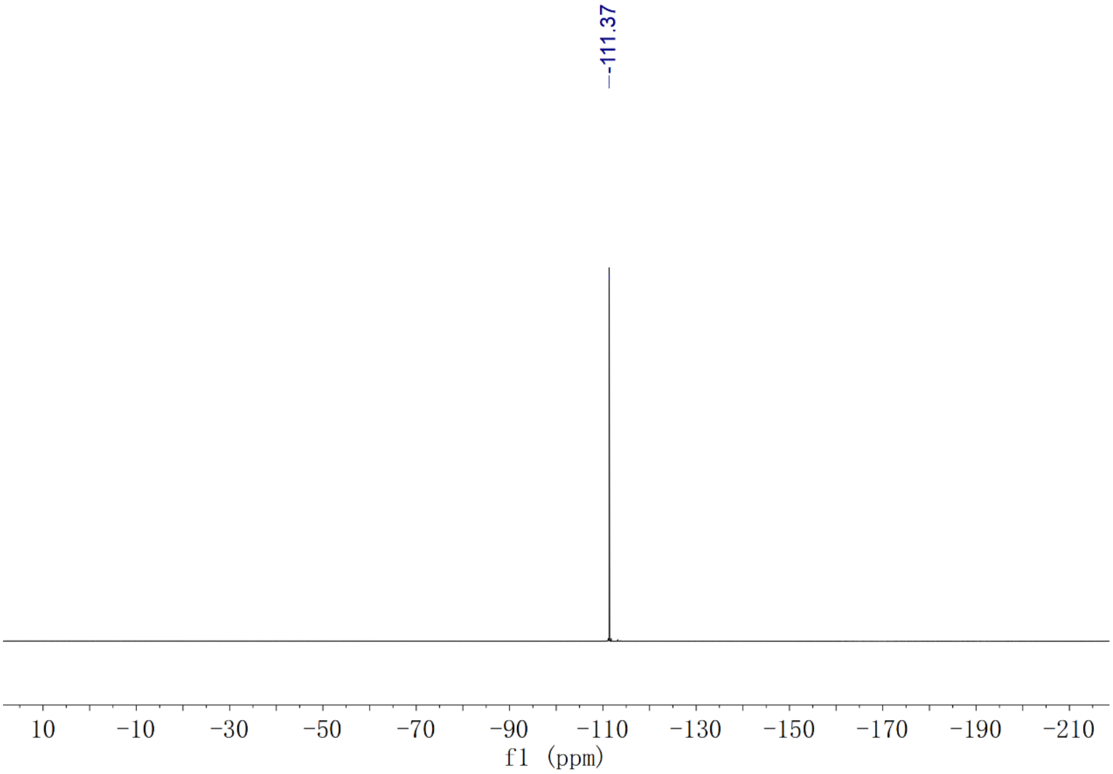

5e ^{1}H-NMR

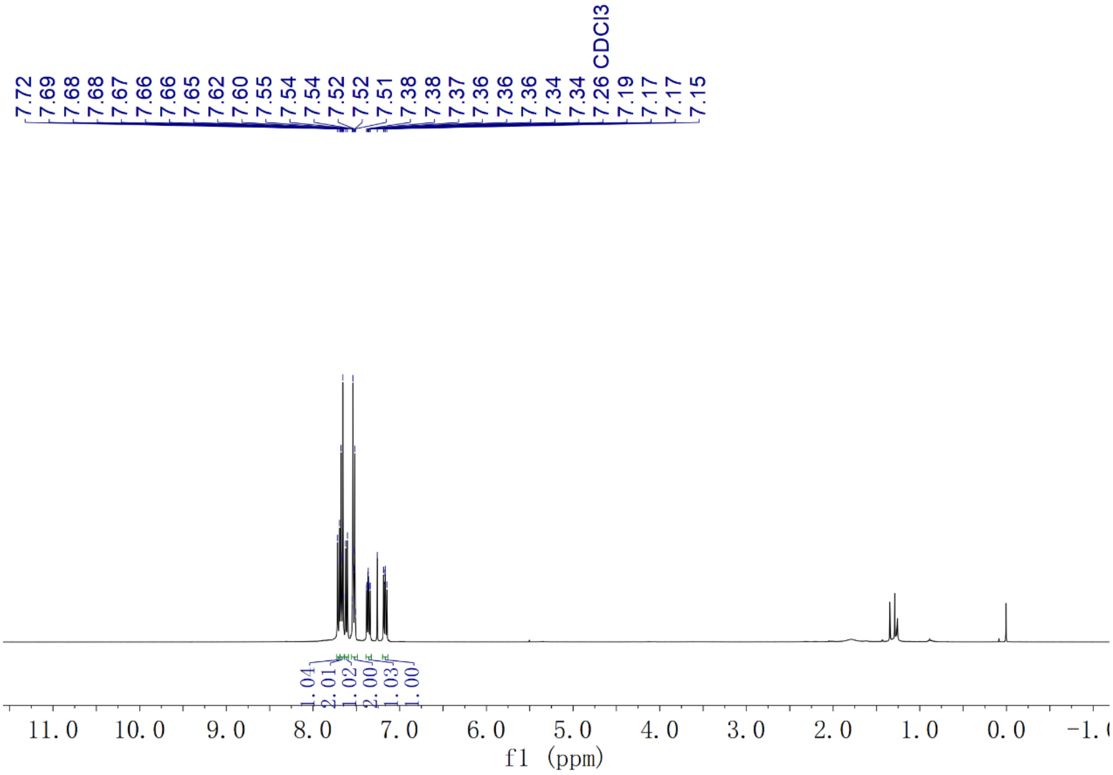

5e [1]C-NMR

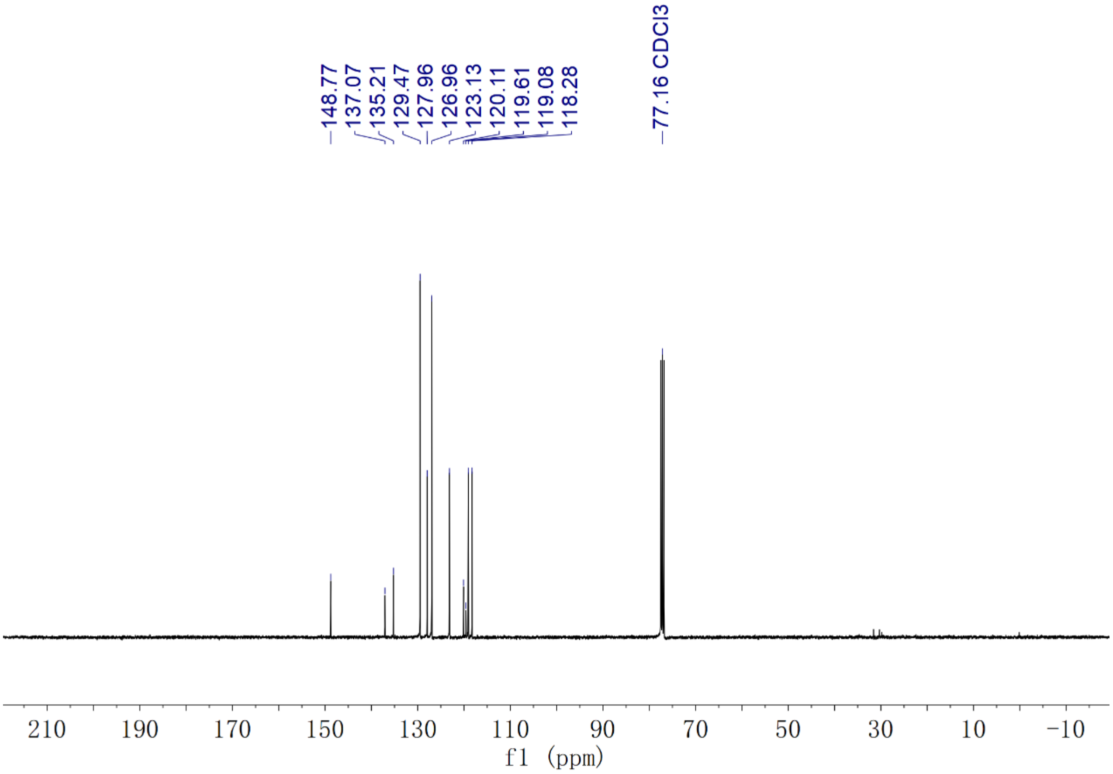

5f ^{1}H-NMR

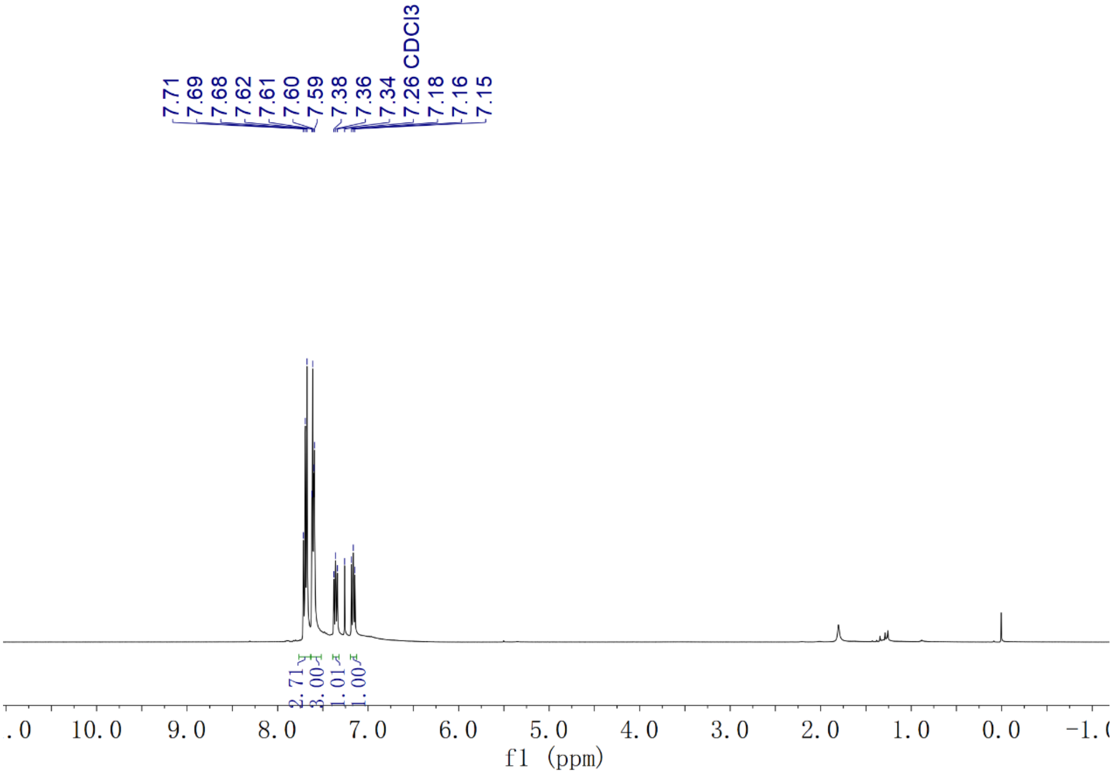

5f ^{13}C-NMR

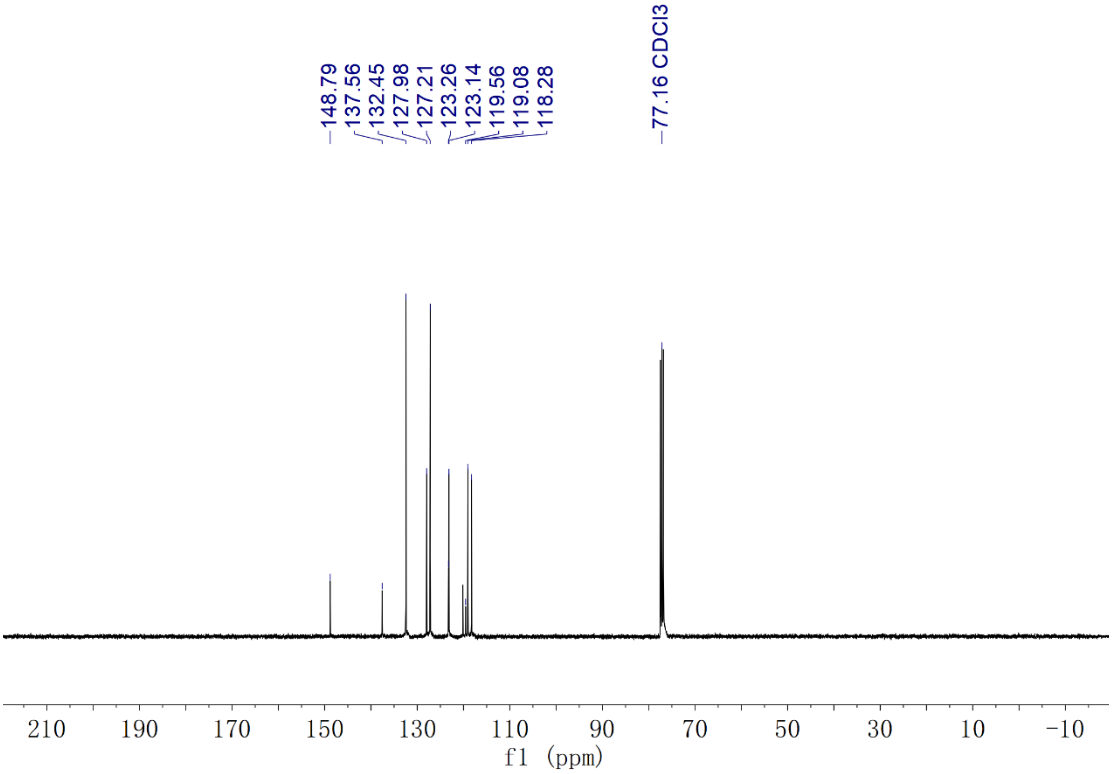

5g ^{1}H-NMR

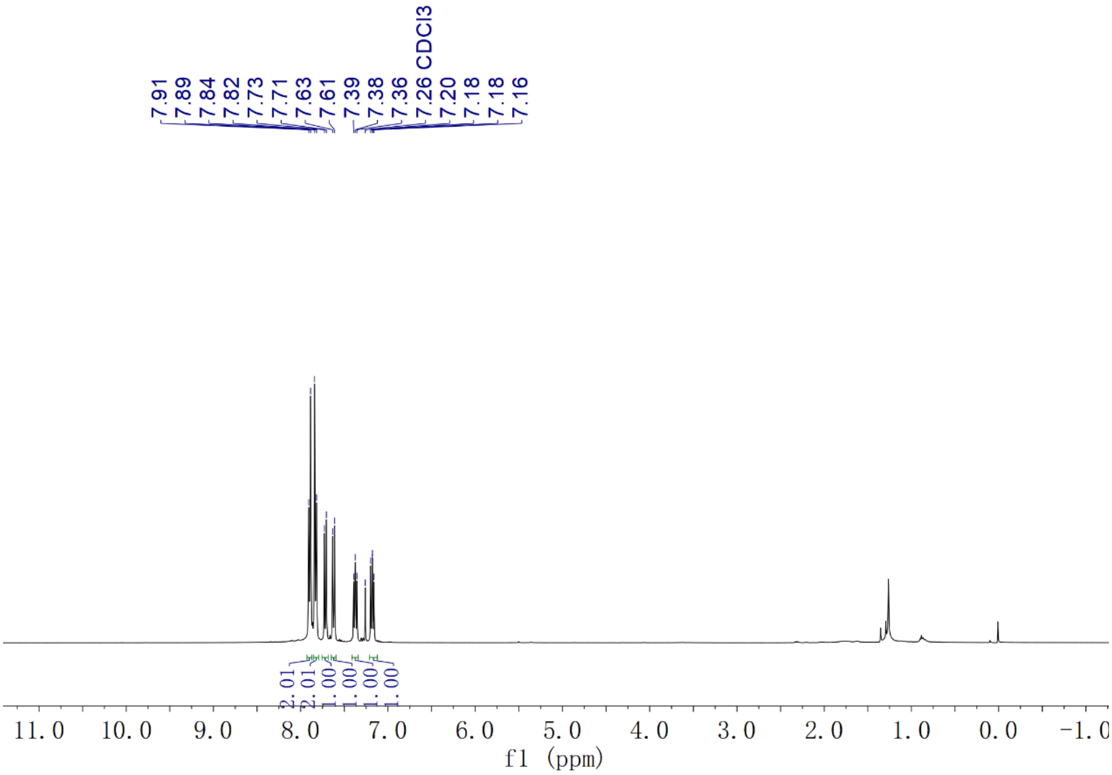

5g ^{13}C-NMR

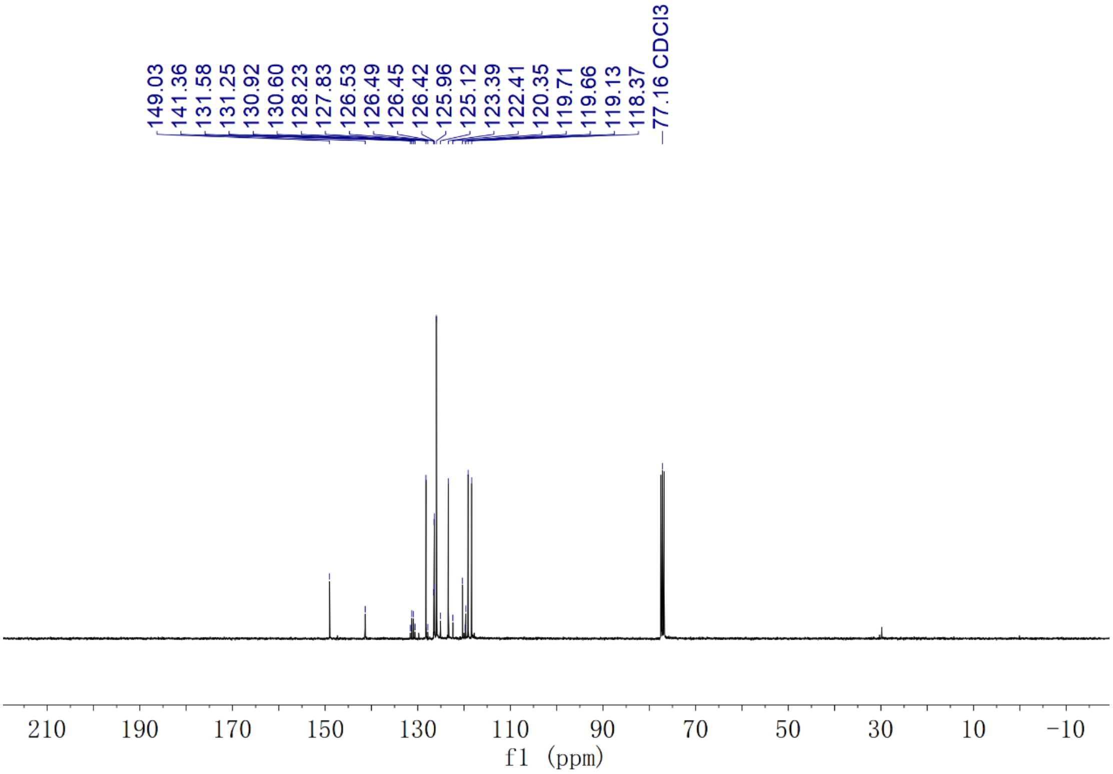

5g ^{19}F-NMR

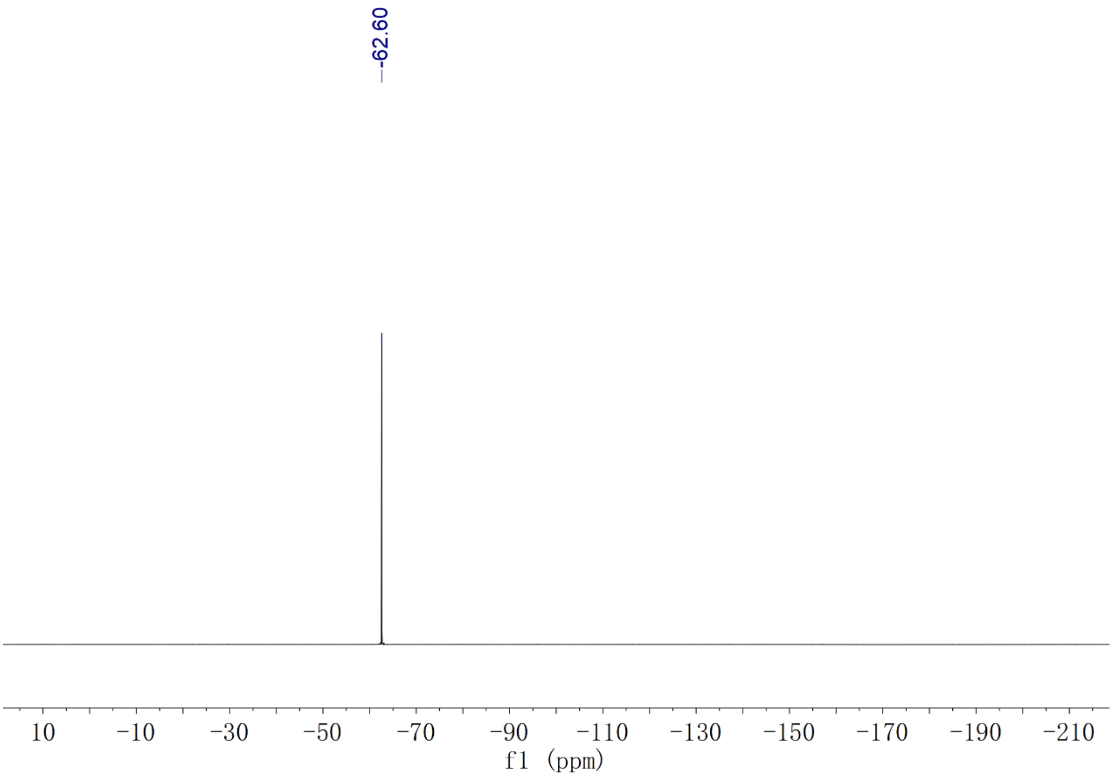

5h ¹H-NMR

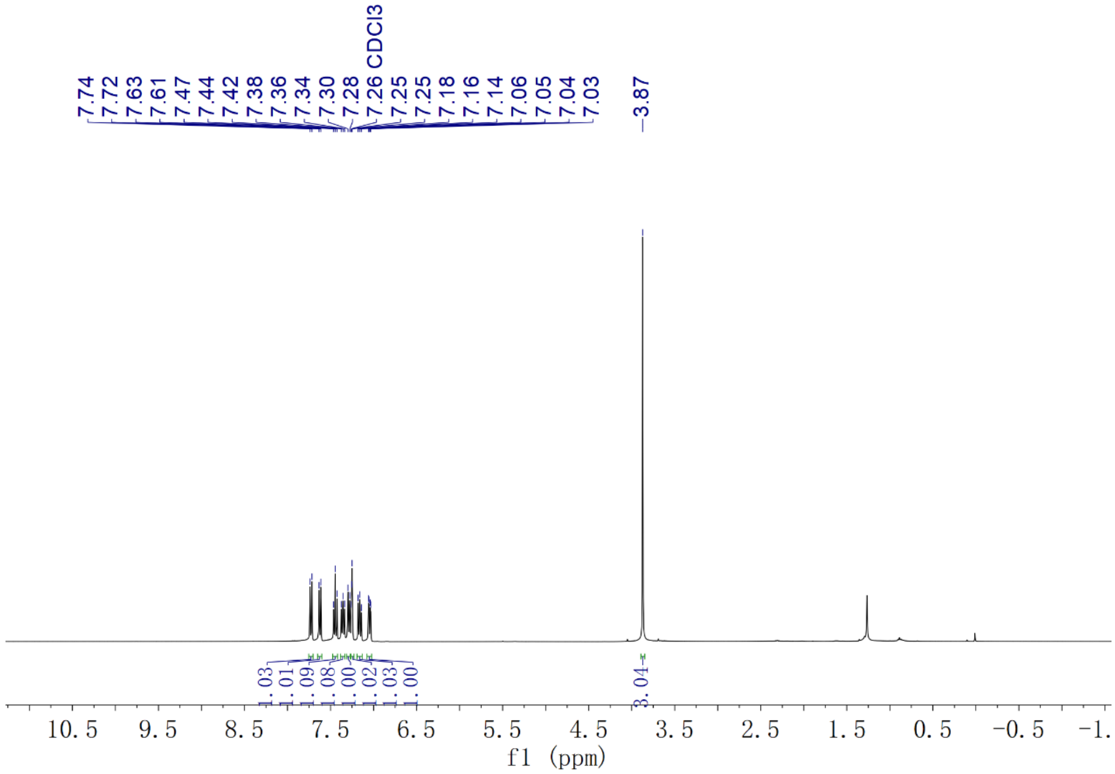

5h ¹³C-NMR

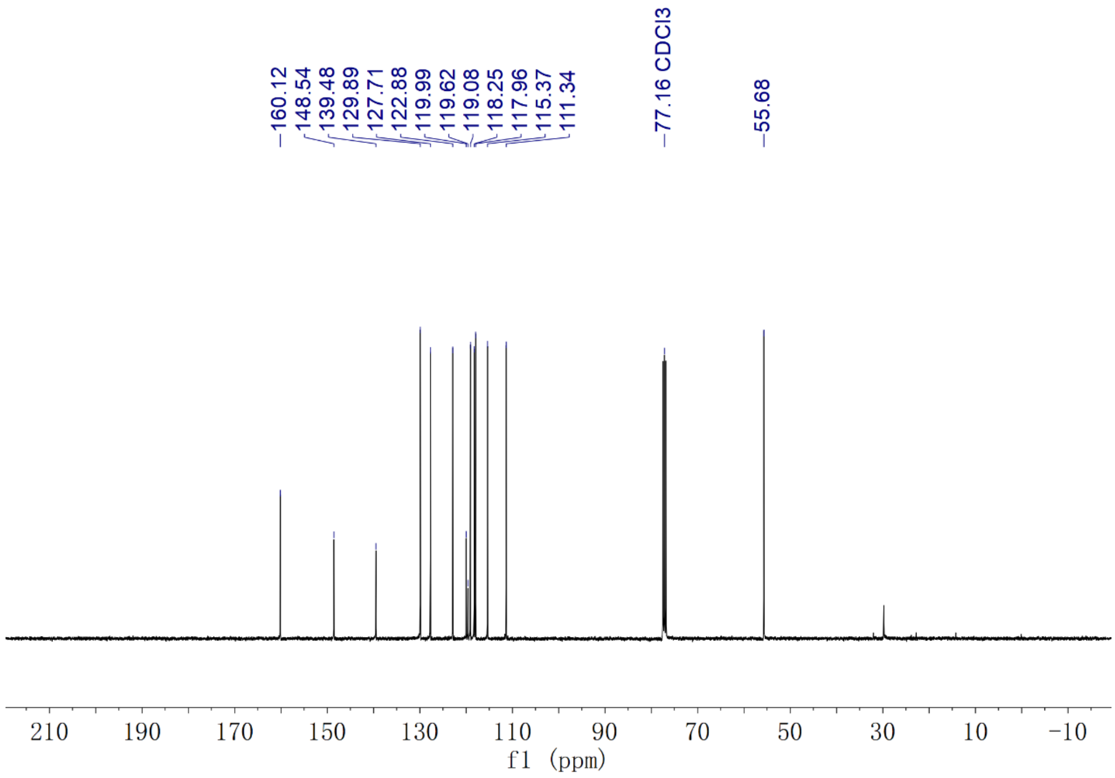

5i ^{1}H-NMR

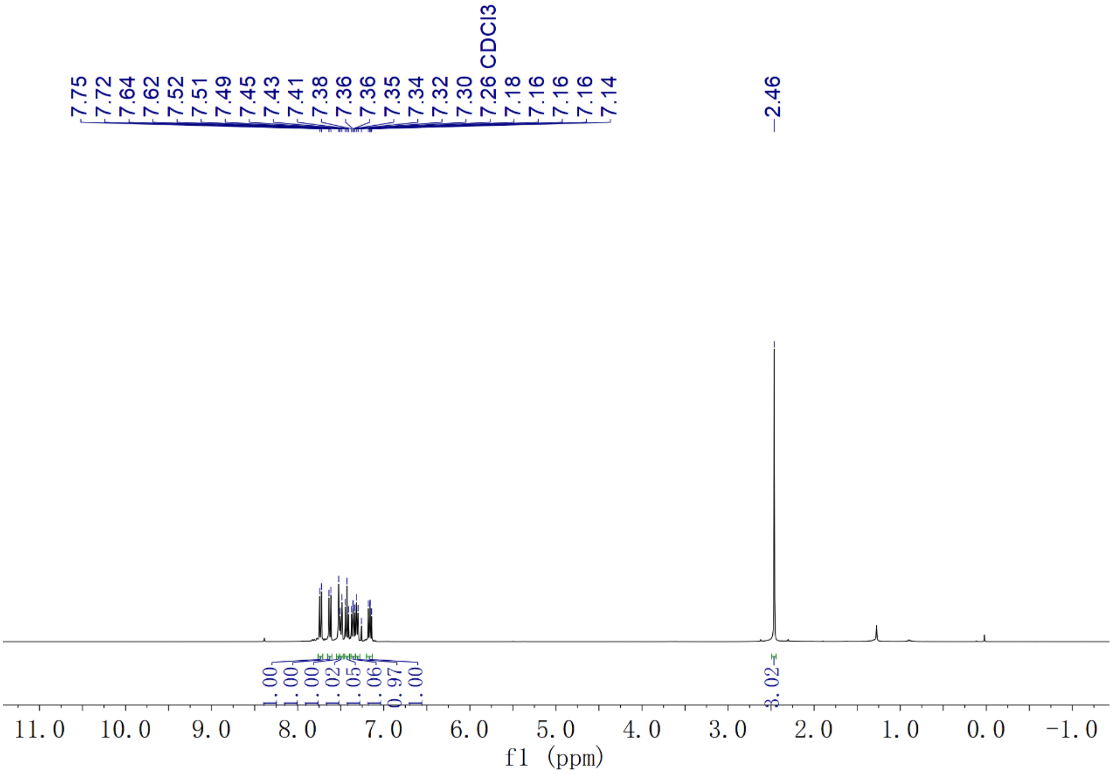

5i ^{13}C-NMR

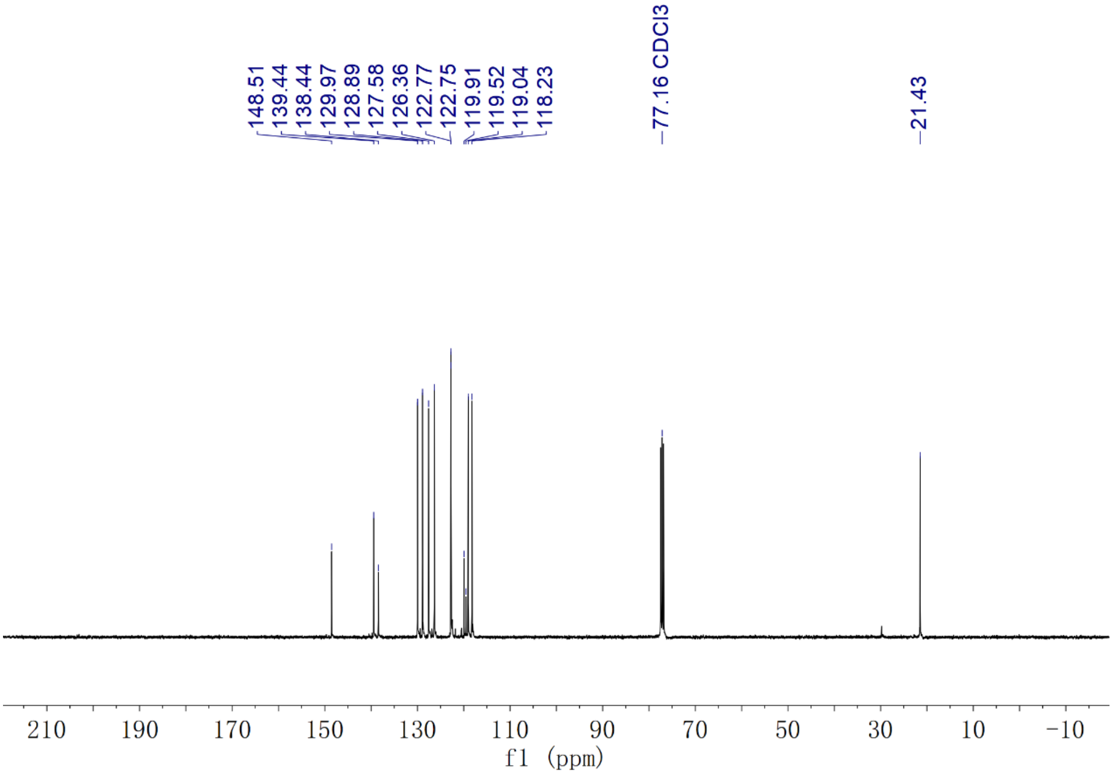

5j ^{1}H-NMR

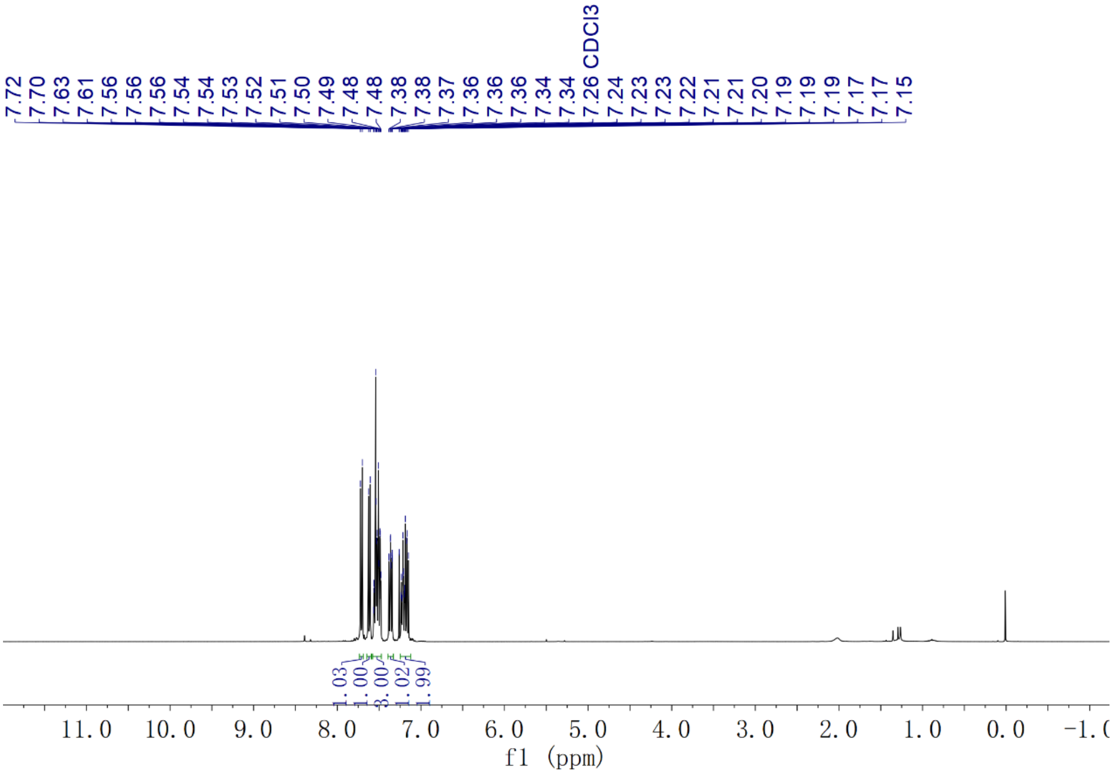

5j ^{13}C-NMR

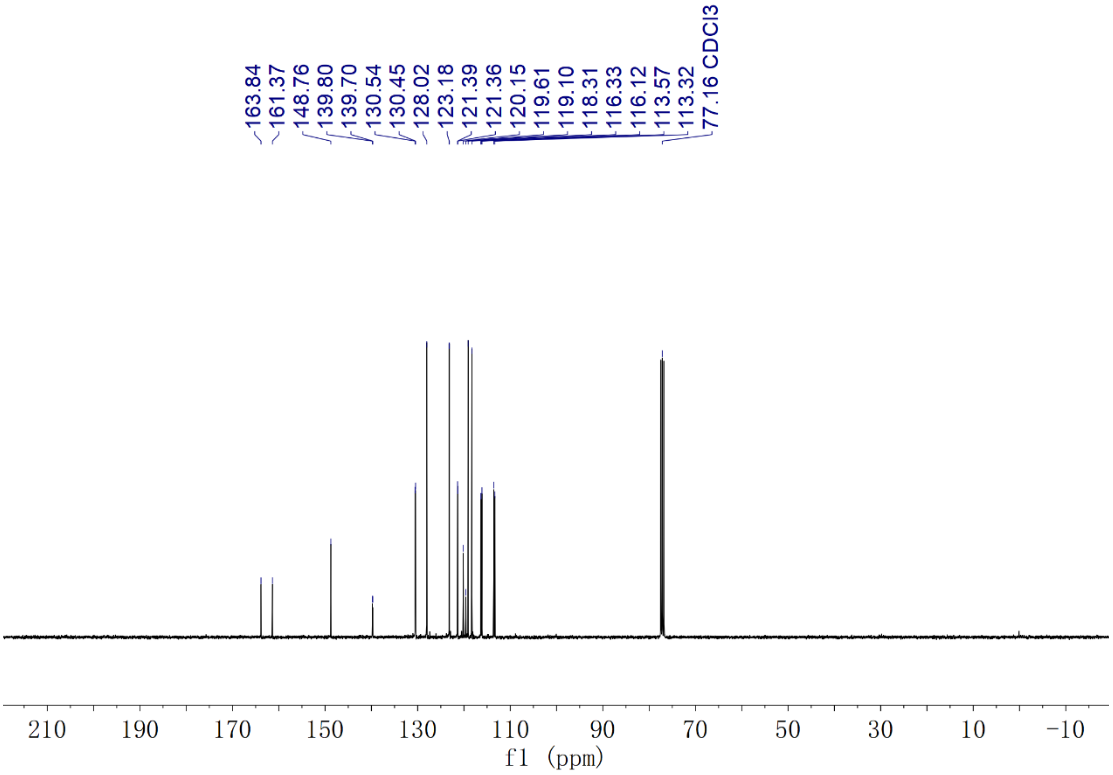

5j ^{19}F-NMR

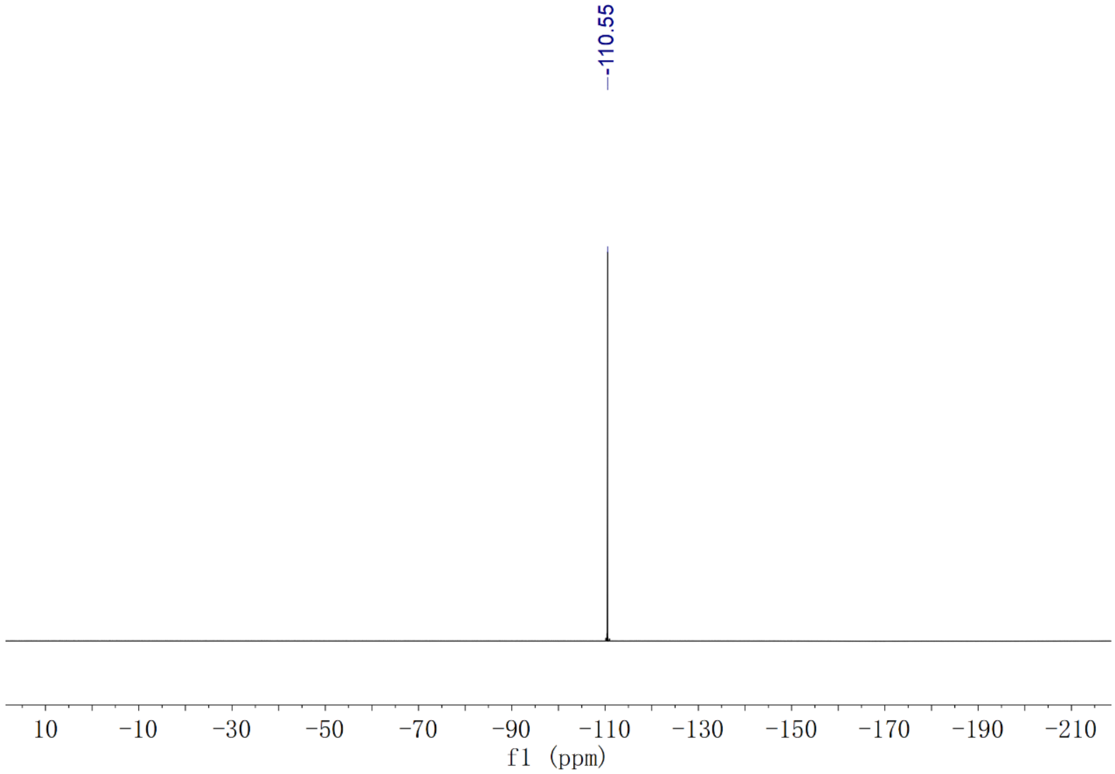

5k ^{1}H-NMR

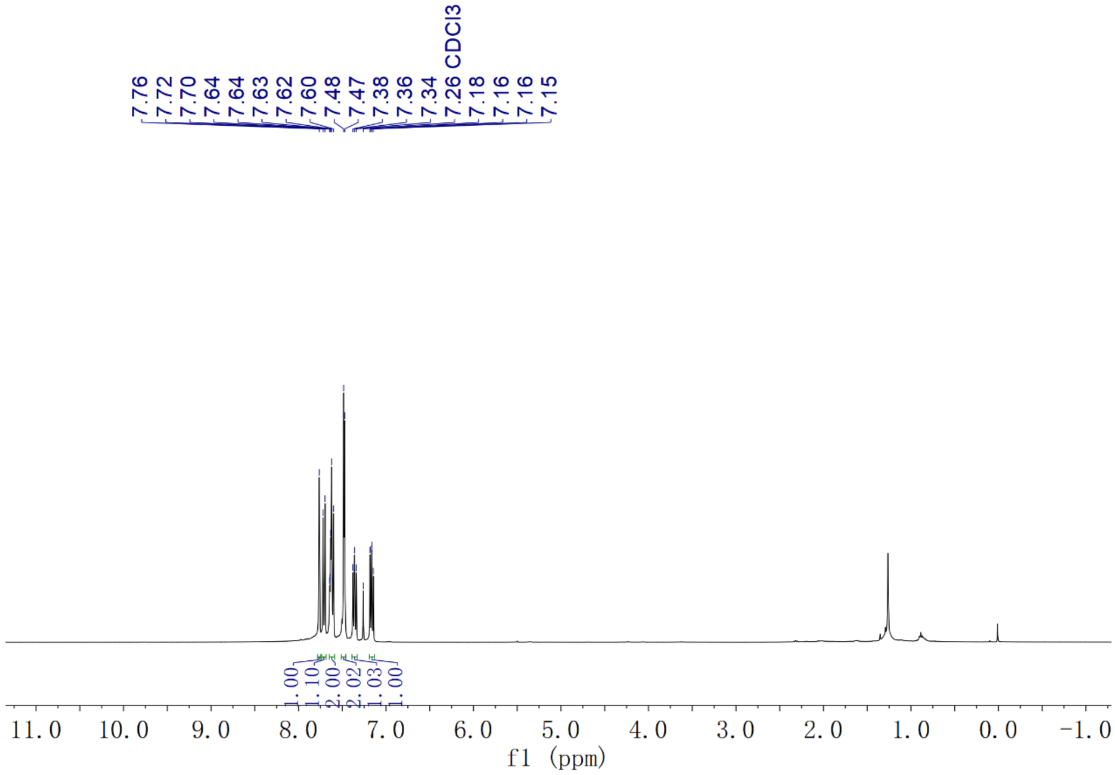

5k ^{13}C-NMR

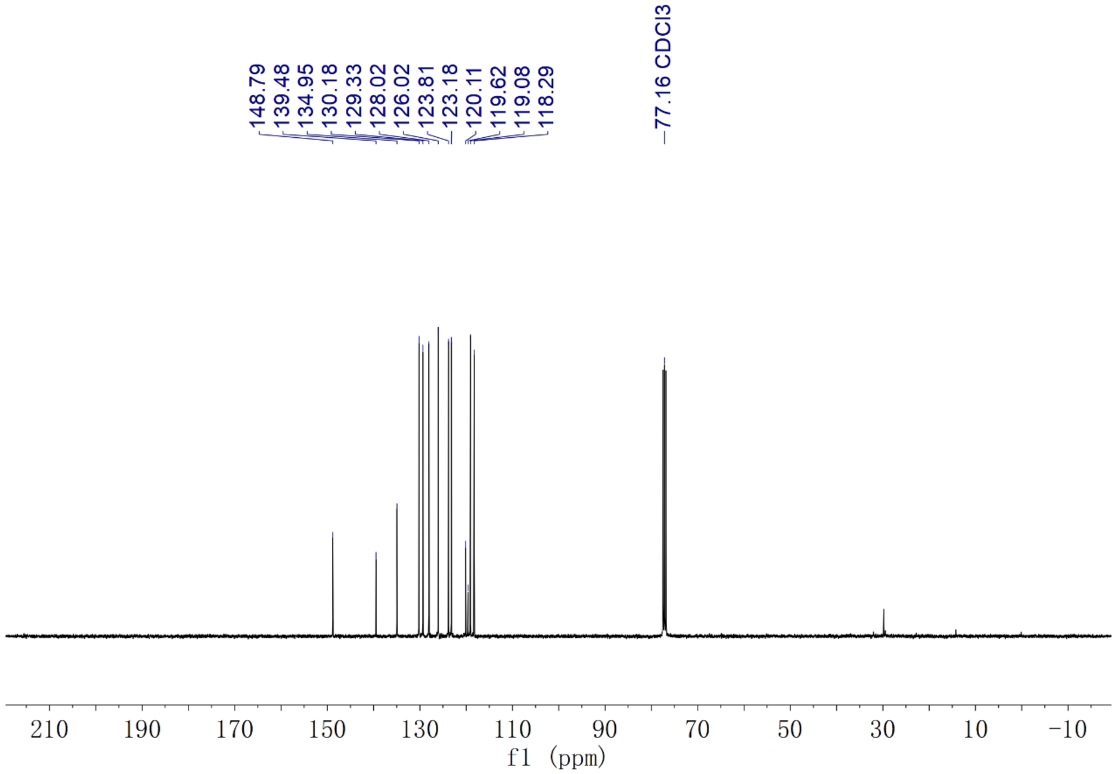

5l ^{1}H-NMR

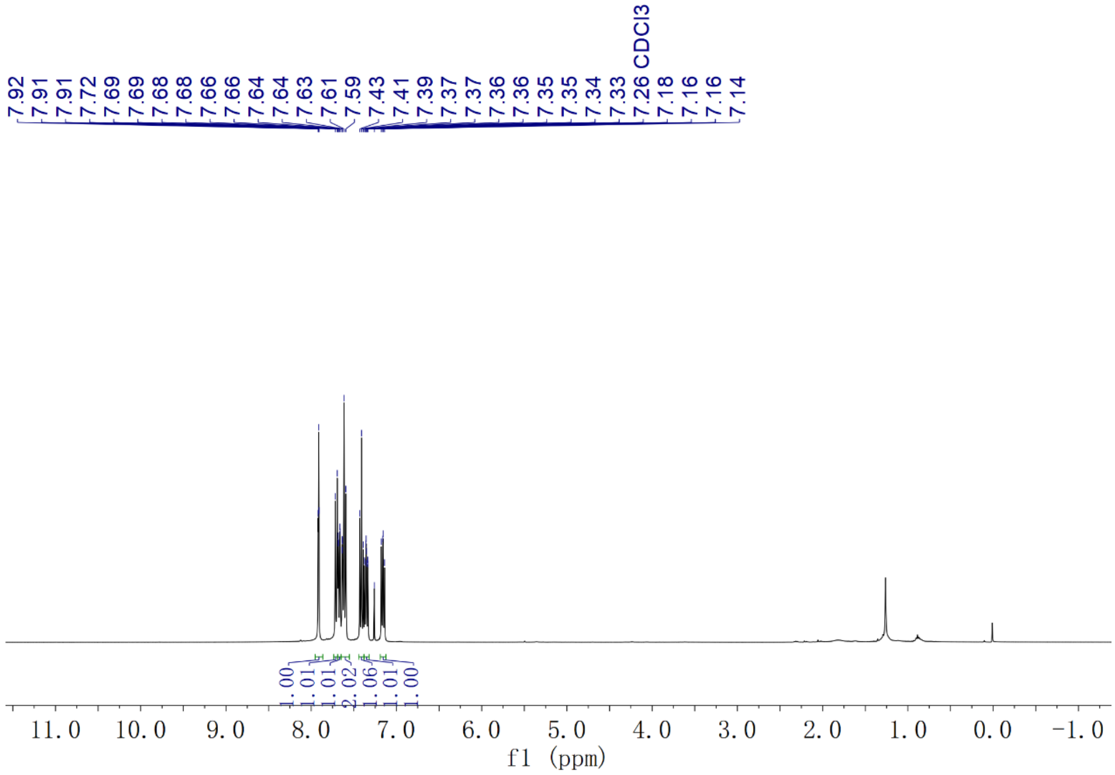

5l ^{13}C-NMR

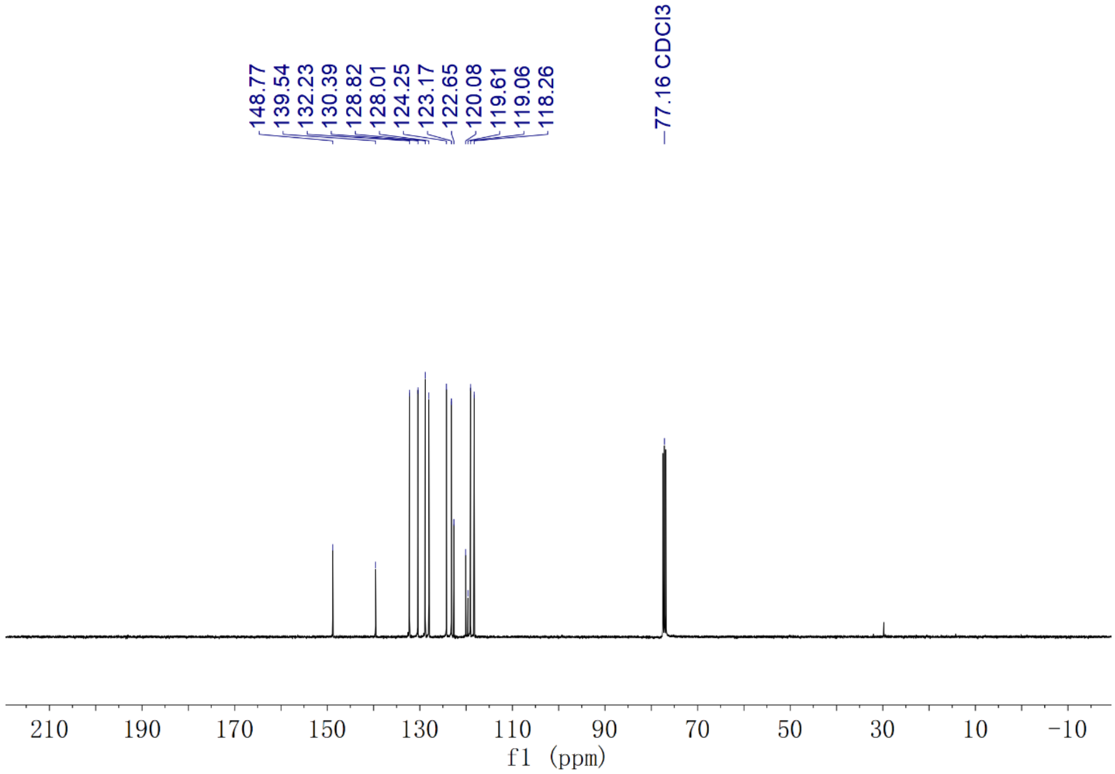

5m ^{1}H-NMR

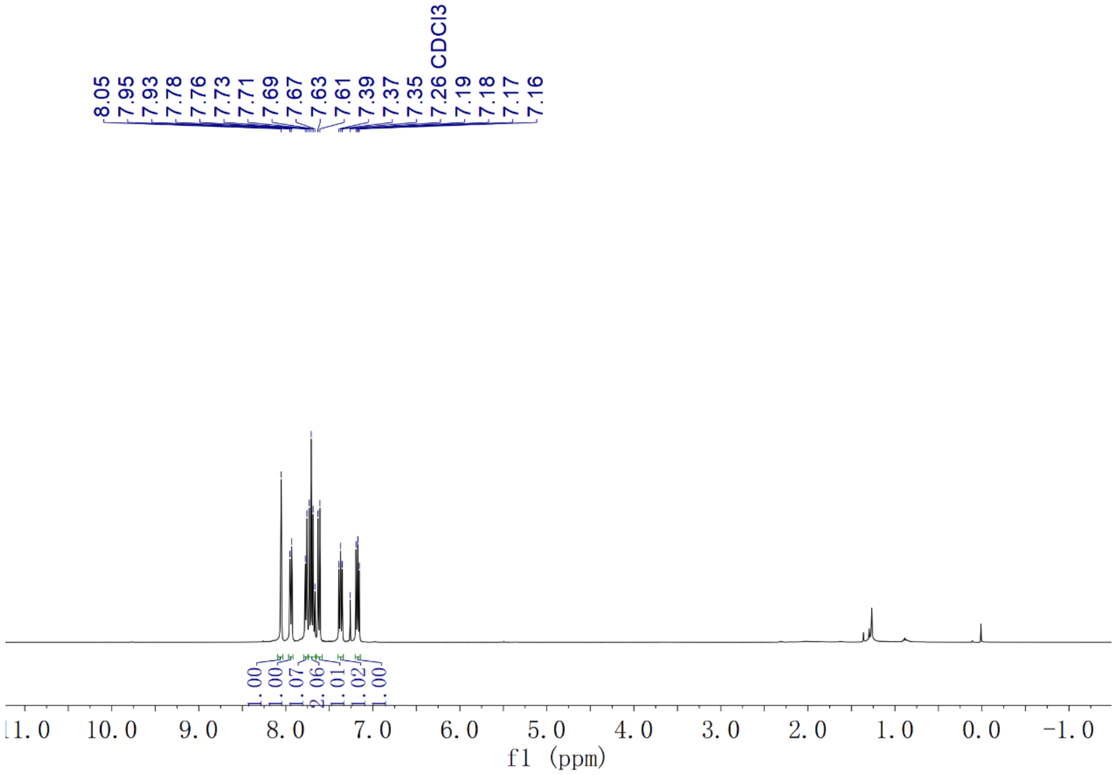

5m ^{1}H-NMR

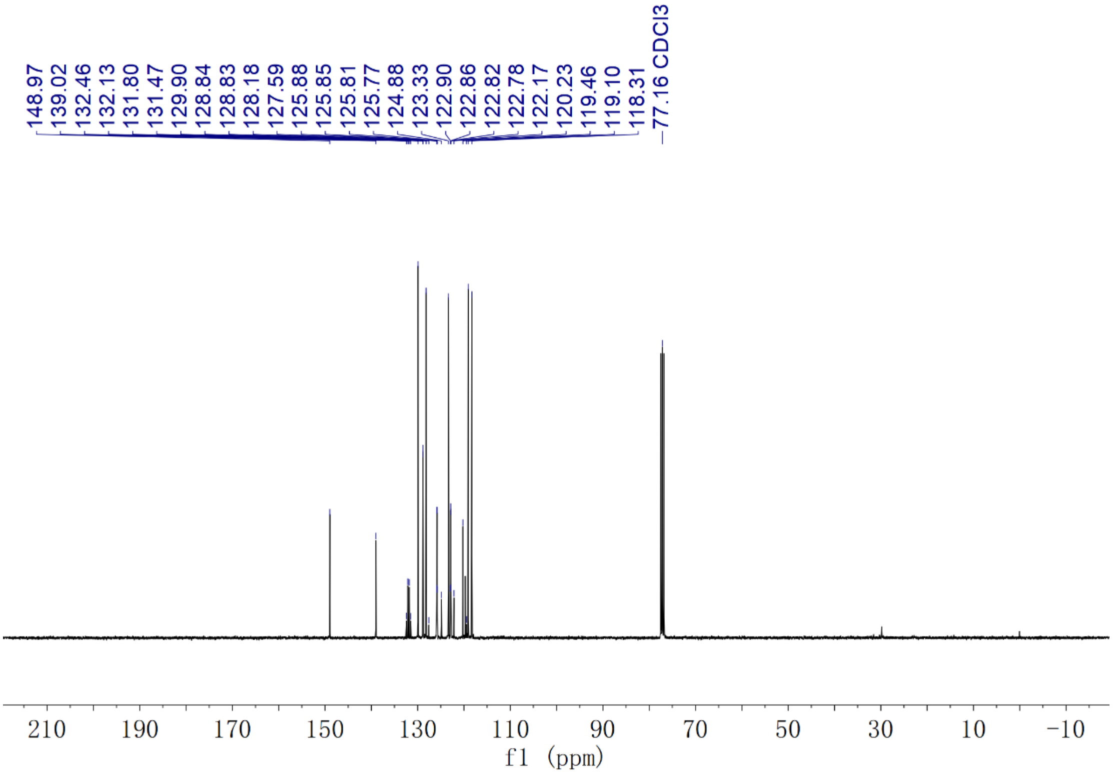

5m ^{19}F-NMR

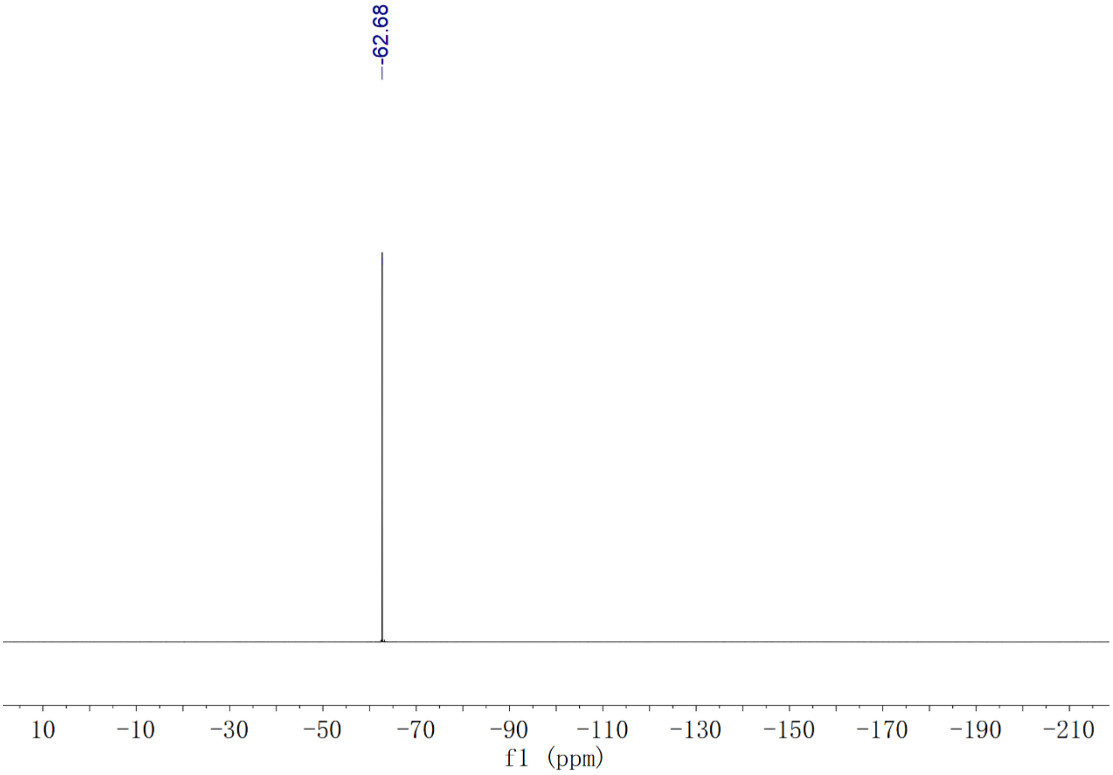

5n ^{1}H-NMR

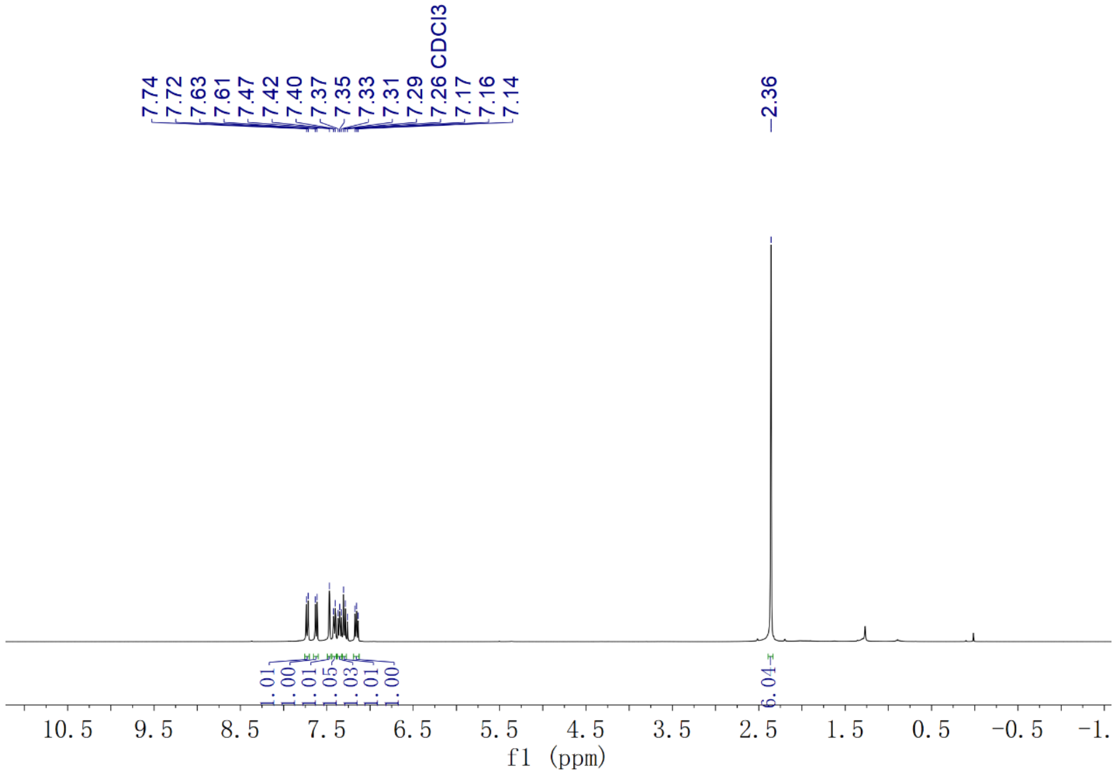

5n ^{13}C-NMR

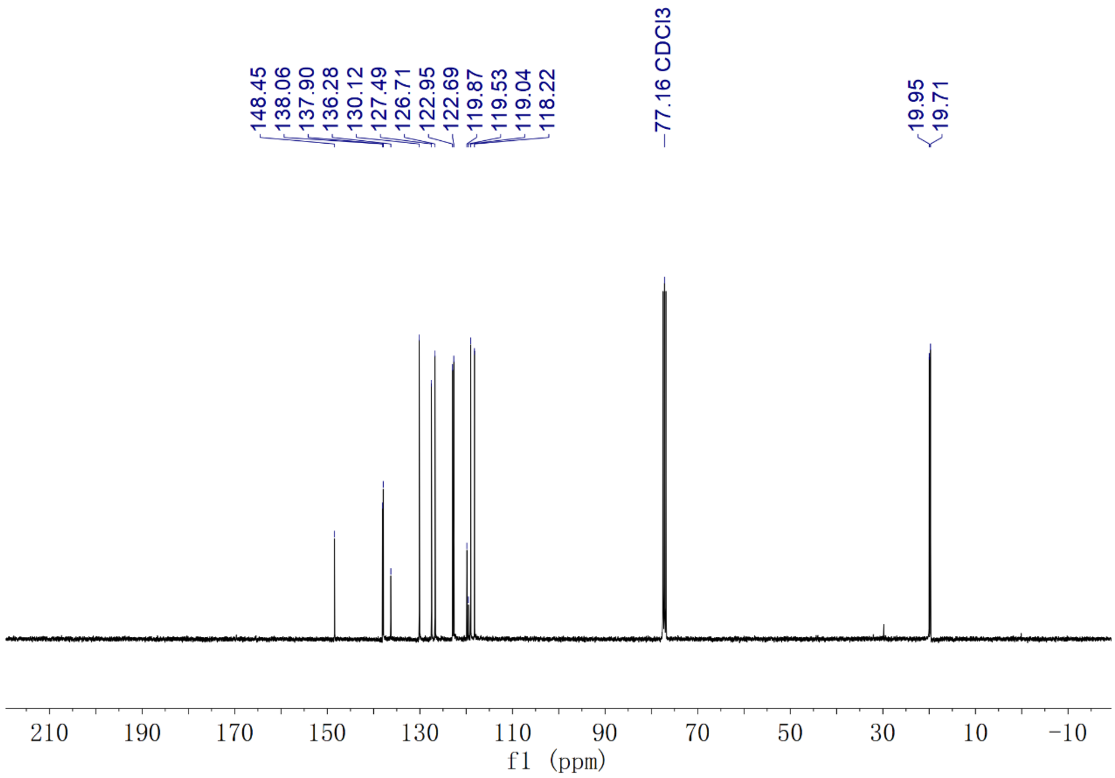

5o ^{1}H-NMR

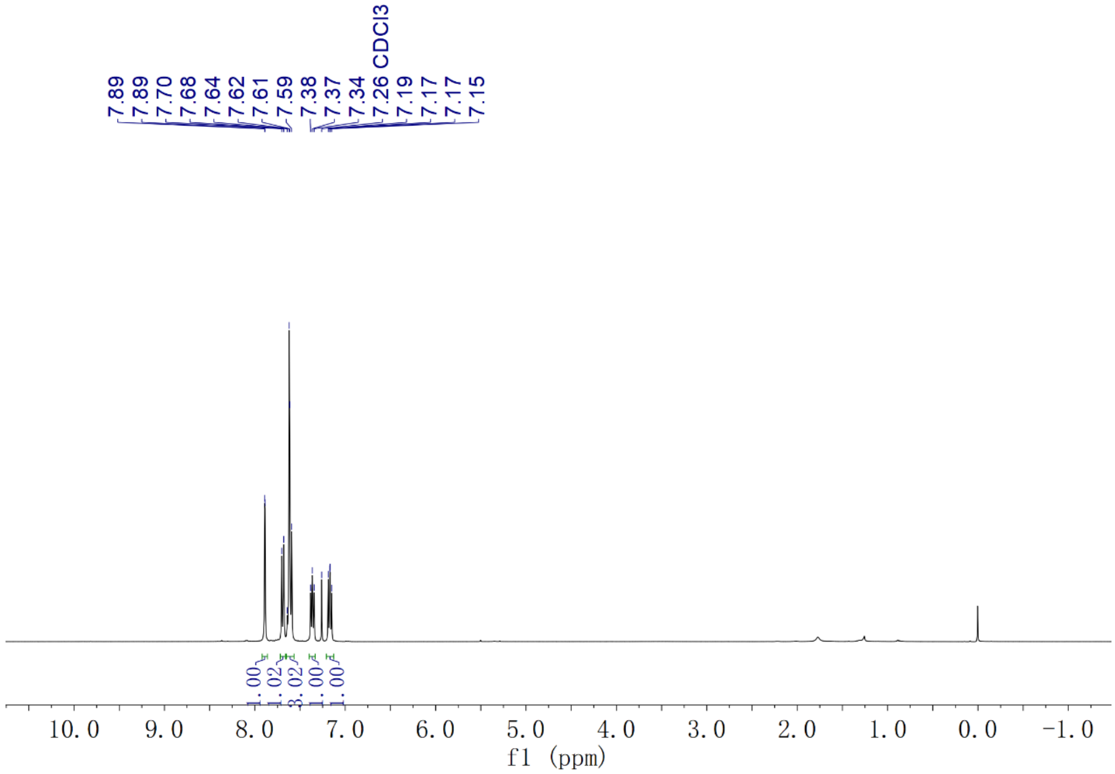

5o ^{13}C-NMR

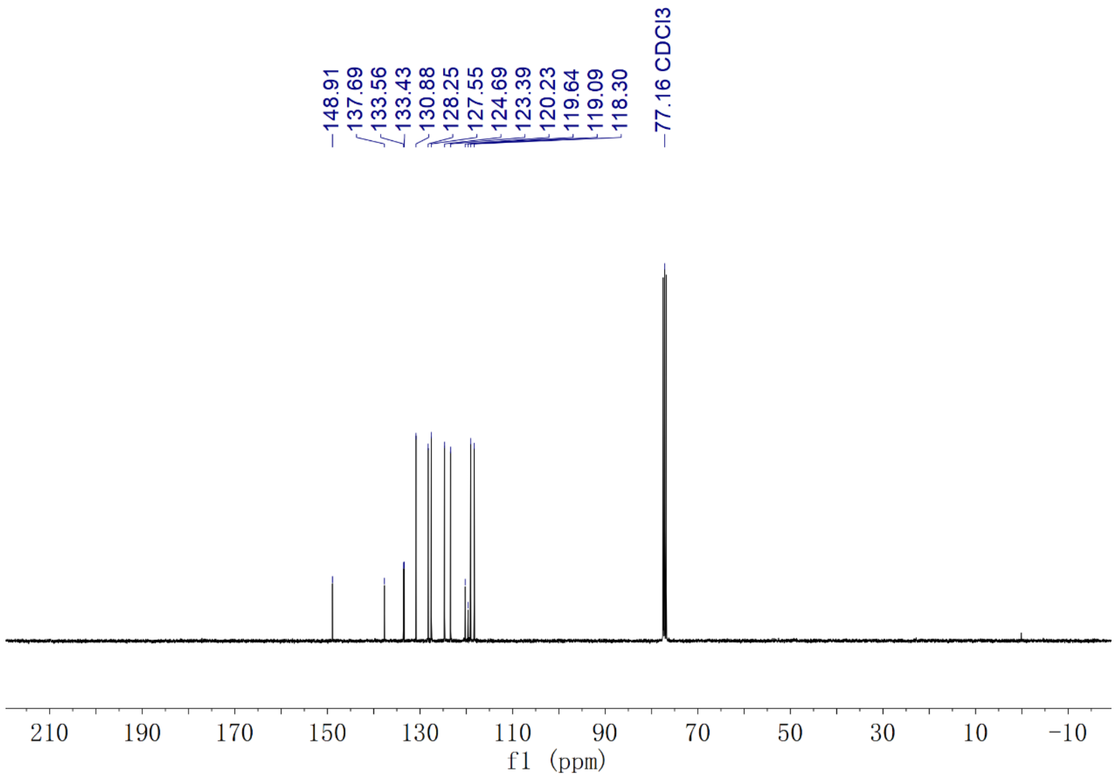

5p ¹H-NMR

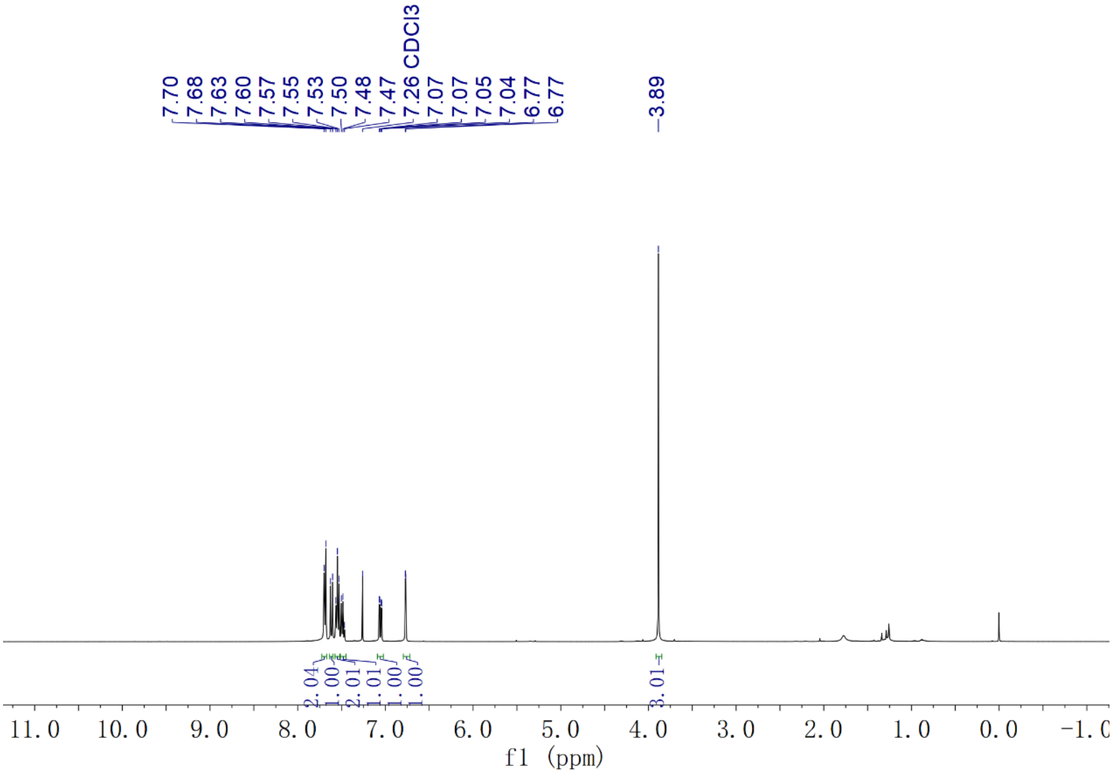

5p ¹³C-NMR

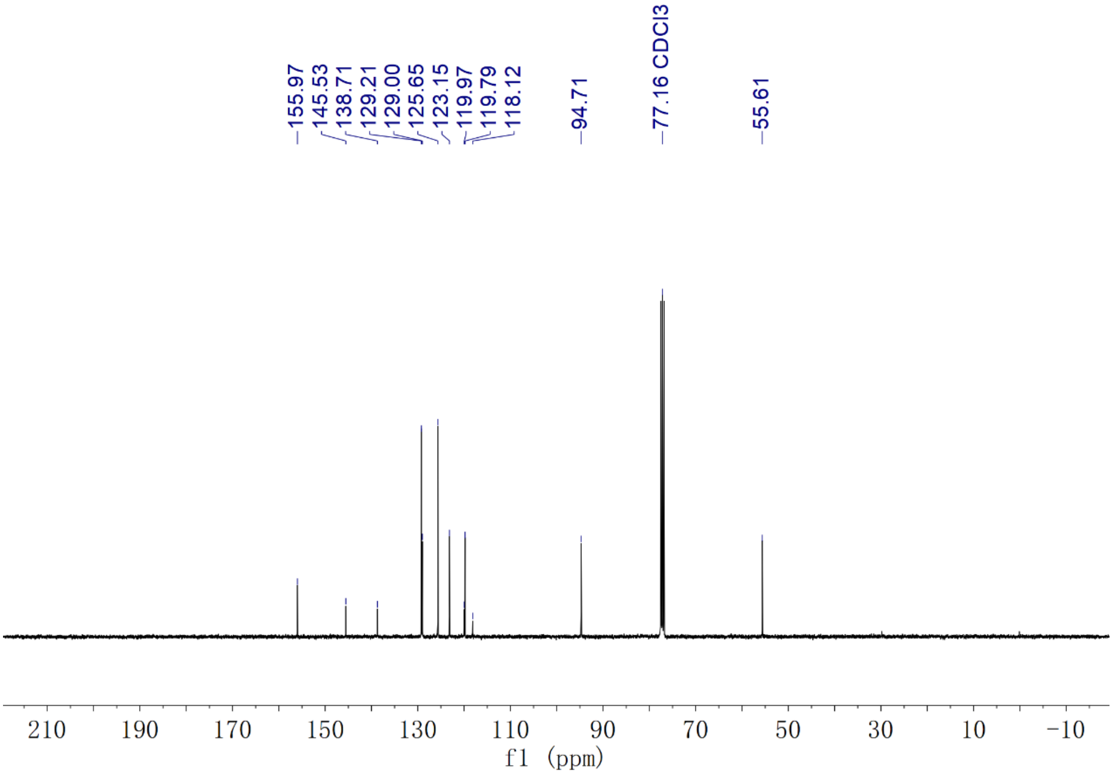

5q ^{1}H-NMR

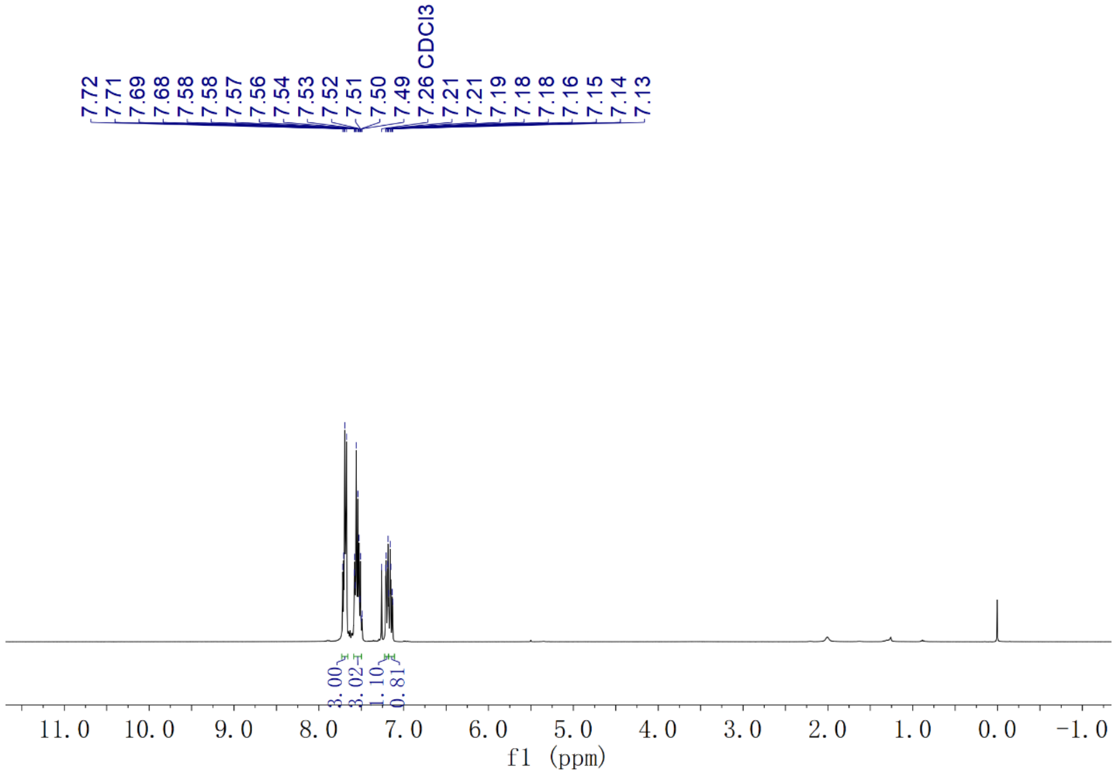

5q ^{13}C-NMR

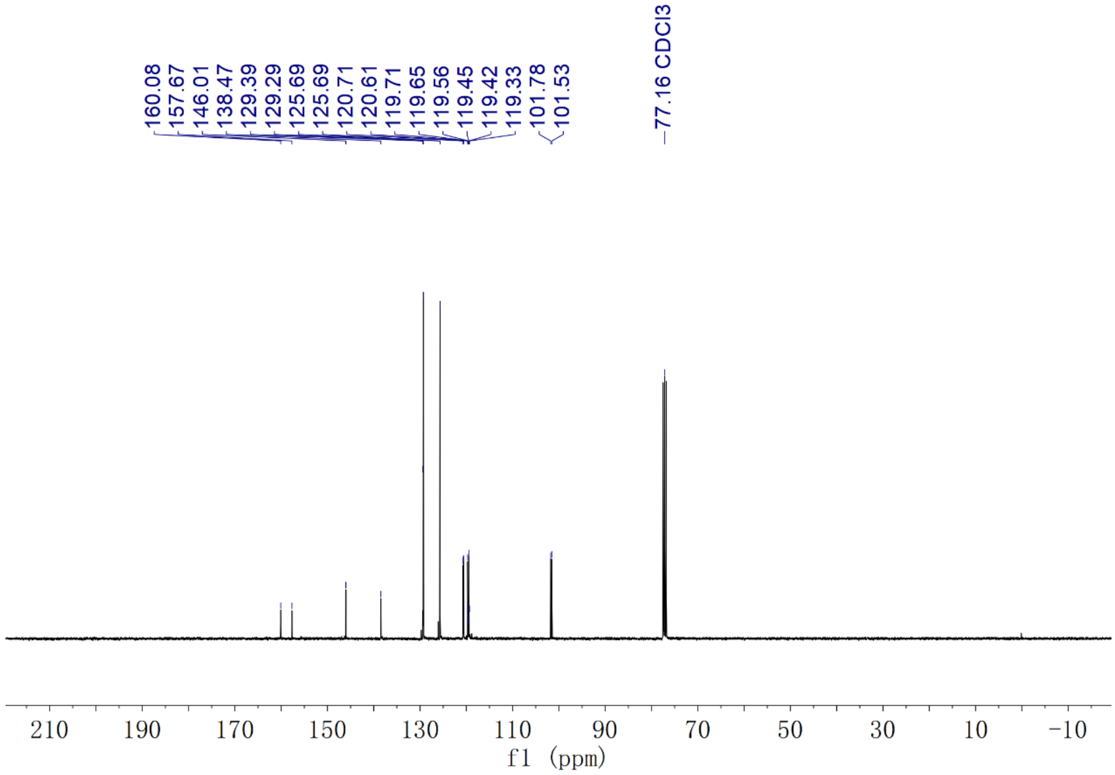

5q ^{19}F-NMR

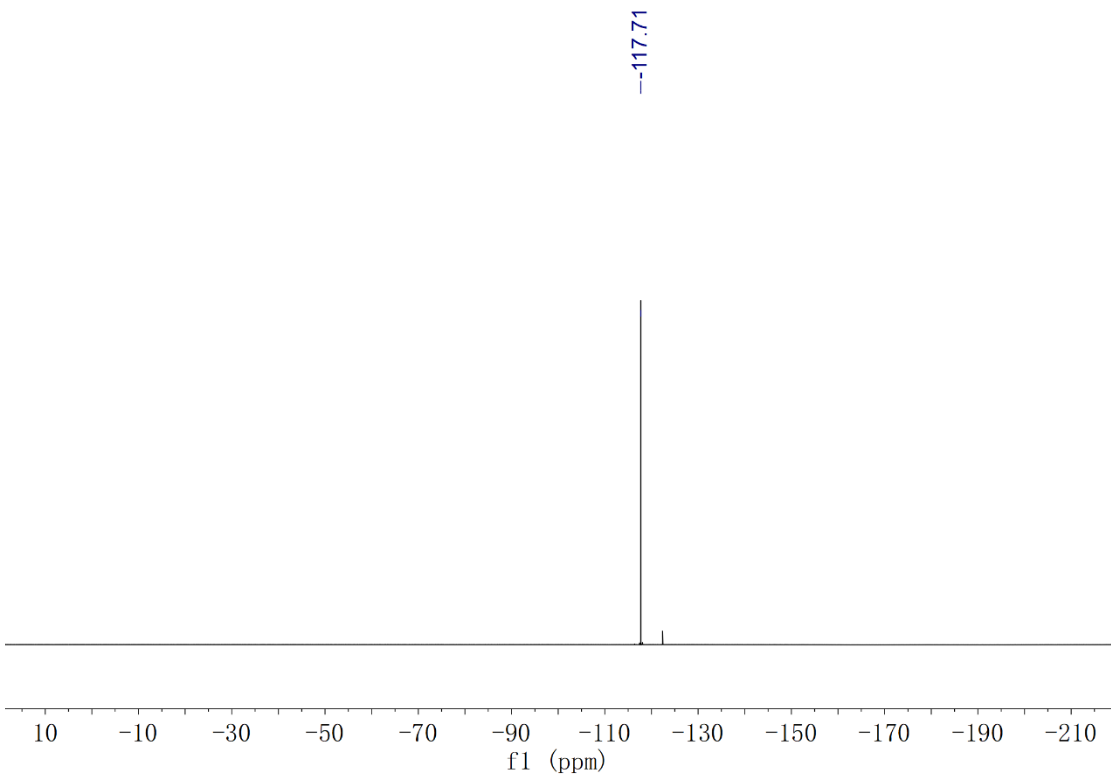

5r ^{1}H-NMR

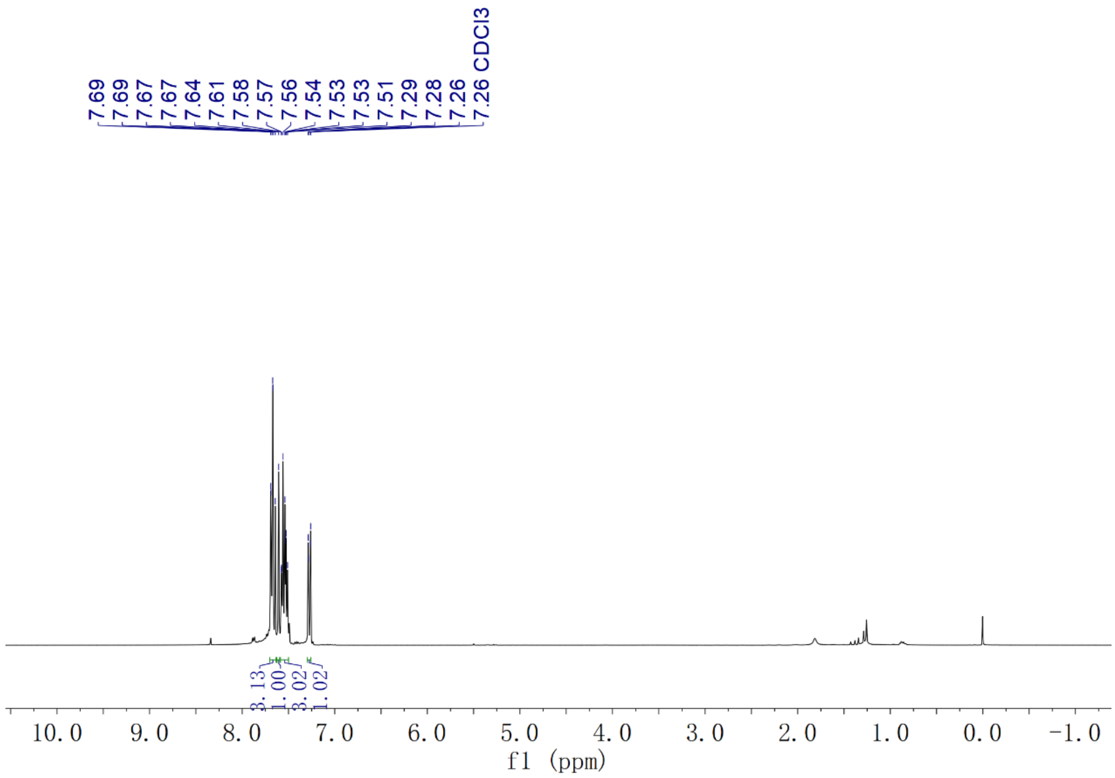

5r ^{13}C-NMR

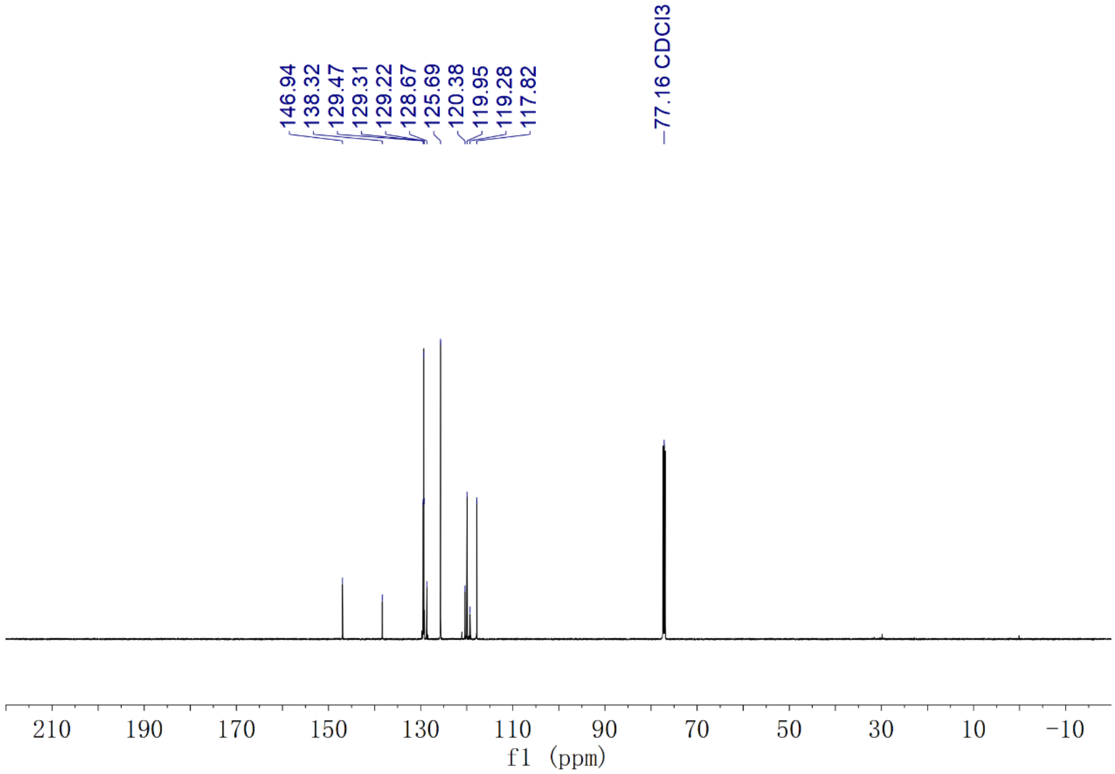

5s ^{1}H-NMR

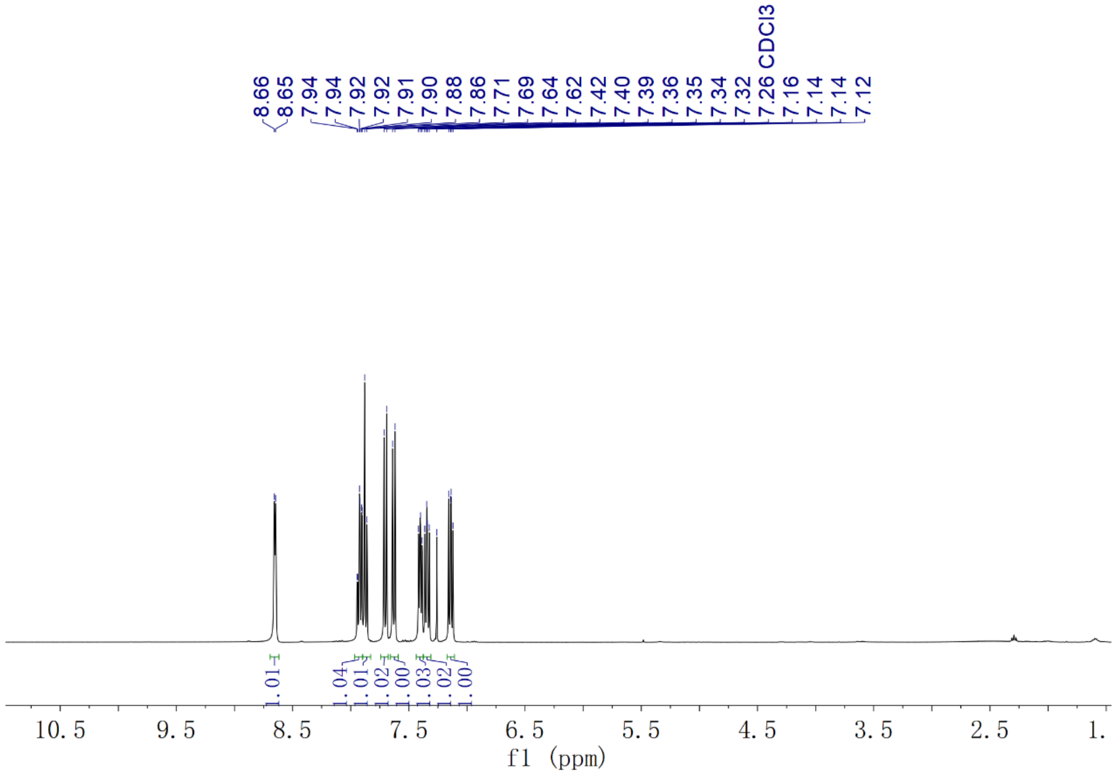

5s ^{13}C-NMR

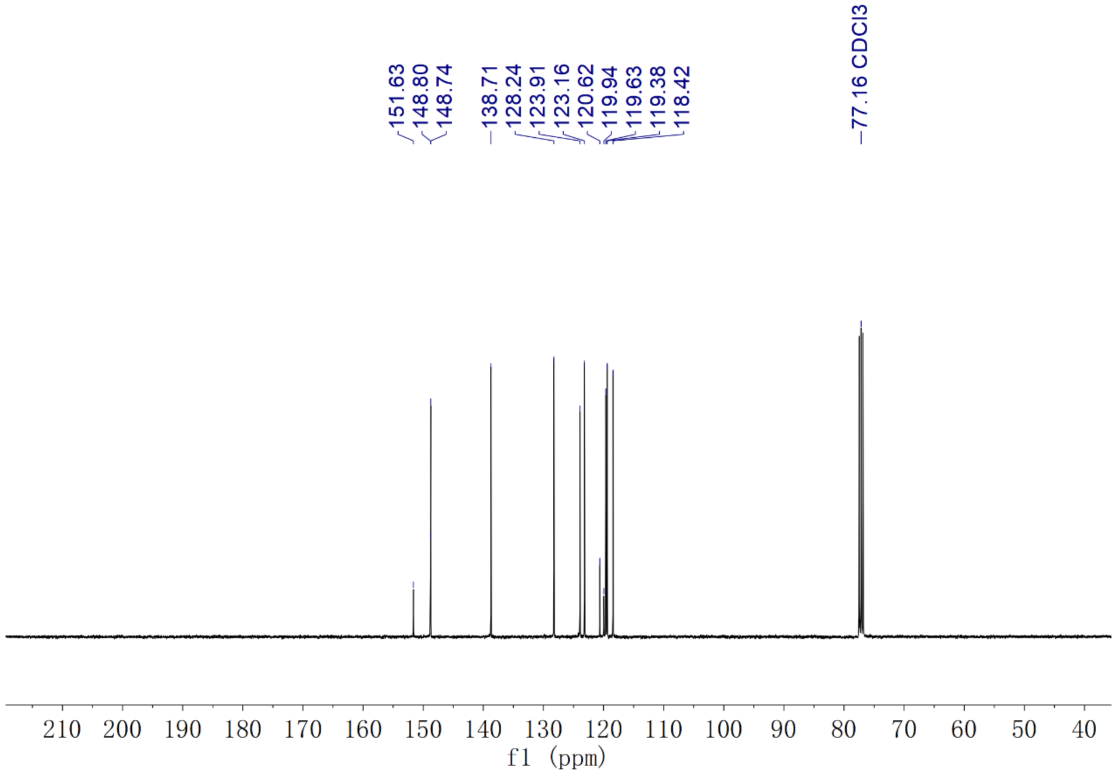

5t ^{1}H-NMR

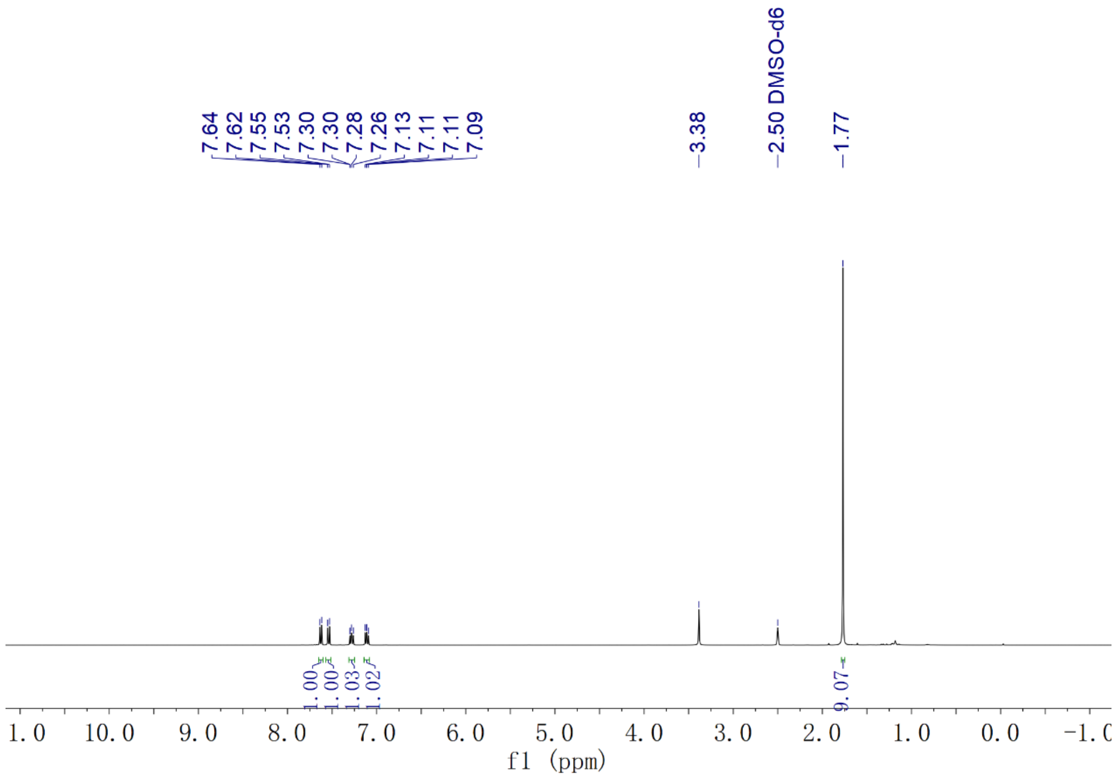

5t ^{13}C-NMR

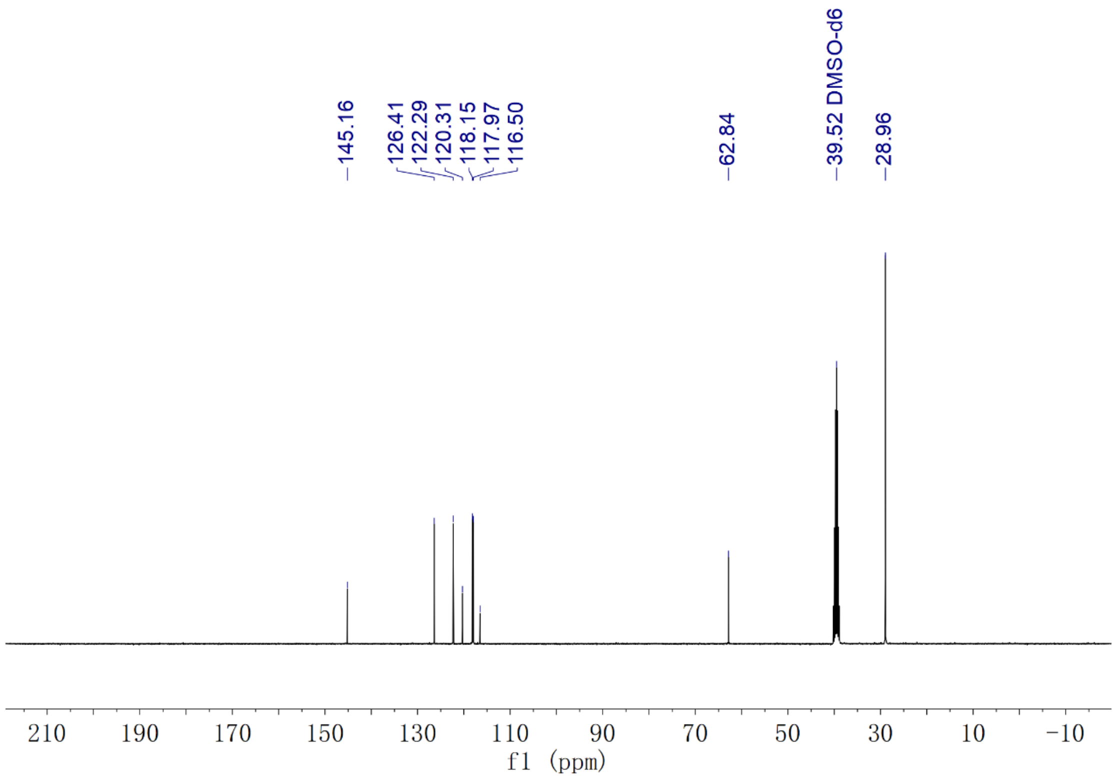

3a ^{1}H-NMR

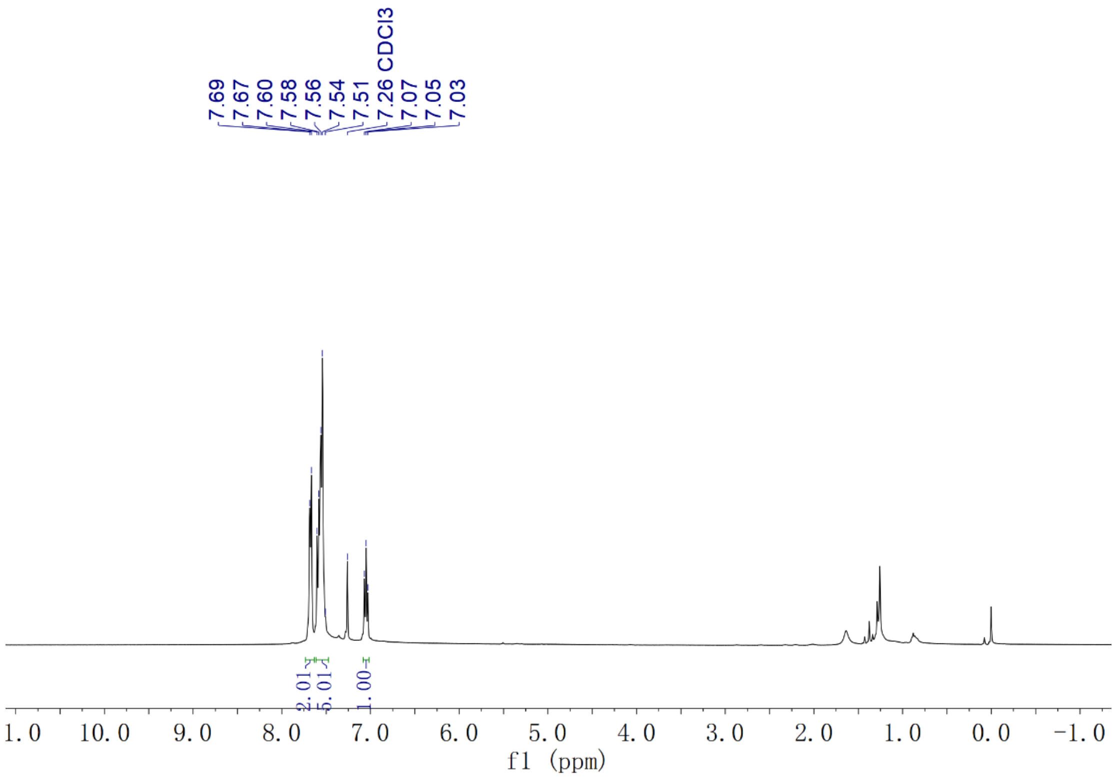

3a ^{13}C-NMR

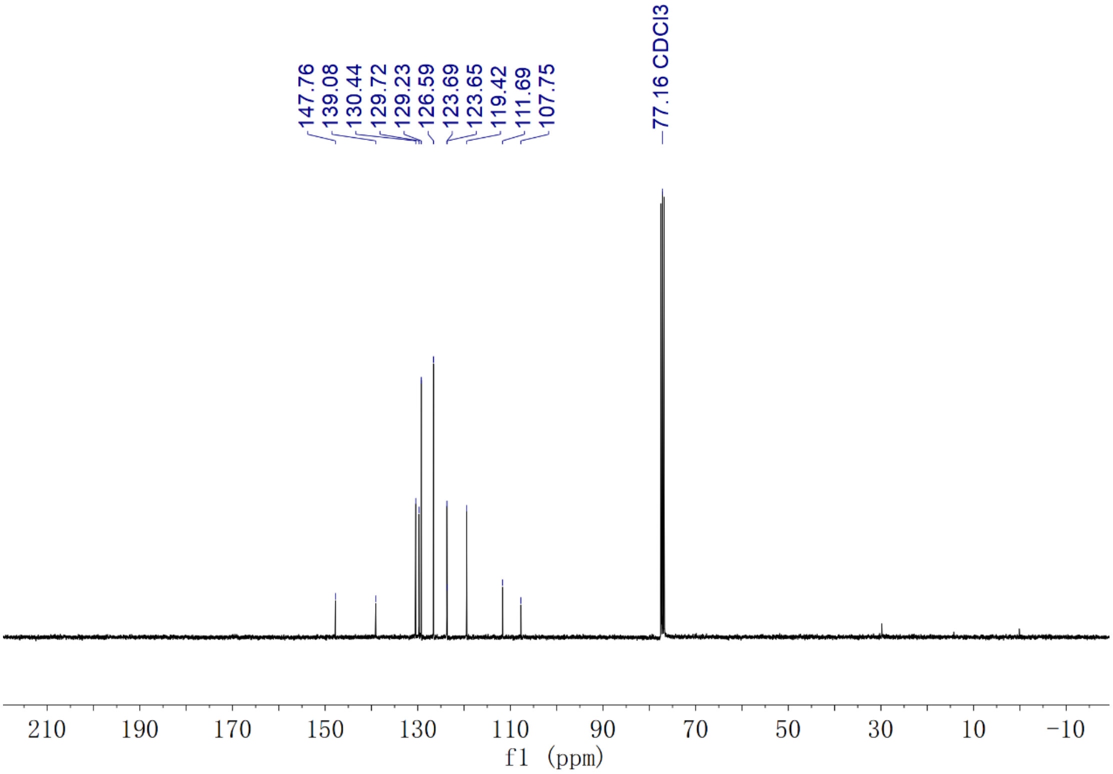

3b ^{1}H-NMR

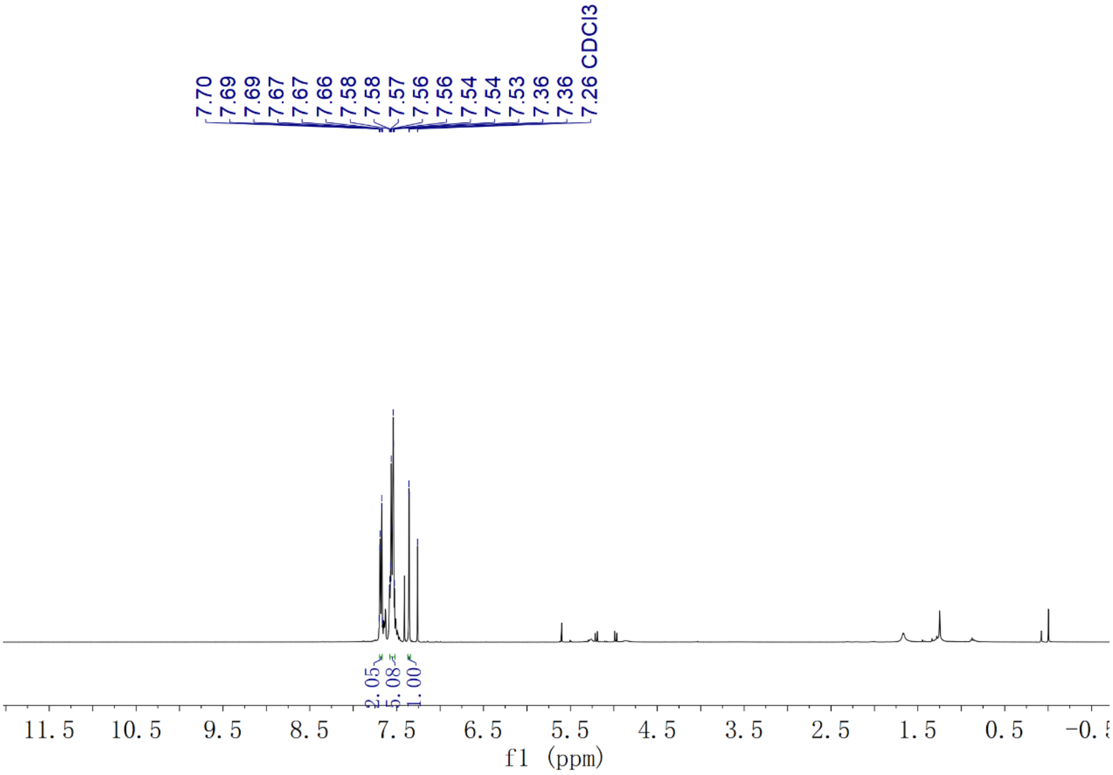

3b ^{13}C-NMR

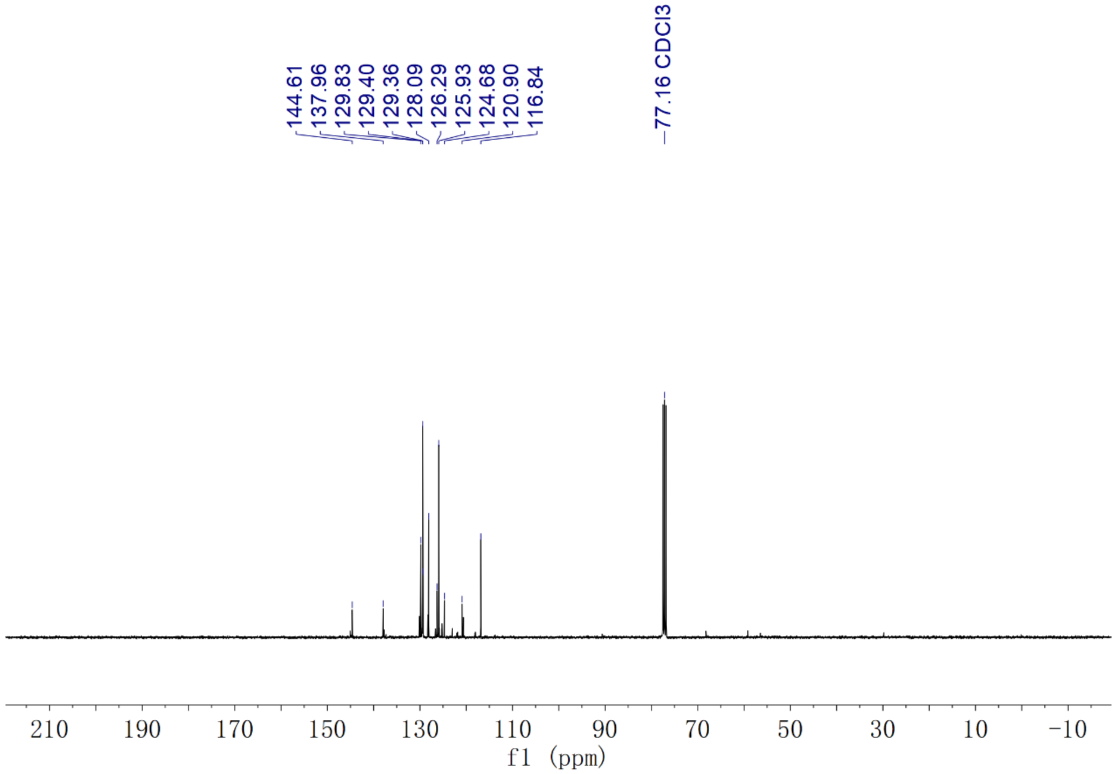

3c ^{1}H-NMR

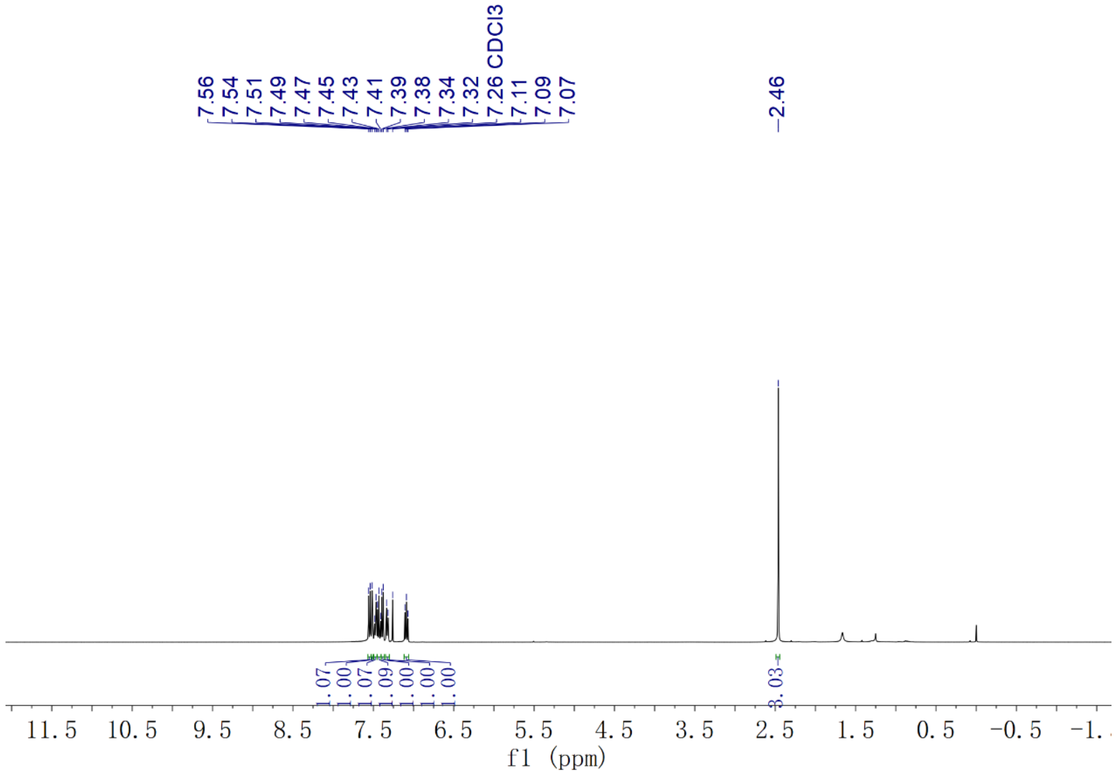

3c ^{13}C-NMR

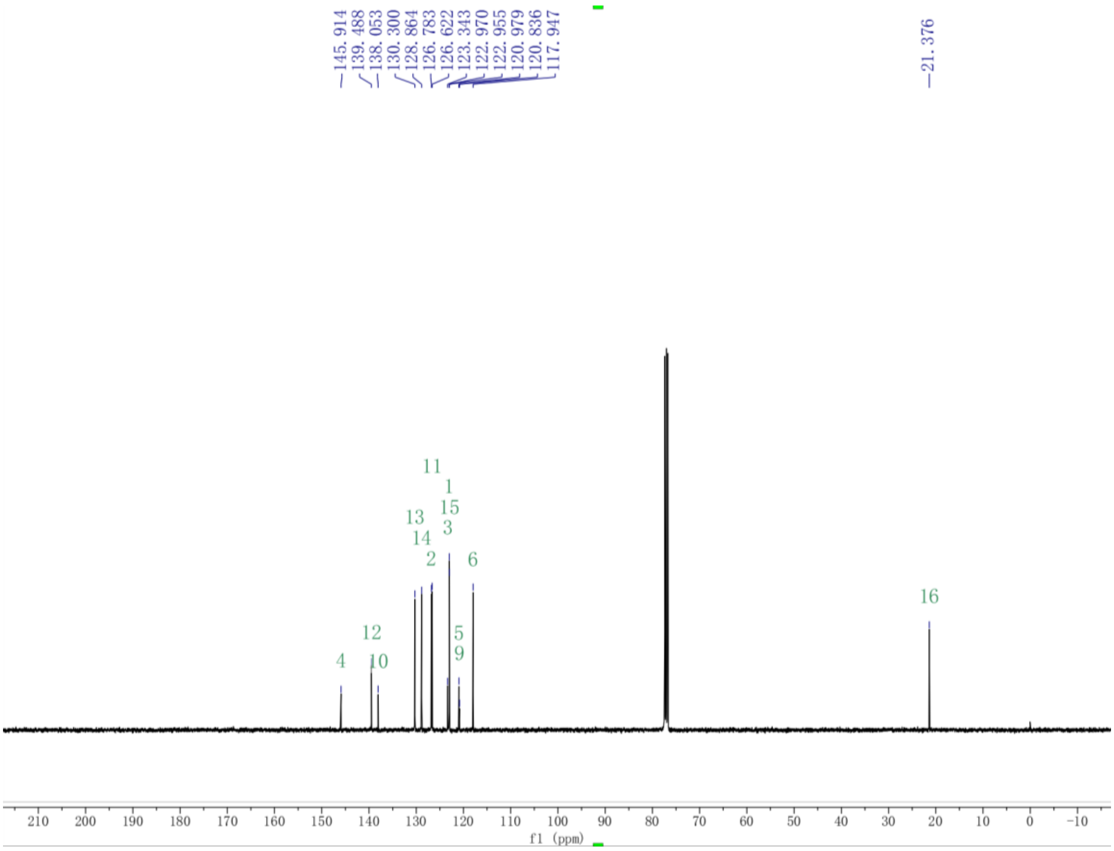

3c DEPT135

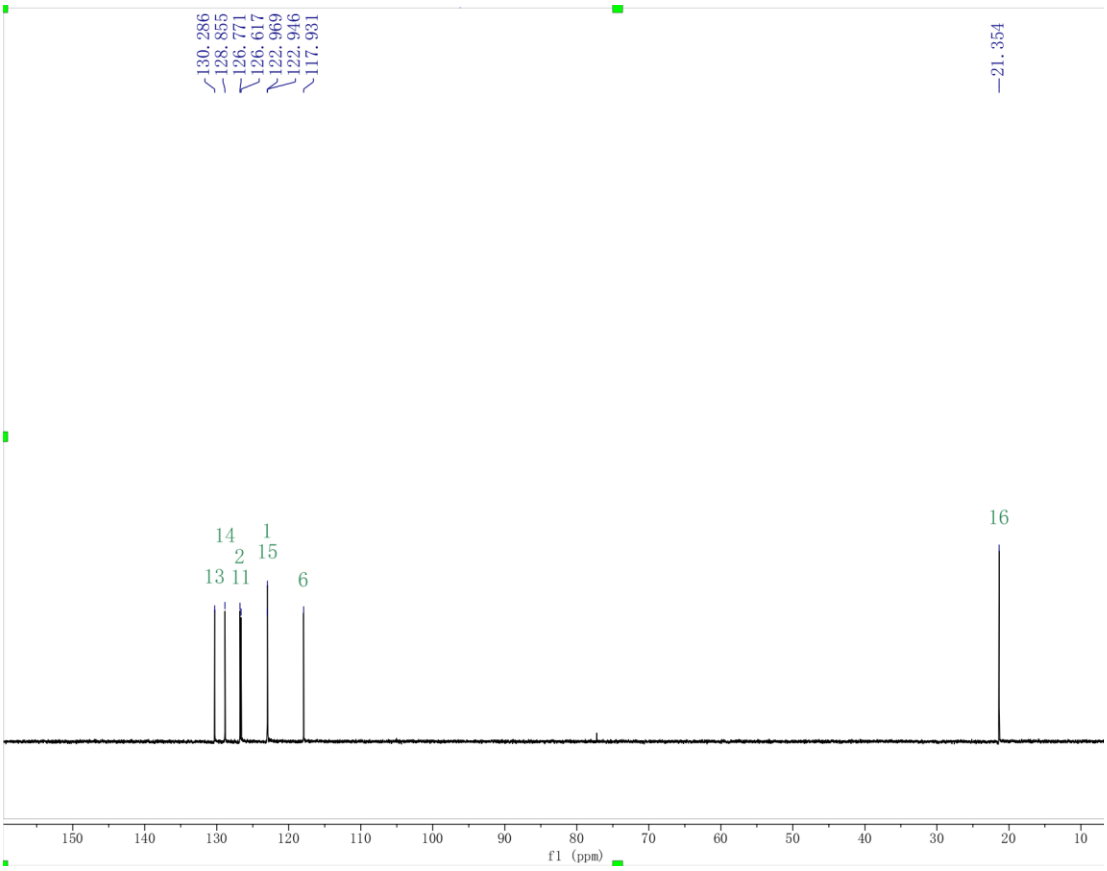

3c ^{1}H–^{1}H COSY

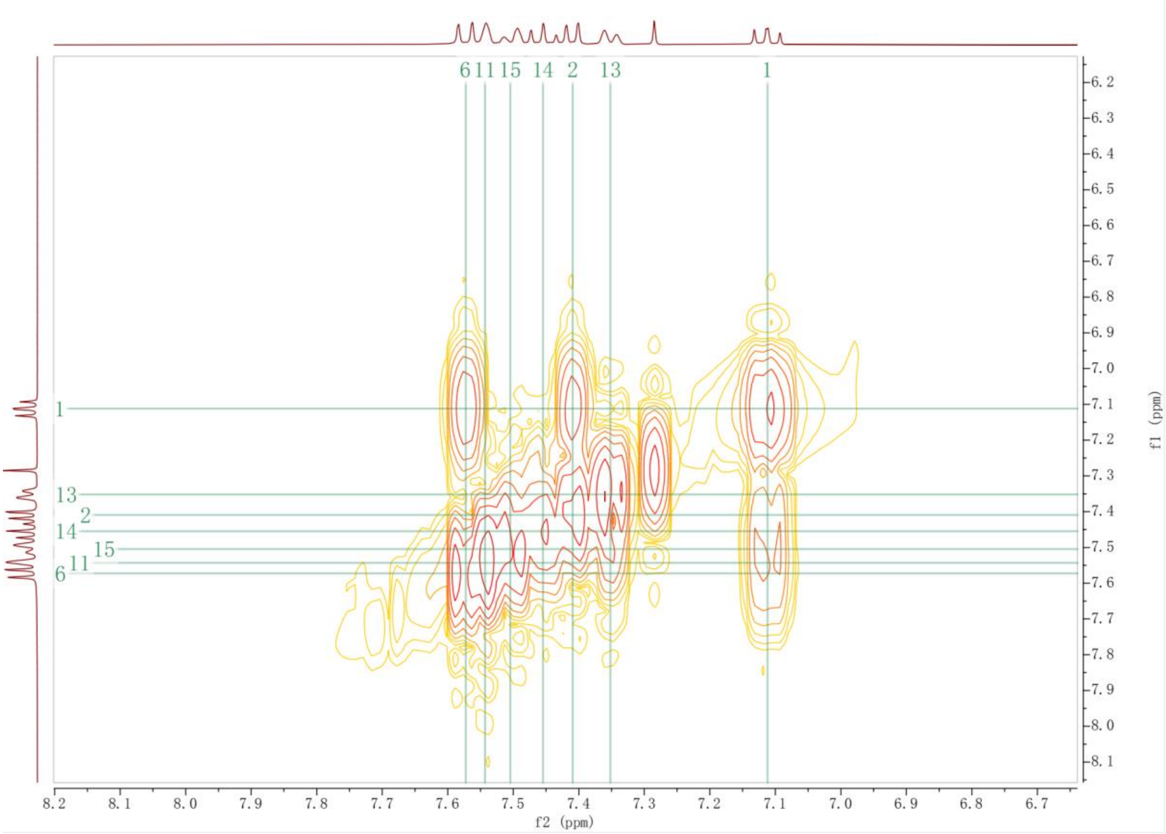

3c ^{1}H–^{13}C HSQC

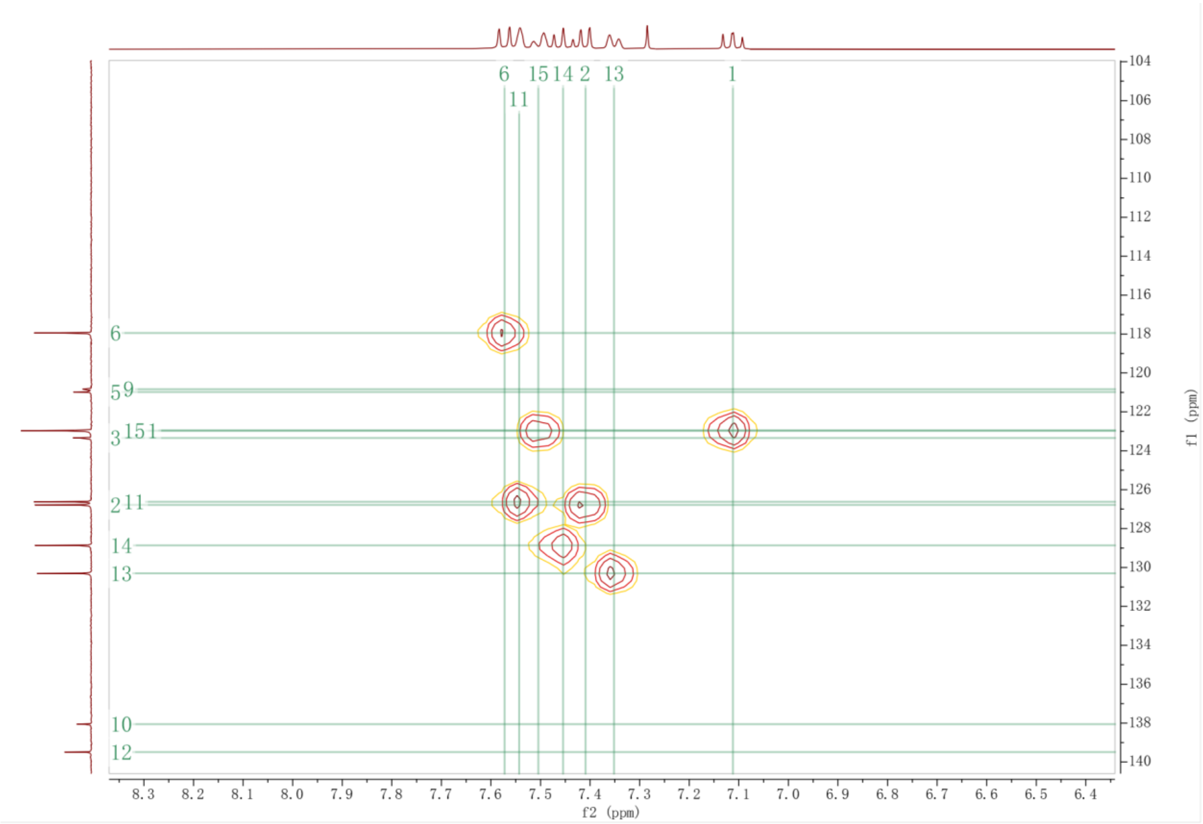

3c ^{1}H–^{13}C HMBC

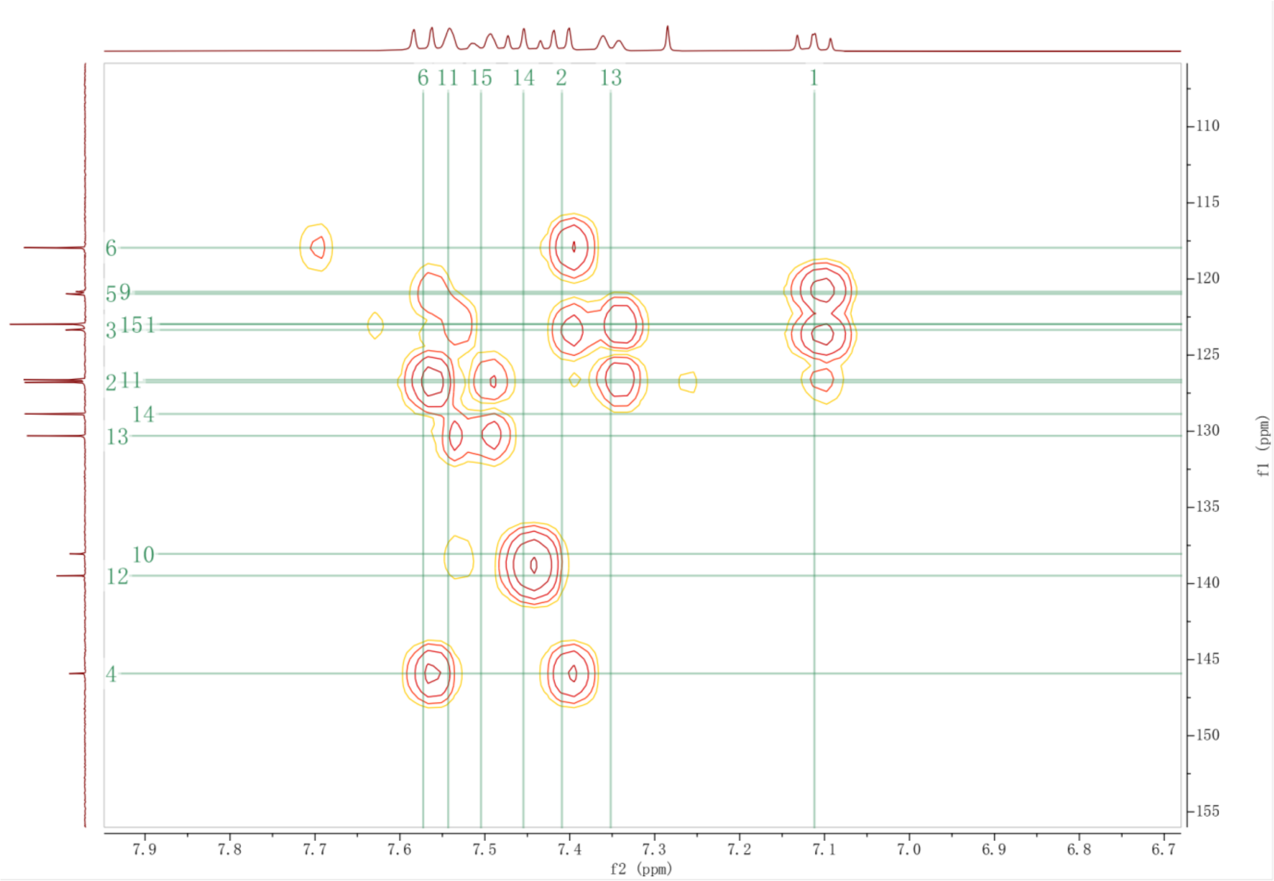

3d ^{1}H-NMR

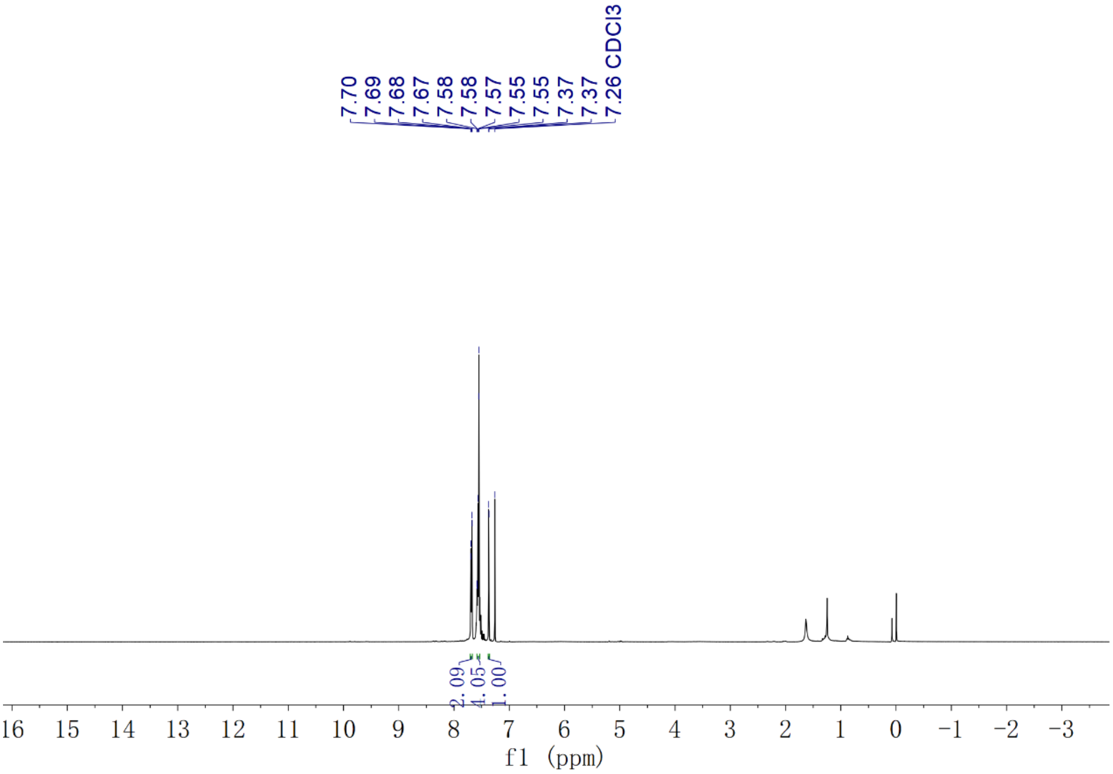

3d ^{13}C-NMR

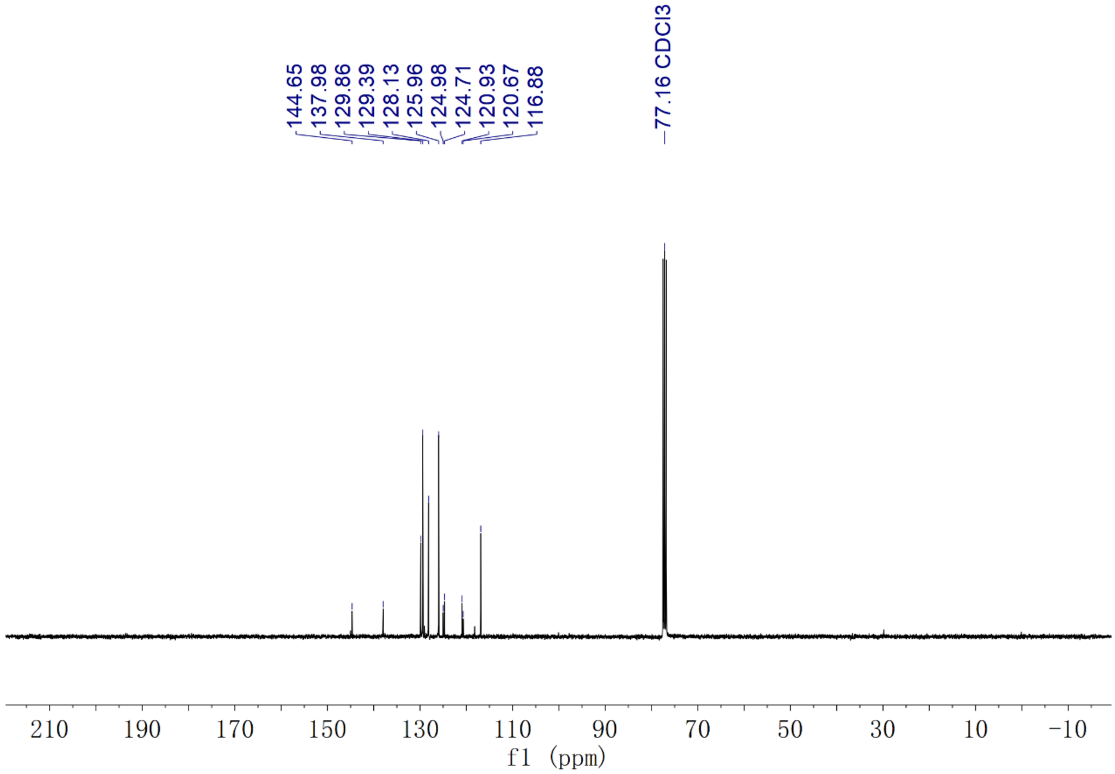

3e ^{1}H-NMR

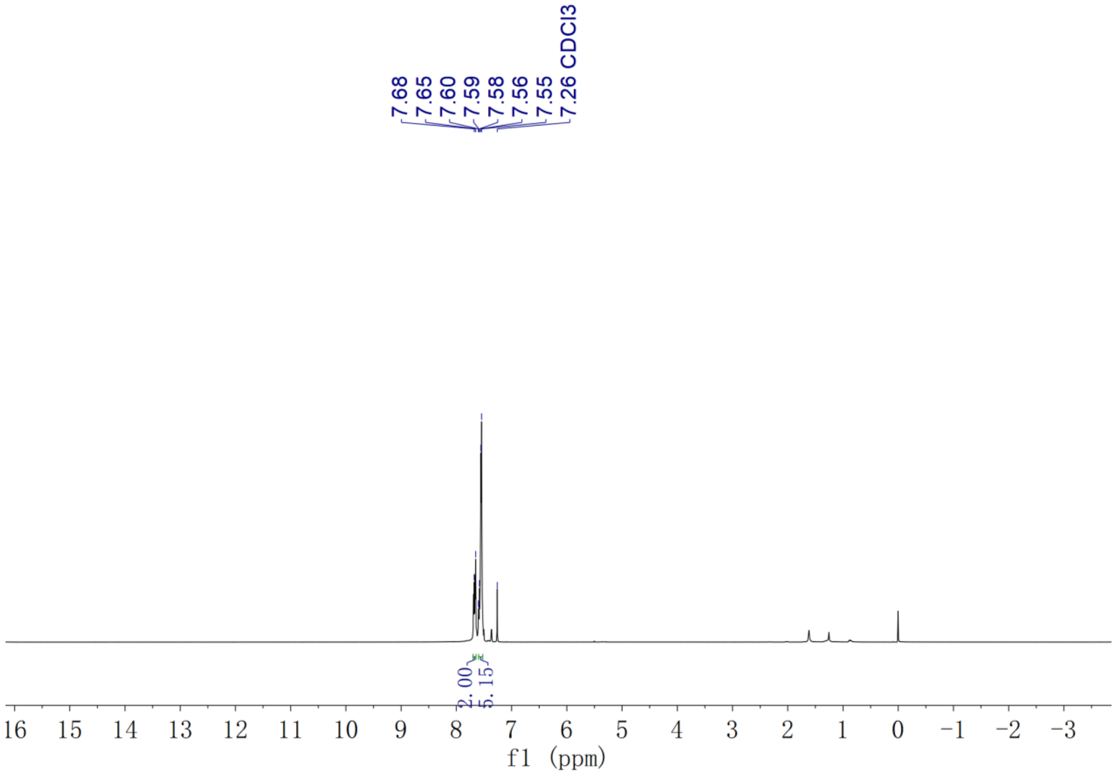

3e ^{13}C-NMR

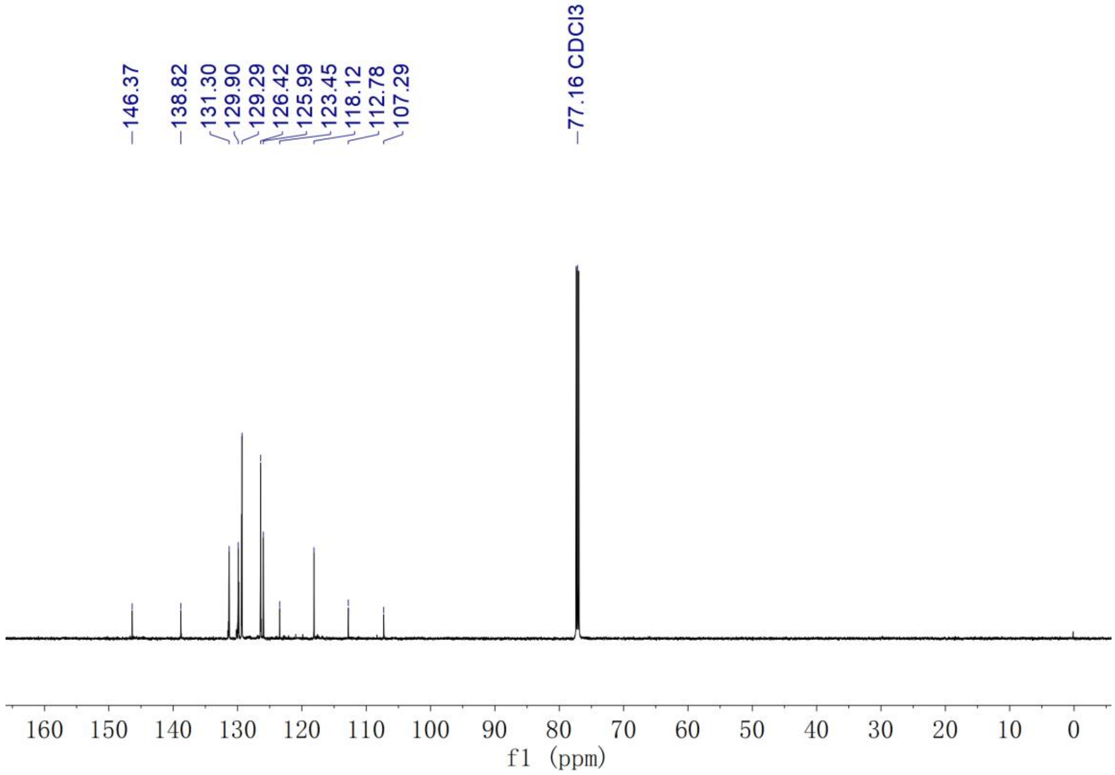

3f ^{1}H-NMR

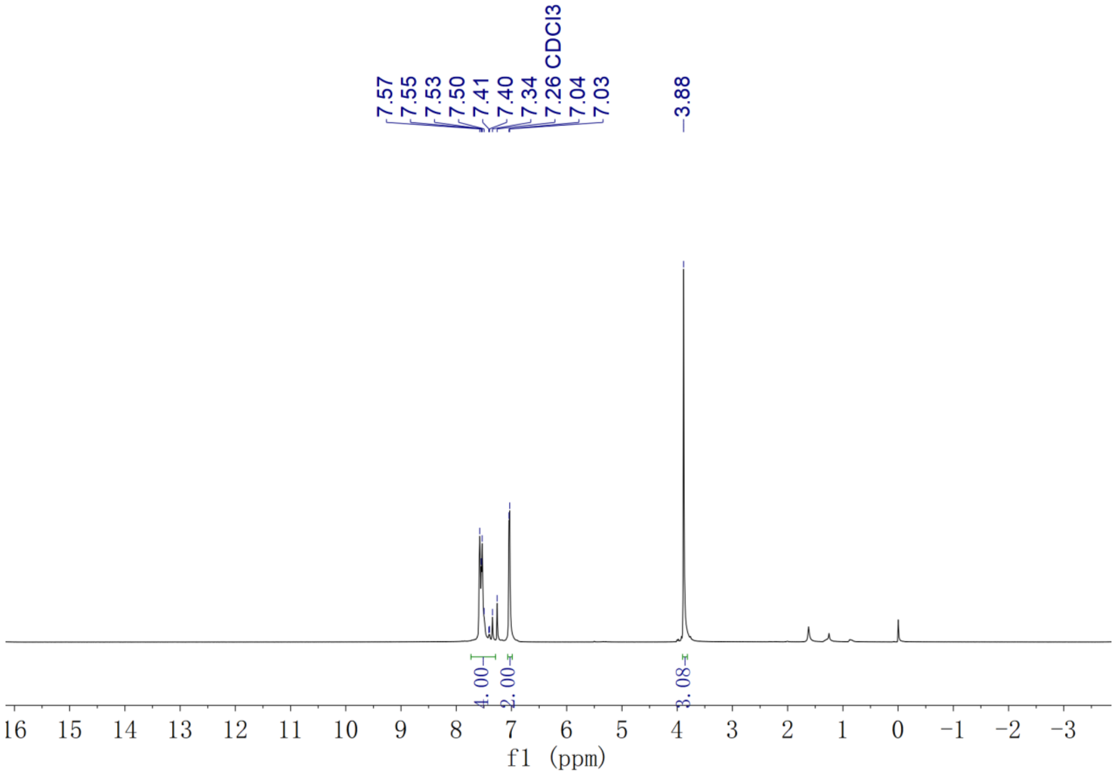

3f ^{13}C-NMR

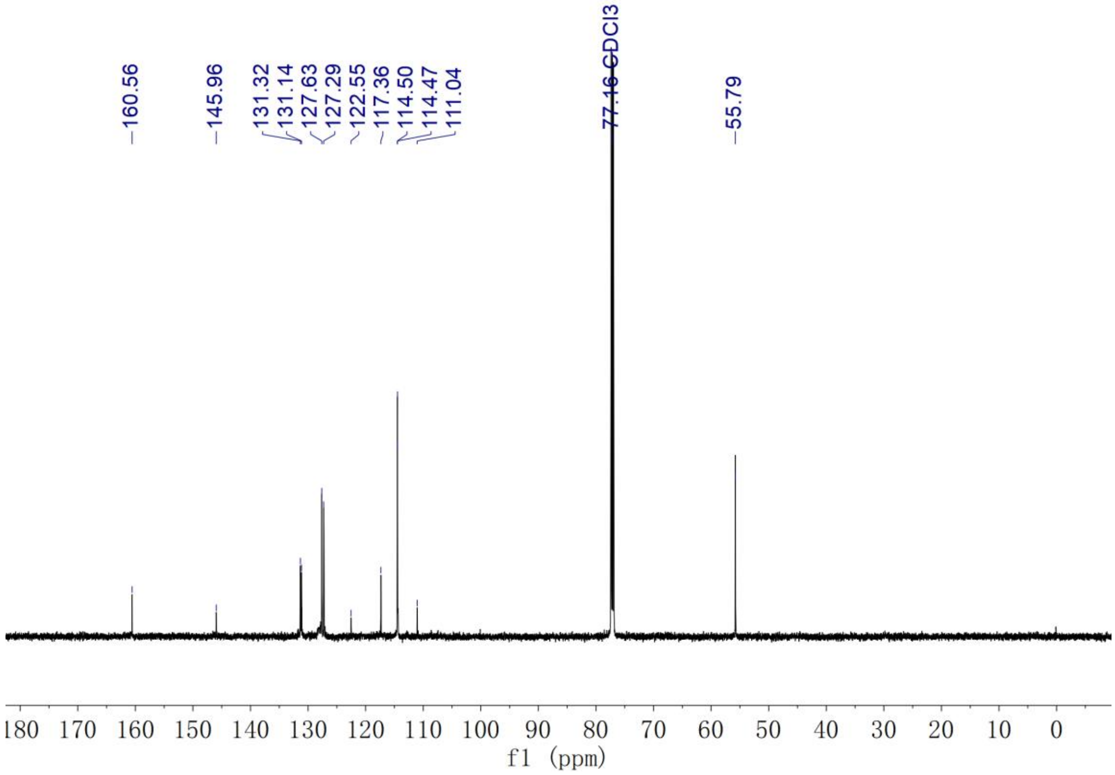

3g ^{1}H-NMR

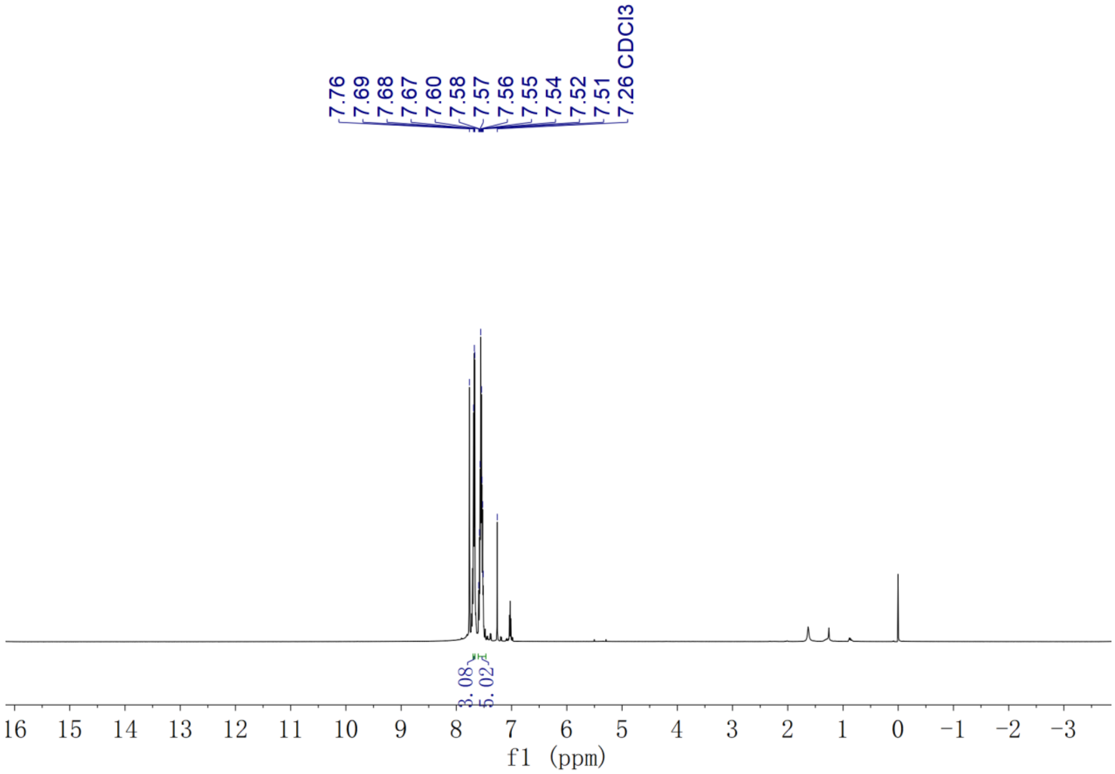

3g ^{13}C-NMR

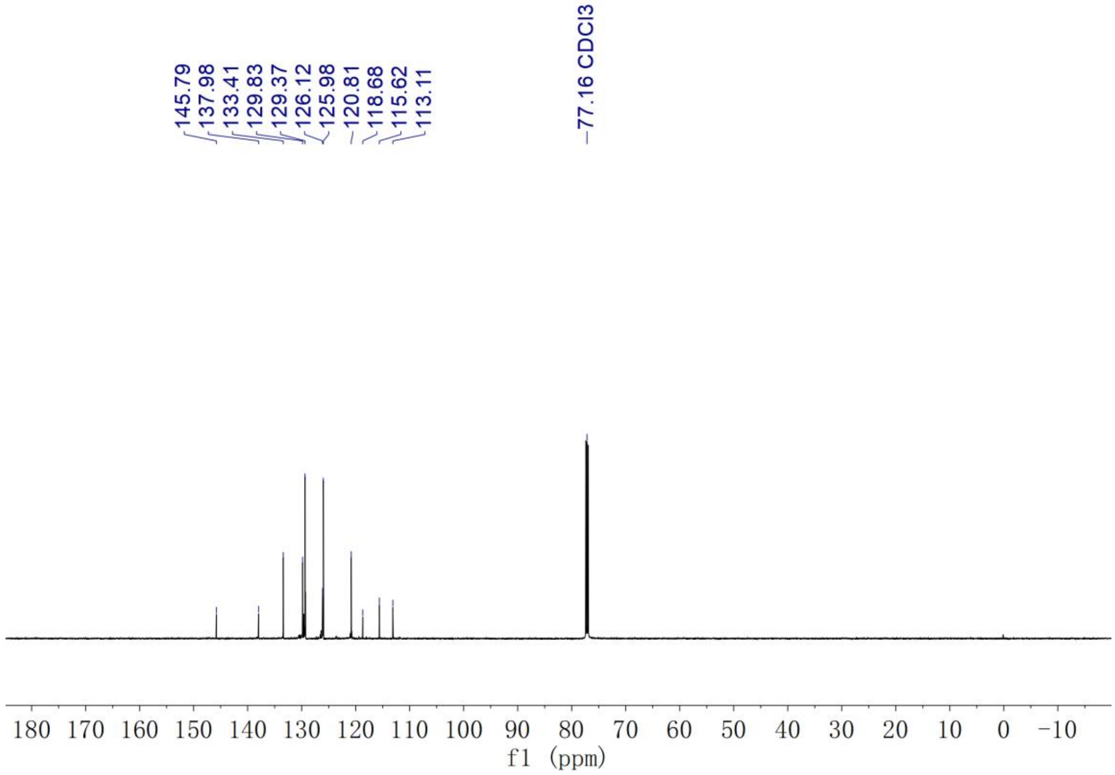

3h ^{1}H-NMR

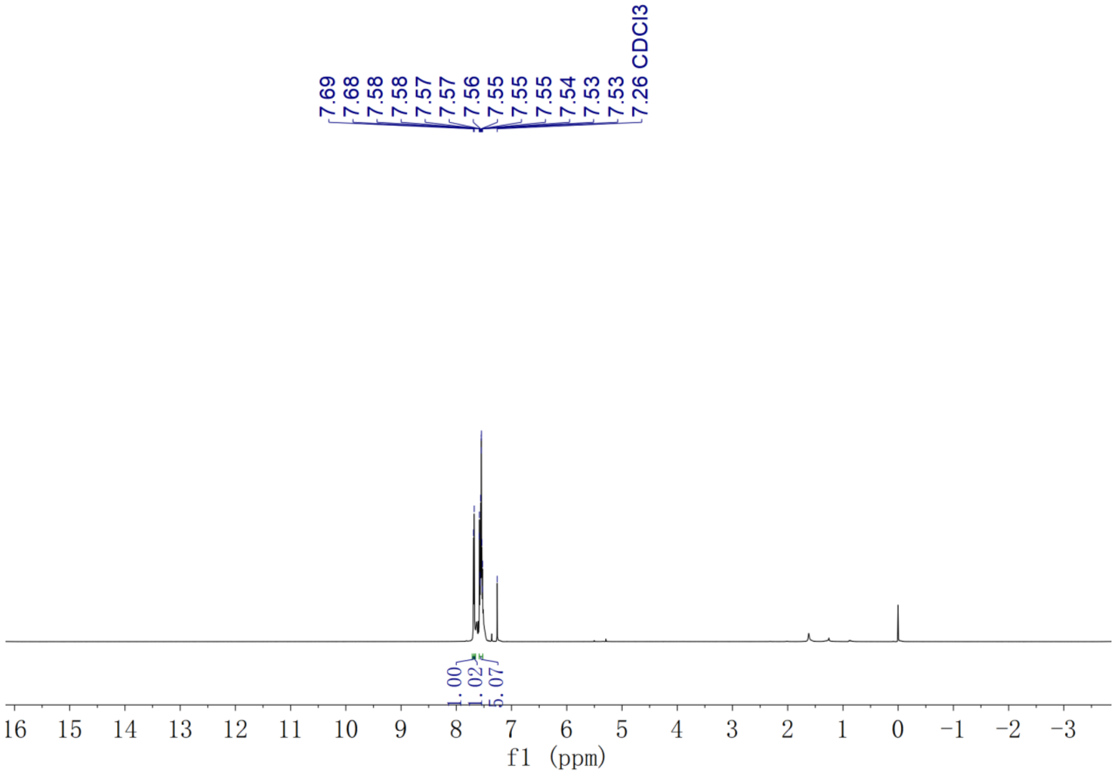

3h ^{13}C-NMR

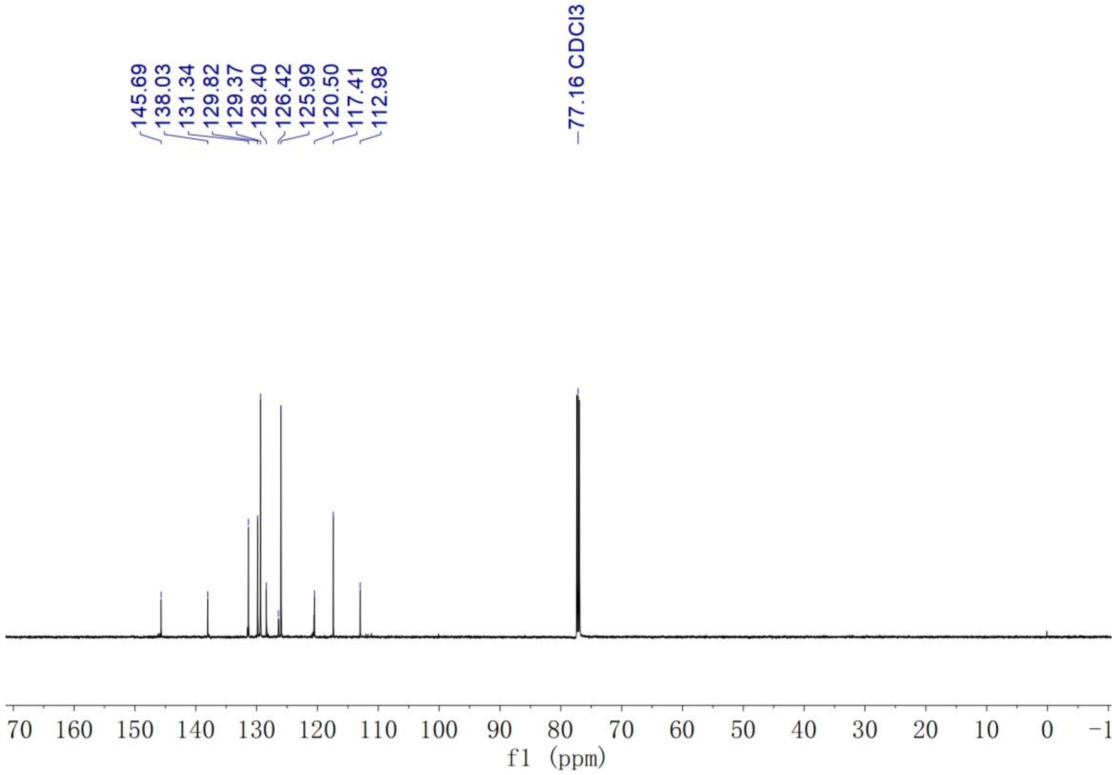

3i ^{1}H-NMR

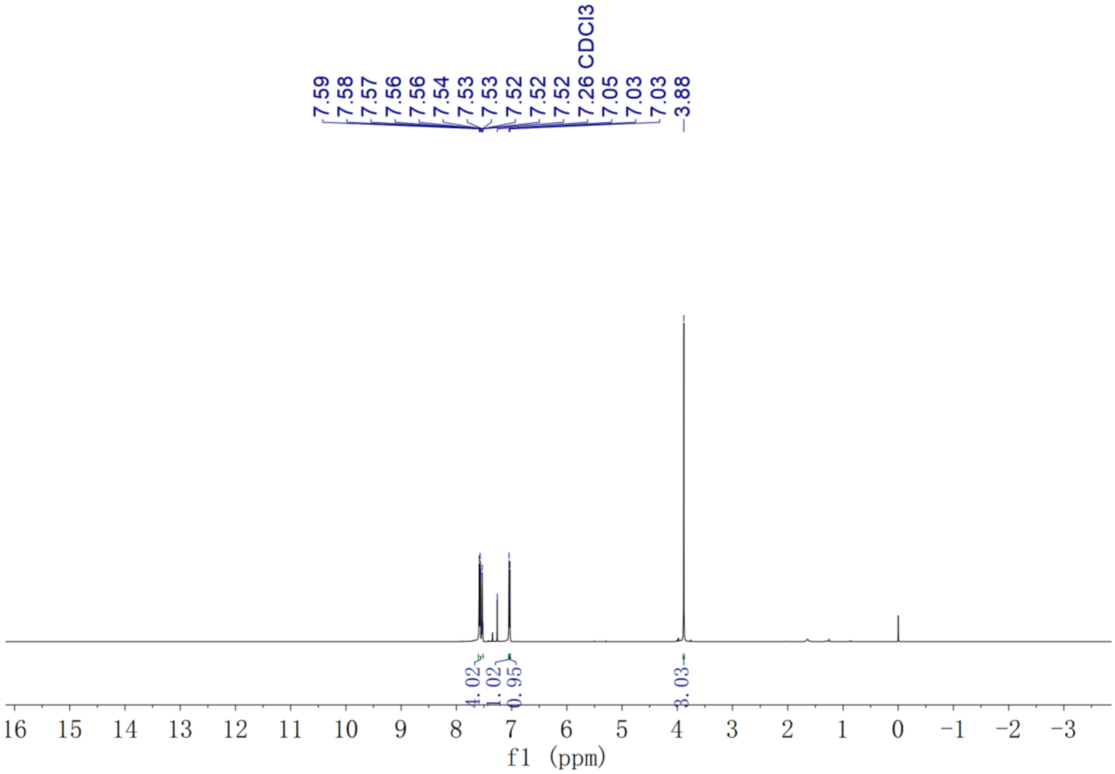

3i ^{13}C-NMR

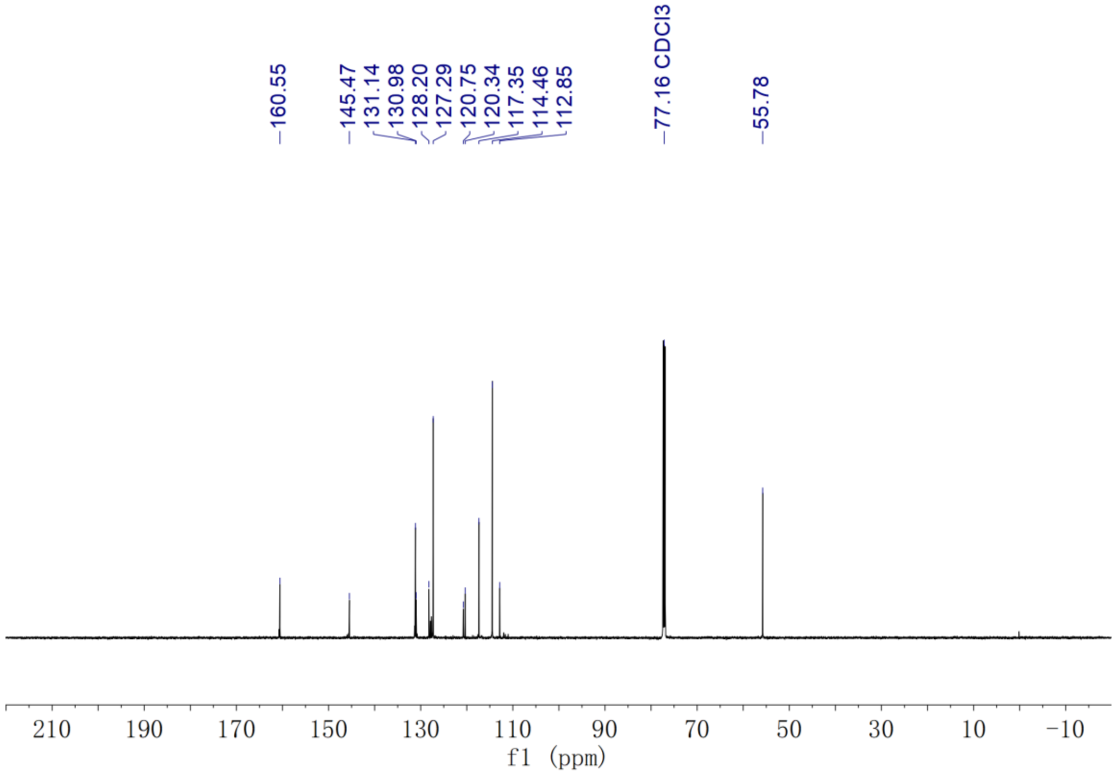

3j ^{1}H-NMR

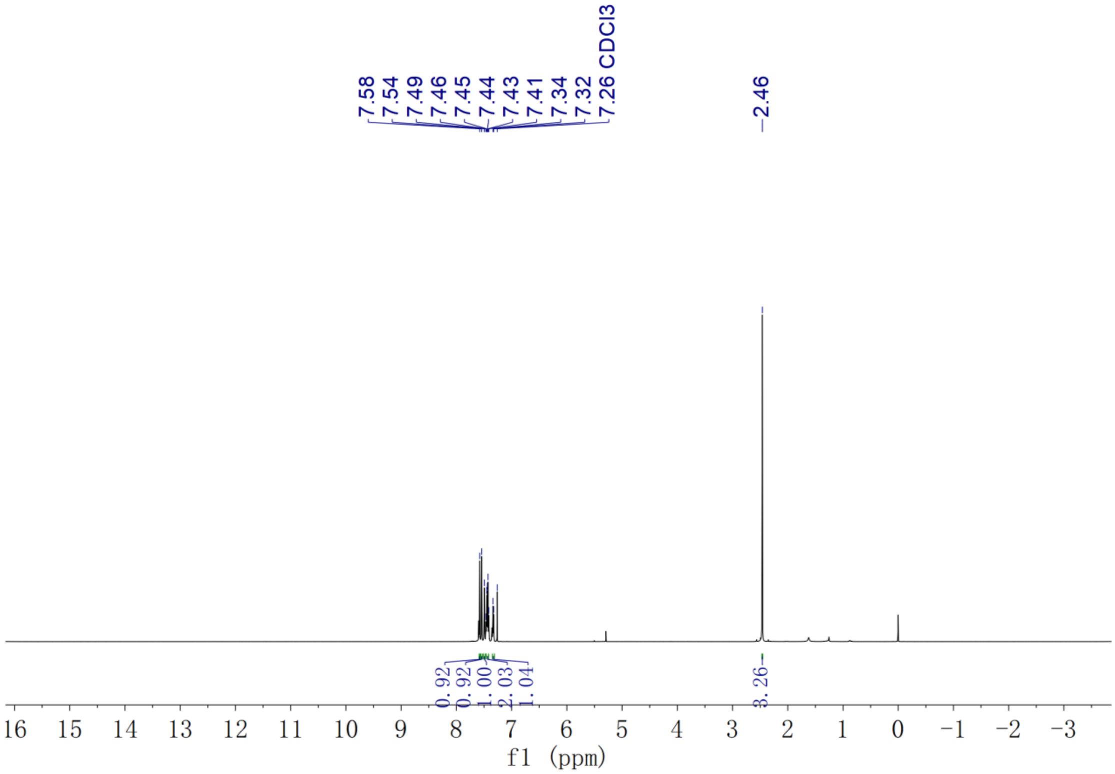

3j ^{13}C-NMR

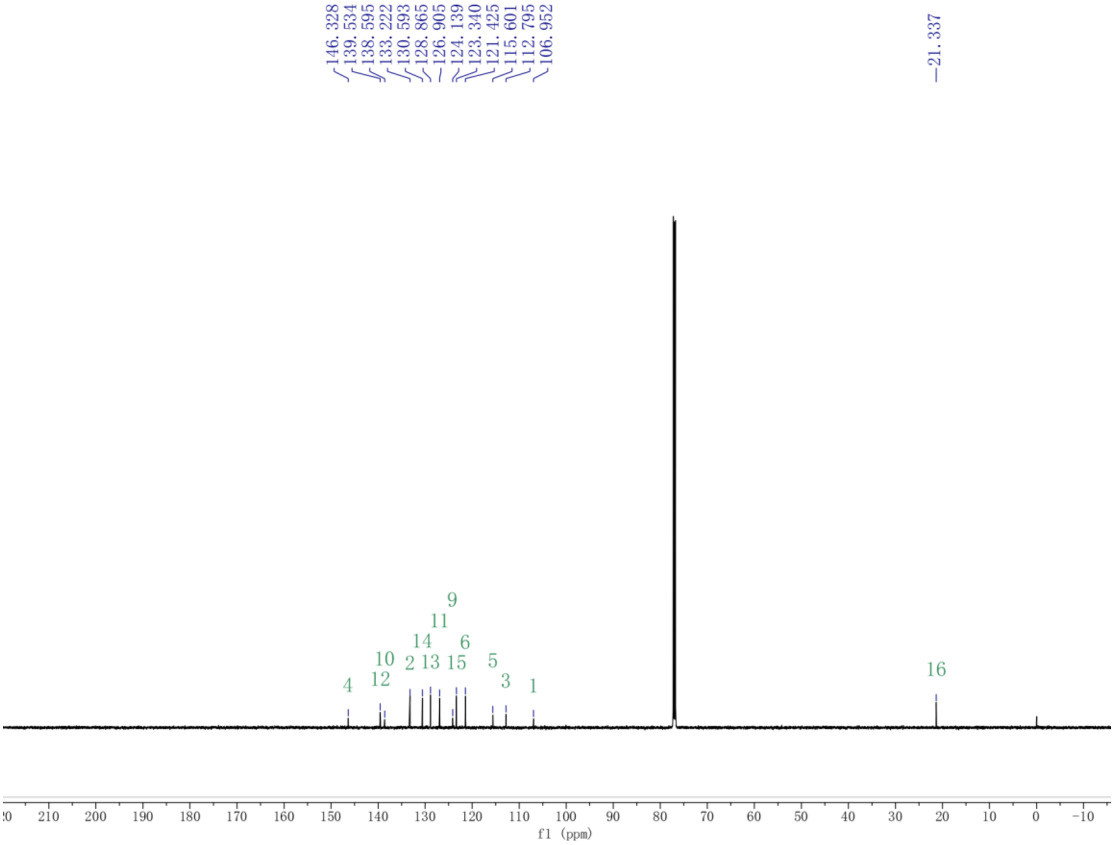

3j DEPT135

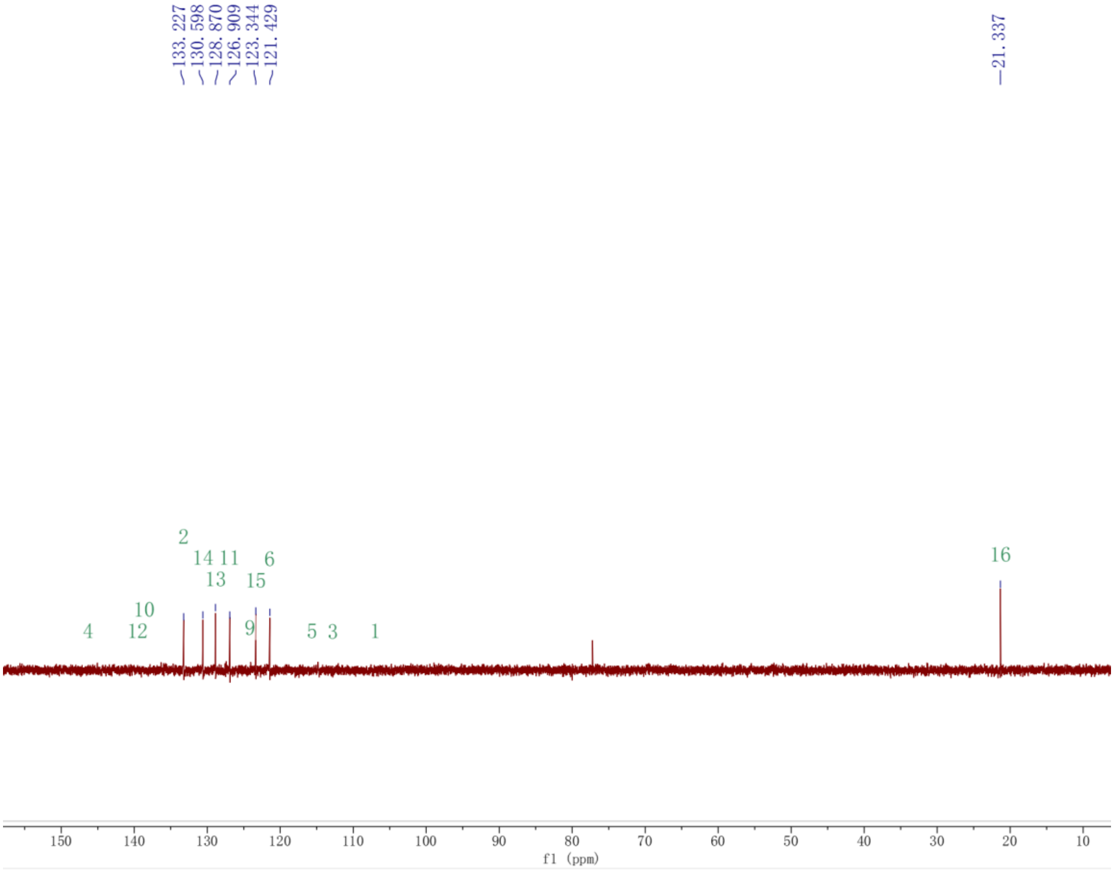

3j ^1H–^1H COSY

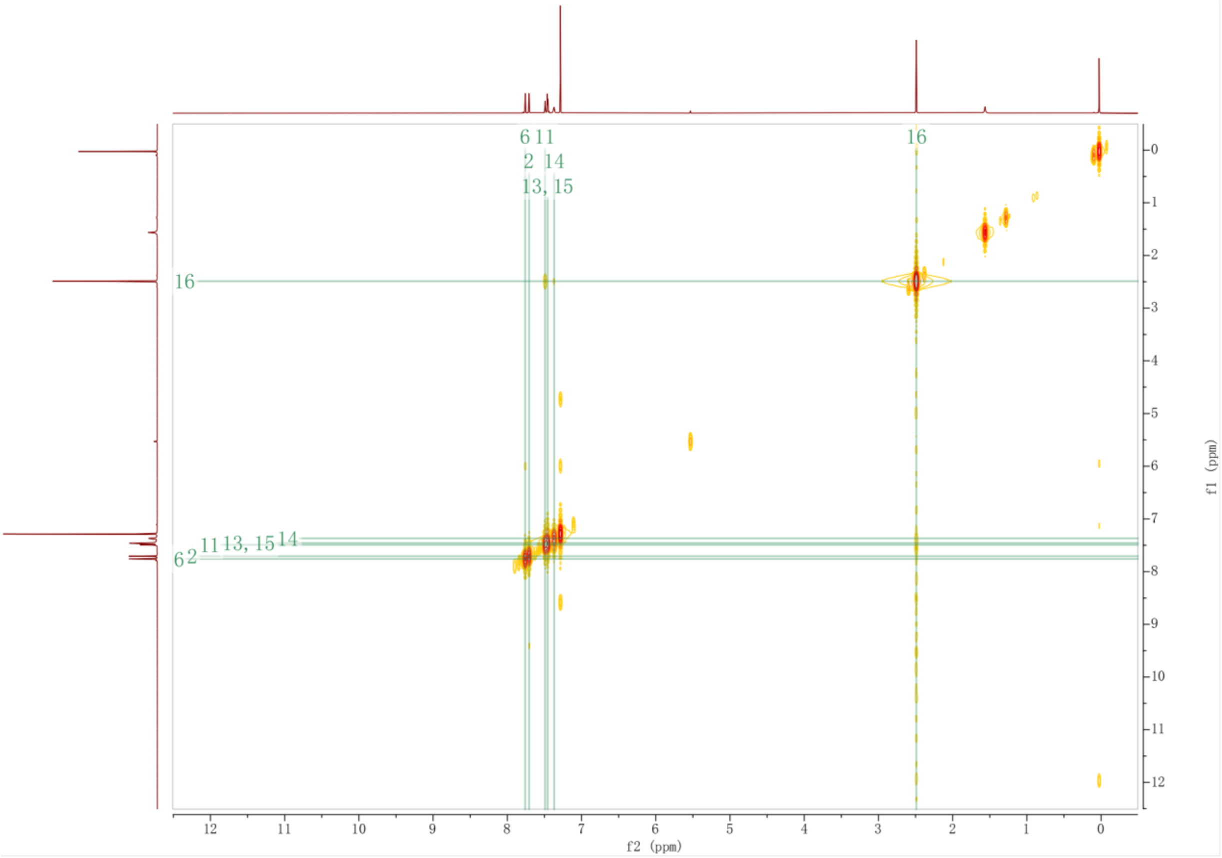

3j ^1H–^{13}C HSQC

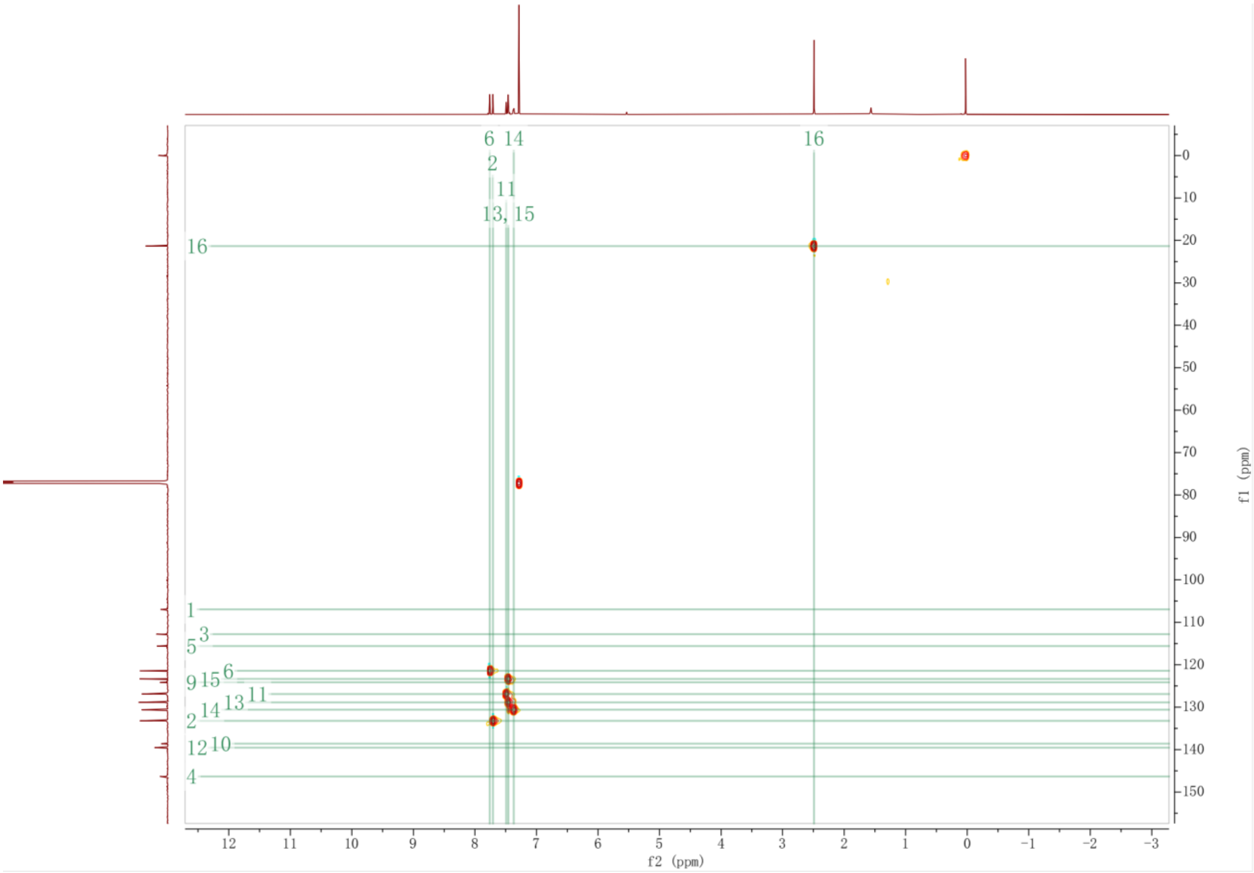

3j ^{1}H–^{13}C HMBC

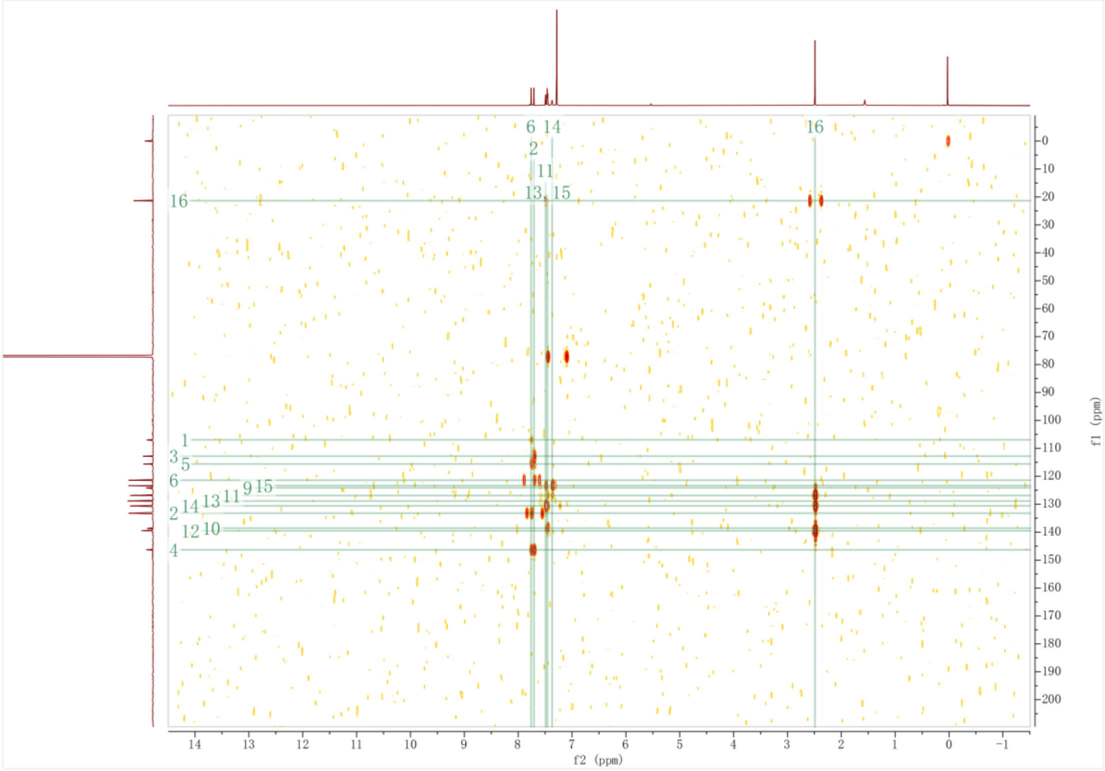

4b ^{1}H-NMR

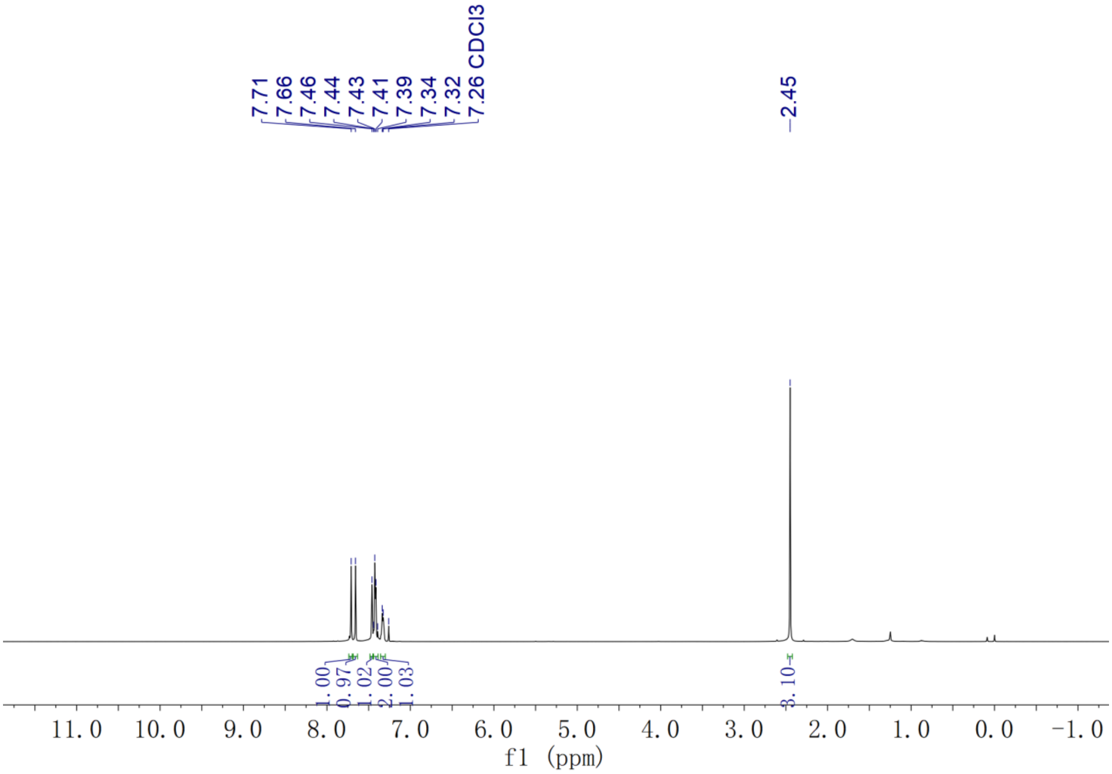

4b ^{13}C-NMR

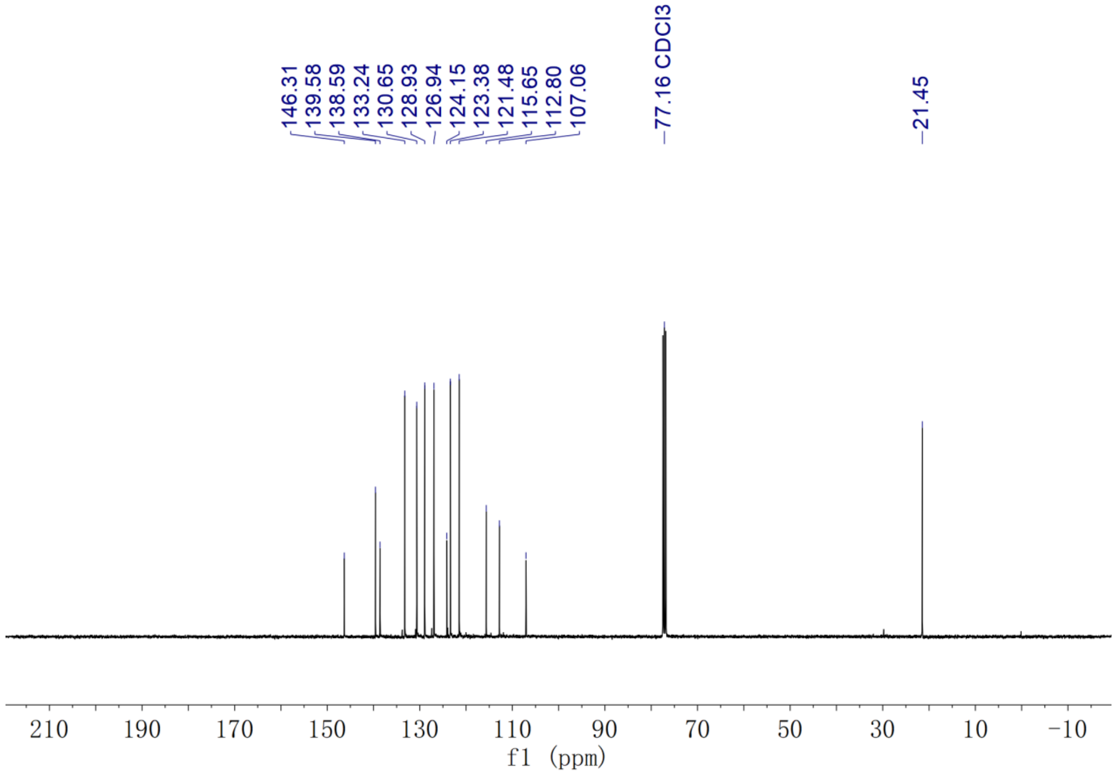

146.31
139.58
138.59
133.24
130.65
128.93
126.94
124.15
123.38
121.48
115.65
112.80
107.06
77.16 CDCl3
21.45
210
190
170
150
130
110
90
70
50
30
10
−10
f1 (ppm)
